# Transcription factor-based direct conversion of human fibroblasts to functional astrocytes

**DOI:** 10.1016/j.stemcr.2022.05.015

**Published:** 2022-06-23

**Authors:** Ella Quist, Francesco Trovato, Natalia Avaliani, Oskar G. Zetterdahl, Ana Gonzalez-Ramos, Marita G. Hansen, Merab Kokaia, Isaac Canals, Henrik Ahlenius

**Affiliations:** 1 Lund University, Faculty of Medicine, Department of Clinical Sciences Lund, Neurology, Stem Cells, Aging and Neurodegeneration, Lund, Sweden; 2 Lund Stem Cell Center, Lund, Sweden; 3 Lund University, Skane University Hospital, Department of Clinical Sciences Lund, Neurosurgery, Lund, Sweden; 4 Lund University, Faculty of Medicine, Department of Clinical Sciences Lund, Neurology, Glial and Neuronal Biology, Lund, Sweden; 5 Lund University, Skane University Hospital, Department of Clinical Sciences Lund, Epilepsy Center, Lund, Sweden

**Keywords:** direct conversion, astrocytes, transcription factors, neurons, transdifferentiation, Sox9, Nfib, Nfia, fibroblasts, neurodegenerative diseases

## Abstract

Astrocytes are emerging key players in neurological disorders. However, their role in disease etiology is poorly understood owing to inaccessibility of primary human astrocytes. Pluripotent stem cell-derived cells fail to mimic age and due to their clonal origin do not mimic genetic heterogeneity of patients. In contrast, direct conversion constitutes an attractive approach to generate human astrocytes that capture age and genetic diversity. We describe efficient direct conversion of human fibroblasts to functional induced astrocytes (iAs). Expression of the minimal combination *Sox9* and *Nfib* generates iAs with molecular, phenotypic, and functional properties resembling primary human astrocytes. iAs could be obtained by conversion of fibroblasts covering the entire human lifespan. Importantly, iAs supported function of induced neurons obtained through direct conversion from the same fibroblast population. Fibroblast-derived iAs will become a useful tool to elucidate the biology of astrocytes and complement current *in vitro* models for studies of late-onset neurological disorders.

## Highlights

• Effective direct conversion of human fibroblasts to induced astrocytes (iAs)
• iAs resemble human primary astrocytes at molecular, phenotypic, and functional levels
• iAs can be generated from fibroblasts covering the entire human lifespan
• iAs support function of induced neurons obtained from the same starting population

## Graphical abstract

**Figure:**
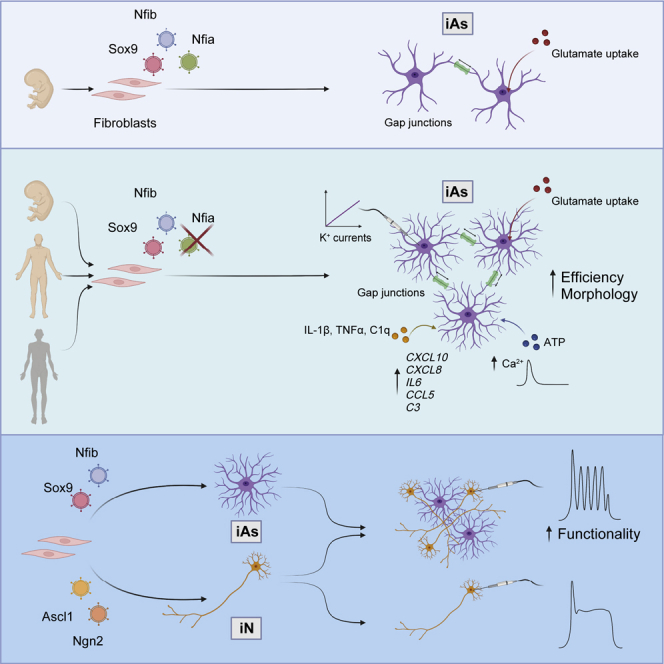

## Teaser

Generation of functional induced astrocytes (iAs) from human fibroblasts has potential to become very important for studies on sporadic and age-related neurological disorders. Here, Quist, Ahlenius and colleagues describe direct conversion of fibroblasts, derived throughout the human lifespan, to functionally mature astrocytes. Furthermore, generation of iAs enabled functional co-cultures of induced neurons and astrocytes from the same starting fibroblast population.

## Introduction

Astrocytes are a major cell type in the mammalian central nervous system (CNS) and are crucial for brain functionality (Molofsky et al., 2012). They provide trophic and functional support to neurons, take up and recycle neurotransmitters, and modulate neurotransmission (Allen and Eroglu, 2017). Astrocytes maintain brain homeostasis, form functional syncytia, communicate intra- and intercellularly, and respond to injury and inflammation (Verkhratsky and Nedergaard, 2018).

With these essential roles, it is not surprising that astrocyte dysfunction is associated with a range of neurological disorders. However, there is poor understanding of molecular roles of astrocytes and their interplay with other CNS cells in disease contexts (Molofsky et al., 2012). Traditionally, rodents have been used to study astrocyte biology but interspecies differences raise concerns regarding translational potential to humans (Zhang et al., 2016). Another hurdle is limited accessibility of astrocytes from living healthy individuals and patients with neurological diseases. However, recent advances in stem cell technology have emerged to generate human astrocytes for disease modeling.

Recently, we developed efficient generation of functional astrocytes from human pluripotent stem cells (Canals et al., 2018). However, for modeling aging, sporadic, or late-onset diseases, direct conversion protocols bypassing pluripotency are needed, as they preserve genetic mosaicism of the starting population and retain age-related features (Mertens et al., 2016). In addition, direct conversion avoids ethical concerns regarding embryonic stem cells and circumvents the need for reprogramming to pluripotency, making direct conversion an easy and faster approach to obtain patient-derived astrocytes. However, generation of induced astrocytes (iAs) by direct conversion from human fibroblasts has only been briefly explored (Caiazzo et al., 2015; Tian et al., 2016) remains inefficient and functional characterization is scarce.

Here, we describe substantially improved generation of iAs from human fibroblasts obtained from individuals covering the entire lifespan. In addition, we demonstrate that iAs are phenotypically and functionally similar to native human astrocytes. We also show for the first time functional co-culture of iAs and induced neurons (iNs) obtained from the same human starting fibroblast population, which constitutes a powerful tool for the study of astrocyte-neuron interactions and for disease modeling.

## Results

### *Sox9*, *Nfia*, and *Nfib* convert human embryonic fibroblasts to induced astrocytes

Gliogenic transcription factors or small molecules have been shown to convert mouse fibroblasts to induced astrocytes (Caiazzo et al., 2015; Tian et al., 2016). However, conversion of human fibroblasts has not been studied in detail, and efficiency in generating mature functional human astrocytes remains low.

**Figure 1:**
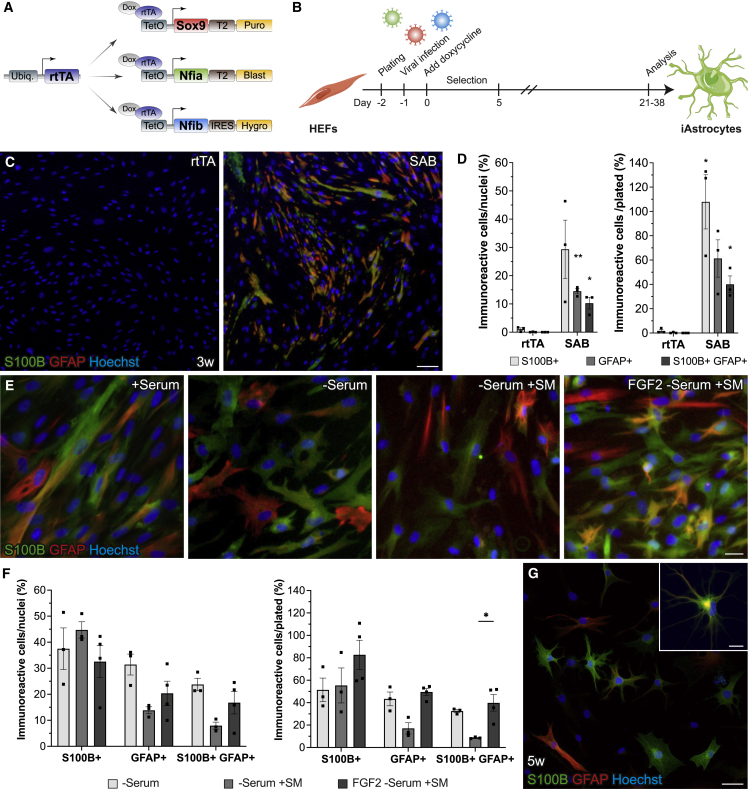
*Sox9*, *Nfia*, and *Nfib* convert human embryonic fibroblasts to induced astrocytes (A) Schematic of doxycycline-inducible lentiviral constructs. (B) Experimental overview of direct conversion to induced astrocytes (iAs). (C) Representative immunofluorescence images of GFAP and S100B in rtTA- and SAB-infected HEFs at 3 weeks. (D) Quantification of S100B, GFAP, and S100B/GFAP immunoreactive cells at 3 weeks related to total number of nuclei (purity) and plated cells (yield). (E) Representative immunofluorescence images of SAB-HEF-iAs at 3 weeks converted in serum-containing medium (+Serum), serum-free medium (−Serum), serum-free medium with small molecules (−Serum + SM), or low-serum-containing medium with SM and FGF2 followed by serum-free medium with SM (FGF2 − Serum + SM). (F) Quantification of purity and yield of S100B, GFAP, and S100B/GFAP immunoreactive cells at 3 weeks. (G) Immunofluorescence image of SAB-HEF-iAs at 5 weeks converted in FGF2 − Serum + SM medium. Puro, puromycin resistance gene; Blast, blasticidin resistance gene; Hygro, hygromycin resistance gene; HEFs, human embryonic fibroblasts. Individual color levels are adjusted for visibility in (C), (E), and (G). Data are presented as mean ± SEM of n = 3–4 independent experiments. Two-tailed paired t tests (D) and Kruskal-Wallis test (F) were performed for statistical analysis. ^∗^p < 0.05, ^∗∗^p < 0.01. Scale bars, 100 μm (C), 25 μm (E), 50 μm (G), and 20 μm (G, inset).

To improve this, we cloned cDNAs of *Sox9*, *Nfia*, and *Nfib* (SAB), able to convert mouse fibroblasts, into doxycycline-inducible lentiviral vectors containing puromycin, blasticidin, and hygromycin resistance genes, respectively (Canals et al., 2018), to enable the selection of cells with high expression of transgenes. We then infected human embryonic fibroblasts (HEFs) with a vector constitutively expressing reverse tetracycline-controlled transactivator (rtTA) alone, as control, or together with SAB vectors, induced expression using doxycycline, and the day after started a 5-day selection period (Figures 1A and 1B). Cells were kept in serum-containing medium commonly used for primary mouse astrocytes. Three weeks after induction, we performed immunocytochemistry for canonical astrocyte markers S100B and glial fibrillary acidic protein (GFAP). Strikingly, we observed around 30% and 15% of SAB-infected HEFs expressing S100B and GFAP, respectively, and 10% co-expressed S100B and GFAP, which corresponded to a yield of 110% and 60% of S100B- and GFAP-expressing cells, while the yield of S100B and GFAP co-expressing cells was 40% (Figures 1C and 1D). This is in stark contrast to previous conversion of neonatal fibroblasts whereby the yield was only 2% (Caiazzo et al., 2015). rtTA-infected controls never expressed GFAP and only very rarely low-expressing S100B cells were found (Figure 1C).

We observed that the majority of S100B^+^ and GFAP^+^ cells had elongated or polygonal fibroblast-like morphology (Figures 1C and 1E) resembling human primary astrocytes cultured with serum but normally not present *in vivo* (Zhang et al., 2016). We hypothesized that introducing serum-free medium adapted for astrocytes as well as supplementation with small molecules important for astrogliogenesis could improve conversion and morphology of converted cells. To address this we tested three different conditions: (1) serum-free medium alone (Serum-free); (2) serum-free medium with leukemia inhibitory factor, bone morphogenetic protein 4 (BMP4), ciliary neurotrophic factor (CNTF), and cyclic AMP (cAMP) (Serum-free + SM); and (3) an initially low concentration (0.2%) of serum-containing medium with fibroblast growth factor 2 (FGF2), together with BMP4 and CNTF between days 4 and 11 followed by serum-free medium with BMP4, CNTF, and cAMP (FGF2 + Serum-free + SM).

By gradually switching to Serum-free medium, many S100B^+^ and GFAP^+^ SAB-infected HEFs developed a star-like morphology with thick processes. However, there was still a substantial number of cells displaying elongated or polygonal morphology. Interestingly, in Serum-free + SM medium, S100B^+^ and GFAP^+^ cells displayed smaller somas and more thin, branched processes. Strikingly, FGF2 + Serum-free + SM medium resulted in a larger proportion of S100B^+^ and GFAP^+^ cells that developed morphologies similar to that of stellate primary astrocytes (Figure 1E). Furthermore, quantification revealed that this condition resulted in the highest yield of converted cells, with more than 80% S100B^+^, around 50% GFAP^+^, and 40% S100B^+^/GFAP^+^ cells (Figure 1F).

We obtained slightly higher purity of GFAP^+^ and S100B/GFAP co-expressing cells using Serum-free medium without SM (Figure 1F) but reasoned that the overall higher yield of cells expressing S100B and GFAP, and improved astrocytic morphology when using FGF2 + Serum-free + SM medium, was the best condition and was therefore used in subsequent experiments. Finally, by culturing SAB-infected HEFs for an additional 2 weeks some cells developed clear stellate astrocytic morphologies (Figure 1G). In addition, 31% expressed ATP1B2, described to be enriched in astrocytes (Zhang et al., 2016). However, expression was not specific to converted cells, as 15% of control cells also expressed ATP1B2 (Figures S1A and S1B).

Taken together, these findings indicate that selection of cells expressing high levels of transcription factors and optimization of media enables effective direct conversion of human fibroblasts to astrocytes, which we hereafter refer to as SAB-HEF-iAs.

### Human induced astrocytes are functional

**Figure 2:**
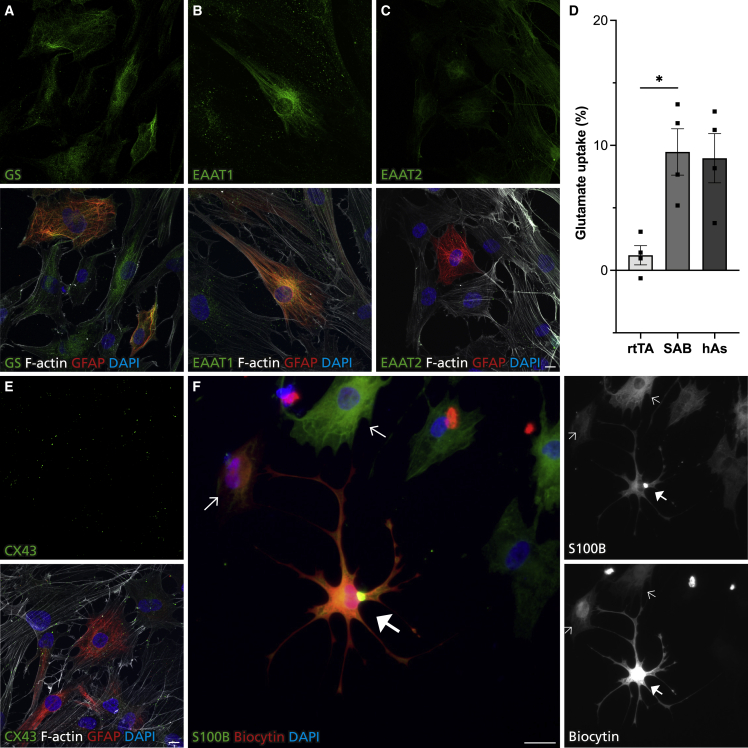
iAs express canonical astrocytic proteins and perform astrocytic functions (A–C) Representative maximum-intensity projection immunofluorescence images of GS and glutamate transporters in SAB-HEF-iAs. (D) Glutamate uptake of rtTA-infected control HEFs, SAB-HEF-iAs, and primary human fetal astrocytes (hAs). (E) Representative maximum-intensity projection immunofluorescence images of CX43 in SAB-HEF-iAs. (F) Example immunofluorescence image of SAB-HEF-iAs showing spread of biocytin between S100B^+^ cells. Arrows indicate cells with biocytin and thick full arrow the injected cell. Individual color levels are adjusted for visibility. Data are presented as mean ± SEM of n = 4 independent viral transduction experiments at 5 weeks. Kruskal-Wallis test was performed for statistical analysis in (D). ^∗^p < 0.05. Scale bars, 10 μm (A, B, C, and E) and 25 μm (F). See also Figure S1.

Astrocytes take up glutamate and convert to glutamine to prevent toxic accumulation and maintain synaptic transmission (Allen and Eroglu, 2017). To assess this crucial function in SAB-HEF-iAs, we analyzed the expression of glutamate transporters EAAT1, EAAT2, and glutamine synthetase (GS), responsible for glutamate-glutamine conversion (Verkhratsky and Nedergaard, 2018). We found that the vast majority of SAB-HEF-iAs expressed GS and were decorated with EAAT1 and EAAT2 puncta, although the latter was expressed at low levels (Figures 2A–2C). Next we assessed glutamate uptake. Importantly, SAB-HEF-iAs took up glutamate to similar extent as primary human fetal astrocytes and significantly more than rtTA-infected control HEFs (rtTA-HEFs) (Figure 2D).

Another important property of astrocytes is to form functional syncytia through gap-junction coupling, essential for buffering ions and metabolites to maintain brain homeostasis (Sofroniew and Vinters, 2010). To investigate this, we first analyzed the presence of the gap-junction protein CX43 and found that GFAP^+^ SAB-HEF-iAs expressed CX43^+^ puncta (Figure 2E). Next, we injected single cells with biocytin, a dye that diffuses through functional gap junctions. We observed that injected S100B^+^ SAB-HEF-iAs spread biocytin to neighboring S100B^+^ SAB-HEF-iAs (Figure 2F) as well as non-converted fibroblasts. In line with previous reports (Zhang and Cui, 2017), rtTA-HEFs expressed CX43, confirmed using two different antibodies as well as by qRT-PCR, and biocytin spread also occurred between these cells (Figures S1C–S1F).

Taken together, these findings indicate that SAB-HEF-iAs perform key astrocytic functions.

### Human induced astrocytes show transcriptional similarity to primary human astrocytes

**Figure 3:**
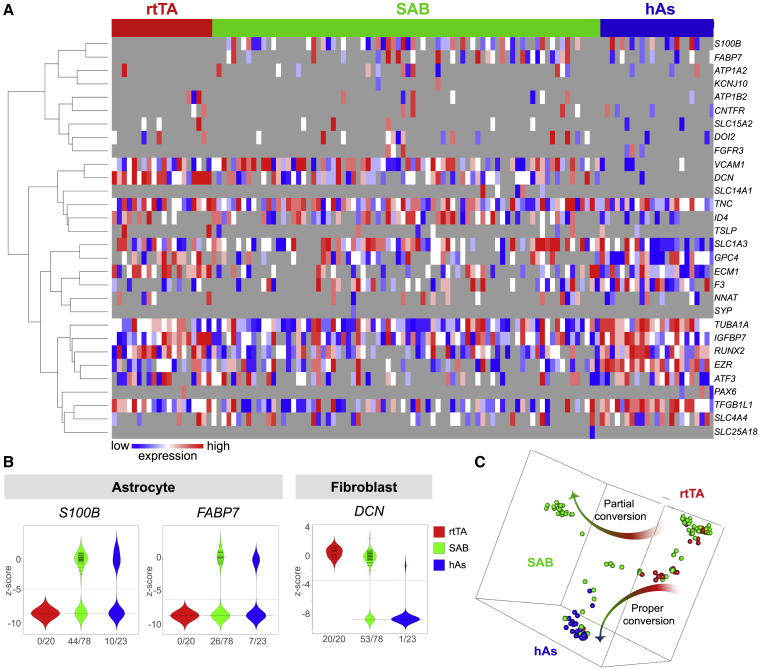
Single-cell gene expression analysis reveals heterogeneity and transcriptional similarities between iAs and human astrocytes (A) Hierarchical clustering based on expression of astrocyte, fibroblast, and neural lineage genes in rtTA-infected control HEFs (red), SAB-infected HEFs (green), and primary human fetal astrocytes (hAs) (blue). (B) Up- and downregulated genes during conversion. Data are presented as inverted Ct values *Z*-scored. (C) DDRTree clustering reveals two trajectories of SAB-infected HEFs emerging from rtTA-HEFs. Data were collected from n = 4 independent experiments. See also Table S4.

Despite efficient conversion, SAB transduced cultures likely consist of both iAs and non-converted cells. To investigate molecular phenotypes and heterogeneity of SAB-infected HEFs, we performed single-cell gene expression. We analyzed the expression of 30 genes, enriched in astrocytes, fibroblasts, or neural lineages, in 20 rtTA-infected HEFs, 78 SAB-infected HEFs, and 23 human primary fetal astrocytes. As expected, we found primary astrocytes to express a number of astrocytic genes not expressed in rtTA-HEFs, which on the other hand expressed the fibroblast-enriched gene *DCN* (Philippeos et al., 2018). This allowed us to identify several populations within SAB-infected HEFs. One expressed astrocyte genes (*S100B* and *FABP7*) but not *DCN*. Another expressed both astrocyte genes and *DCN*, and one population expressed only *DCN* (Figures 3A and 3B). Very few cells expressed neural progenitor (*PAX6*) or neuronal (*SYP*) genes (Figure 3A) and we never observed any immunoreactivity for βIII-TUBULIN (data not shown), excluding that conversion induced a neuronal fate. Furthermore, DDRTree clustering indicated that SAB-infected HEFs follow two main trajectories during conversion, both diverging from rtTA-HEFs and, importantly, one leading toward and clustering with primary human fetal astrocytes (Figure 3C). These findings confirm that SAB-infected HEFs is a heterogeneous population consisting of non-converted and partially converted cells but importantly containing one subpopulation that shows similarity to human fetal astrocytes.

### Omitting *Nfia* improves efficiency of conversion

**Figure 4:**
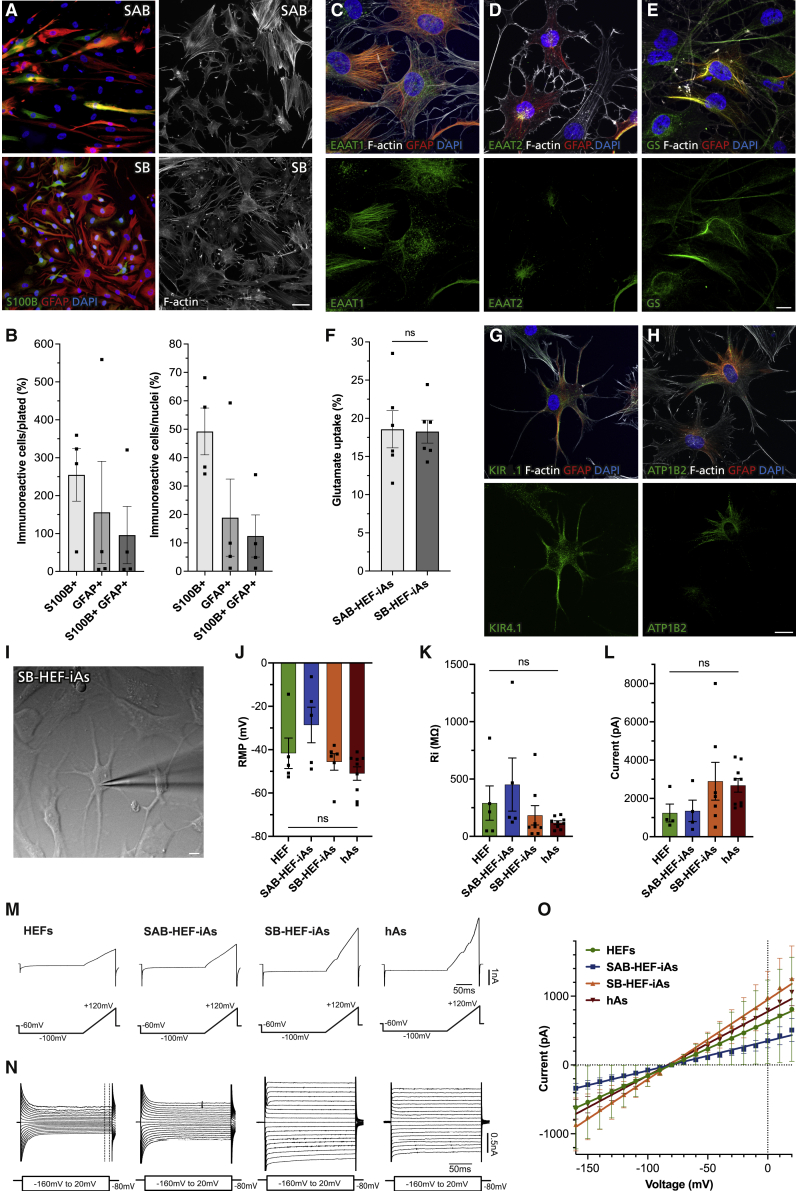
*Sox9* and *Nfib* improve conversion of human embryonic fibroblasts to induced astrocytes (A) Representative immunofluorescence images of HEFs 5 weeks after induction of *Sox9*, *Nfia*, and *Nfib* (SAB) or *Sox9* and *Nfib* (SB). (B) Quantification of yield and purity of S100B, GFAP, and S100B/GFAP immunoreactive cells at 3 weeks. (C–E) Representative maximum-intensity projection immunofluorescence images of glutamate transporters and GS in SB-HEF-iAs at 5 weeks. (F) Glutamate uptake in SAB-HEF-iAs and SB-HEF-iAs at 5 weeks. (G and H) Representative maximum-intensity projection immunofluorescence images of KIR4.1 and ATP1B2 in SB-HEF-iAs at 5 weeks. (I) Example image of whole-cell patch-clamp recording of SB-HEF-iAs with patch pipette indicating a recorded cell. (J and K) Resting membrane potential (RMP) and input resistance (Ri) values. (L) Values of maximum current induced by ramp depolarization protocol. (M) Current trace examples induced by ramp depolarization. (N) Examples of current traces induced by voltage steps ranging from −160 to +20 mV. Dotted lines indicate portion of the trace where steady-state currents were quantified. (O) Current/voltage (I/V) curve typical for astrocytes built from data obtained in (N). hAs, primary human fetal astrocytes. Individual color levels are adjusted for visibility in (A). Data are presented as mean ± SEM of n = 2–6 independent experiments. Data points in (J), (K), and (L) represent individual cells. Wilcoxon matched-pairs rank test (F), Kruskal-Wallis test (J, K, L), and multiple unpaired t tests (O) with significance level p < 0.05 were performed for statistical analysis. Scale bars, 50 μm (A) and 10 μm (C, D, E, G, H, and I). See also Figures S2, S4, and S5.

Our immunocytochemical and transcriptional analysis showed that conversion could be further optimized. Recently, several reports have pointed toward the importance of nuclear factor 1A (NFIA) in the gliogenic switch but not for astrocyte maturation (Tchieu et al., 2019; Tiwari et al., 2018). To investigate individual contribution of *Sox9*, *Nfia*, and *Nfib* and establish a minimal combination for optimal conversion, we performed a screen where HEFs were infected with single, pairs, or all three transcription factors (Figure S2A). Three weeks after induction, we analyzed the expression of GFAP and S100B by immunocytochemistry. In conditions with *Nfia* or *Nfib* alone we found S100B^+^ cells to be extremely rare. In contrast, *Sox9* was sufficient to induce S100B expression in some cells. However, we could not detect any GFAP-expressing cells using either *Sox9* or *Nfia*, whereas rare GFAP^+^ cells were found using *Nfib*. The *Nfia* + *Nfib* condition resulted in some S100B^+^ but few GFAP^+^ cells. On the other hand, with *Sox9* + *Nfia* or *Sox9* + *Nfib* we observed more cells expressing S100B and GFAP. Strikingly, *Sox9* + *Nfib*-infected HEFs generated substantially more S100B^+^ and GFAP^+^ cells as compared with all three transcription factors. In addition, a larger proportion of cells displayed astrocytic morphologies when using *Sox9* + *Nfib* (Figure S2A). This was even more striking after an additional 2 weeks when F-actin staining, used for better visualization of morphology, revealed an apparent improved astrocytic morphology compared with SAB-HEF-iAs (Figure 4A).

Quantification at 3 weeks confirmed our observation that the *Sox9* + *Nfib* (SB) condition improved conversion with a yield of 255%, 156%, and 96% and purity of 49%, 19%, and 12% S100B^+^, GFAP^+^, and S100B^+^/GFAP^+^ co-expressing cells, respectively (Figure 4B). This is approximately a 3-fold increase in yield compared with SAB-infected HEFs, while purity remained similar. The increased yield could be explained by better survival or increased proliferation. To assess this, we quantified total number and Ki67^+^ cells at 1 and 3 weeks. Total cell numbers were higher in SB compared with SAB conditions at 1 week. At 3 weeks, SAB cultures with serum had similar number of cells as SB, but higher than SAB in FGF2 + Serum-free + SM conditions (Figure S2B). Proliferation was overall low, especially at 3 weeks when less than 1% of S100B- and/or GFAP-expressing cells were Ki67^+^. We could not detect any significant differences between conditions, only a tendency toward higher proliferation in SAB-infected HEFs cultured with serum at 3 weeks (Figures S2C and S2D).

Taken together, these data indicate that SB-infected HEFs have higher survival, but proliferation most likely does not contribute to the increased yield.

Furthermore, using qRT-PCR we observed similar levels of *S100B* in SAB and SB but increased *GFAP* and further decreased expression of *DCN*, supporting downregulation of a fibroblast gene program and upregulation of astrocytic genes when using SB (Figure S2E).

To exclude that conversion induces skeletal muscle or renal epithelia, cells that also express GFAP, we analyzed the expression of MyoD and ANP by immunocytochemistry but never detected any immunoreactive cells (Figure S3).

Furthermore, AQP4 and ALDH1L1, reported as astrocyte-enriched markers (Verkhratsky and Nedergaard, 2018), were expressed in GFAP^+^ cells in both SAB- and SB-infected HEFs and surprisingly also in control fibroblasts (Figures S4A and S4B), though in line with previous studies (Khan et al., 2018; Mola et al., 2009).

Overall, these findings indicate that; single transcription factors are not sufficient, *Nfia* is not needed, and the optimal transcription factor combination for efficient conversion is *Sox9* and *Nfib*.

### *Sox9* and *Nfib* are sufficient to induce functional astrocytes

Having established *Sox9* + *Nfib*, hereafter referred to as SB-HEF-iAs, as the best combination, we next assessed functional properties by testing the ability to take up glutamate. Similar to SAB-HEF-iAs, the vast majority of SB-HEF-iAs expressed EAAT1 and EAAT2 as well as GS (Figures 4C–4E). In line with previous studies, EAAT1, EAAT2, and GS immunoreactivity was also observed in controls (Cooper et al., 1998; Soni et al., 1991) (Figures S4C–S4E). We compared levels of these markers in rtTA-HEFs as well as in GFAP^+^ SAB- and SB-HEF-iAs by measuring fluorescence intensity. We observed no difference between conditions in EAAT1, whereas EAAT2 intensity was higher in SB-HEF-iAs than in SAB-HEF-iAs and GS was higher in both SAB-HEF-iAs and SB-HEF-iAs as compared with rtTA-HEFs (Figure S4F). Importantly, SB-HEF-iAs had glutamate uptake similar to that of SAB-HEF-iAs, suggesting that they are equally functional (Figure 4F). Furthermore, SB-HEF-iAs also expressed CX43 and spread biocytin to neighboring S100B^+^ and GFAP^+^ iAs, suggesting formation of functional gap junctions (Figures S4G and S4H).

Functional syncytia are crucial for homeostatic functions of astrocytes, but expression of ion channels and transporters is also important for buffering. We observed that GFAP^+^ SAB-HEF-iAs and SB-HEF-iAs expressed higher levels of KIR4.1 than rtTA-HEFs, with SB-HEF-iAs displaying the highest levels (Figures 4G, S5A, and S5B). In addition, ATP1B2 was expressed in 34% of SB-infected cells, and 17% of cells co-expressed ATP1B2 and GFAP, compared with 3% in the SAB condition (Figures 4H, S5C, and S5D).

Expression of KIR4.1 and ATP1B2 suggested that HEF-iAs had machinery to buffer potassium and attain their most critical homeostatic function (Nwaobi et al., 2016). To assess this further, we performed electrophysiological recordings of SAB-HEF-iAs and SB-HEF-iAs at days 37–42 as well as of control HEFs and primary human fetal astrocytes (Figures 4I–4O). First we analyzed passive membrane properties, such as resting membrane potential (RMP) and input resistance (Ri). Interestingly, SB-HEF-iAs had an RMP of −46 mV and Ri of 184 MΩ, similar to human fetal astrocytes, which was −51 mV and 120 MΩ, respectively. This is in line with previous reports from *in vitro* stem cell-derived and fetal human astrocytes (Neyrinck et al., 2021). On the other hand, SAB-HEF-iAs had lower hyperpolarized RMP of −28 mV and higher Ri of 452 MΩ, and HEFs an RMP of −42 mV and Ri of 291 MΩ (Figures 4J and 4K).

Next, we analyzed whole-cell currents, which in astrocytes predominantly reflect the presence of K^+^ channels. We executed two protocols in voltage clamp mode. First, a ramp of depolarizing currents was evoked and quantified at the peak where voltage reached +120 mV (Figures 4L and 4M). In all groups, large outward-rectifying currents at potentials higher than −40 mV were observed. Induced currents were bigger, although not significantly so, in SB astrocytes and human primary astrocytes compared with SAB-HEF-iAs and HEFs, respectively. This observation agrees with previous data that primary cultured astroglial cells express large outward-rectifying K^+^ currents (Bevan and Raff, 1985).

Second, we induced whole-cell currents using a voltage-step protocol ranging from −160 mV to +20 mV at holding potential of −80 mV and used the steady-state current to plot a quasi-linear current/voltage (I/V) curve, characteristic for astrocytes (Figures 4N and 4O). Mature astrocytes have more voltage-gated K^+^ conductance, leading to membrane hyperpolarization, general high cell conductance, and low membrane resistance, reflected in the linear I/V curve with a steeper slope (Dallérac et al., 2013). Interestingly, SB-HEF-iAs had characteristics closest matching to human astrocytes, although data between groups were variable.

These findings show that both SAB-HEF-iAs and SB-HEF-iAs have electrophysiological features characteristic for astrocytes and indicate that SB-HEF-iAs performs slightly better.

In summary, by removing *Nfia*, significantly more iAs are generated and a larger proportion displays morphologies closer to bona fide astrocytes. Furthermore, SB-HEF-iAs have similar or better astrocyte marker expression and function compared with SAB-HEF-iAs.

### SB-HEF-iAs display ATP-induced calcium signaling and are immunocompetent

**Figure 5:**
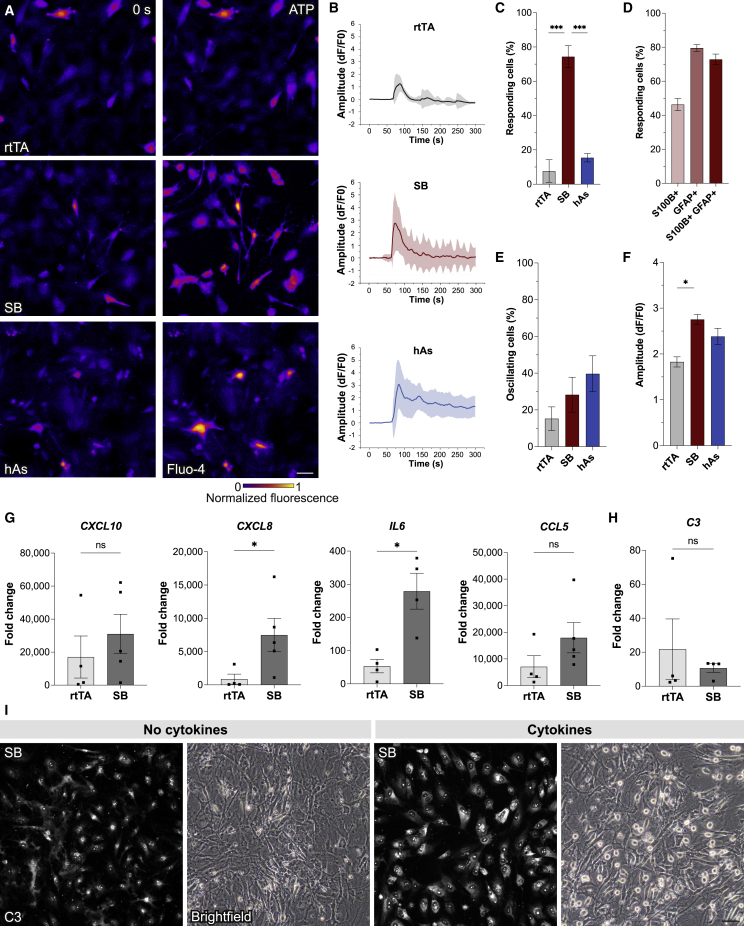
SB-HEF-iAs display ATP-induced calcium response and are immunocompetent (A) Background subtracted images of live-cell calcium imaging experiments on Fluo-4-loaded rtTA-HEFs and SB-HEF-iAs at 5 weeks, and primary human fetal astrocytes (hAs). (B) Mean relative fluorescence changes of calcium traces of ATP-responding cells normalized to baseline (dF/F0). (C) Percentage of ATP-responding cells using a threshold of 3 × SD of baseline. (D) Percentage of ATP-responding cells of S100B, GFAP, and S100B/GFAP co-expressing SB-HEF-iAs. (E) Percentage of oscillating cells of ATP-responding cells. (F) Amplitude of ATP-evoked response in responding cells. (G and H) qRT-PCR analysis upon stimulation with TNFα, IL-1α, and C1q. Data are presented as fold change relative to non-stimulated controls. (I) Representative immunostaining and bright-field image of SB-HEF-iAs following treatment with TNFα, IL-1α, and C1q. Data are presented as mean ± SEM, except for calcium traces (B) presented as mean ± SD, of n = 3–4 independent experiments. Kruskal-Wallis test (C–F) and Mann-Whitney test (G and H) were performed for statistical analysis. ^∗^p < 0.05, ^∗∗∗^p < 0.001. Scale bars, 50 μm (A and I). See also Figure S6 and Video S1. Calcium imaging of rtTA-HEFs, Video S2. Calcium imaging of SB-HEF-iAs, Video S3. Calcium imaging of primary human fetal astrocytes.

Astrocytes are known to respond to stimuli by regulating intracellular calcium levels. To assess this, we performed calcium imaging experiments with Fluo4-loaded SB-HEF-iAs at days 37–38. We observed a clear response to ATP in SB-HEF-iAs with 74% of cells having ATP-induced calcium waves that was distinct from rtTA-HEFs and even human fetal astrocytes, in which only 7.6% and 15% of cells responded, respectively (Figures 5A–5C; Videos S1, S2, and S3). Importantly, post hoc identification revealed that 46%, 80%, and 73% of S100B^+^, GFAP^+^, and S100B^+^/GFAP^+^ co-expressing SB-HEF-iAs, respectively, responded to ATP, indicating that the majority of GFAP^+^ cells display ATP-induced calcium waves (Figure 5D). Of the responding cells, slightly more SB-HEF-iAs showed oscillations, though not significantly more than rtTA-HEFs (Figure 5E). However, the amplitude of evoked responses was higher in SB-HEF-iAs (Figure 5F). This suggests that SB-HEF-iAs acquired intercellular calcium signaling similar to and even more prominent than primary fetal astrocytes that is distinct from control HEFs.

Video S1. Calcium imaging of rtTA-HEFs

Video S2. Calcium imaging of SB-HEF-iAs

Video S3. Calcium imaging of primary human fetal astrocytes

Next, we assessed immunological functionality, since astrocytes participate in regulation of CNS inflammation (Sofroniew and Vinters, 2010). We stimulated SB-HEF-iAs for 24 h with C1q, tumor necrosis factor α (TNFα) and interleukin-1α (IL-1α), a cocktail shown to activate mouse and human pluripotent stem cell (hPSC)-derived astrocytes (Barbar et al., 2020; Liddelow et al., 2017), and analyzed gene and protein expression. Strikingly, SB-HEF-iAs upregulated cytokines *CXCL10*, *CXCL8*, *IL-6*, and *CCL5*, and immune-related complement factor *C3*. Upregulation of *CXCL8* and *IL-6* was significantly higher in SB-HEF-iAs as compared with rtTA-HEFs (Figure 5G). We also observed downregulation of *MKI67* and *GFAP* and upregulation of *VIM* and *CD44* in cytokine-treated SB-HEF-iAs (Figure S6A), in line with previous studies on hPSC-derived astrocytes (Barbar et al., 2020). In addition we observed that SB-HEF-iAs had higher levels of C3D protein accompanied by rounding of the soma (Figures 5H and 5I), similar to what was described for hPSC-derived astrocytes (Tchieu et al., 2019). These data suggest that SB-HEF-iAs are immunocompetent.

Taken together, SB-HEF-iAs acquired astrocytic functional properties that are distinct from control HEFs.

To assess the importance of reprogramming factor expression during conversion, we analyzed SOX9 using immunocytochemistry in SB-infected HEFs. We found no difference in the number of SOX9^+^ cells between converted and non-converted cells at 1 and 3 weeks. However, intensity of SOX9 staining was higher in converted than in non-converted cells at 1 week. Surprisingly, at 3 weeks there was slightly higher intensity in non-converted cells (Figures S6B and S6C), suggesting that initial high levels of transgenes are important although other factors such as repressive mechanisms might also control conversion.

Finally, in an attempt to infer subtype identity to iAs, we performed qRT-PCR for markers of different developing CNS regions in SB-HEF-iAs at 5 weeks as well as human cortical fetal astrocytes. SB-HEF-iAs expressed mainly markers for hindbrain (*MYBPC1* and *WIF1*) and spinal cord (*HOXB4*, *HOXC8*, and *HOXC6*) but also some forebrain markers (*OTX2*, *DMRT2A*, *CHRDL1*, and *CRYM*) (Figure S6D) while fetal cortical astrocytes expressed forebrain and ventral markers (*NKX2*.*1*). These results could indicate that SB-HEF-iAs have a more hindbrain/spinal cord identity compared with human cortical fetal astrocytes.

### Conversion of postnatal, adult, and aged human fibroblasts to astrocytes

**Figure 6:**
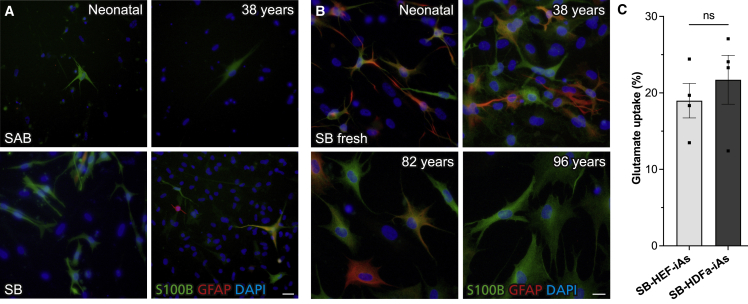
Direct conversion of human neonatal, adult, and aged fibroblasts to induced astrocytes (A and B) Immunofluorescence images of human neonatal and adult fibroblasts (38–96 years) 3 weeks after induction of SAB or SB (A) or freshly prepared SB (B). (C) Glutamate uptake of SB-infected HEFs and human adult fibroblasts (38 years) at 5 weeks. Individual color levels are adjusted for visibility in (A) and (B). Data are presented as mean ± SEM of n = 4 parallel independent experiments. Two-tailed Wilcoxon matched-pairs rank test with significance level p < 0.05 was performed for statistical analysis in (C). Scale bars, 50 μm (A) and 20 μm (B).

To enable modeling of neurological disorders using patient fibroblasts and utilizing advantages of direct conversion such as preservation of genetic heterogeneity and age- and disease-related features, successful conversion of postnatal human fibroblasts is essential. Therefore, we applied our SB protocol to neonatal and adult human fibroblasts. Indeed, 3 weeks after induction, we observed S100B^+^ and GFAP^+^ cells by immunostaining. Importantly, strengthening our previous findings, substantially more S100B^+^ and GFAP^+^ cells were observed by overexpressing SB as compared with SAB (Figure 6A).

Although we could obtain iAs from postnatal cells, conversion was not as efficient as in HEFs, which is in line with previous reports on direct conversion (Tian et al., 2016). We speculated that this could be due to adult fibroblasts requiring higher titer virus and increased transgene expression. We therefore tested freshly prepared, not previously frozen, viral preparations. Strikingly, this improved the conversion of neonatal as well as adult fibroblasts and allowed for conversion of aged fibroblasts (Figure 6B). In line with previous reports (Gatto et al., 2020), we observed that iAs obtained from adults were larger compared to neonatal iAs. To assess the functionality of iAs obtained from adult fibroblasts, we analyzed glutamate uptake and found that adult iAs took up glutamate at levels similar to those of as HEF-iAs (Figure 6C).

These findings indicate that removal of *Nfia* improved direct conversion of neonatal and adult fibroblasts to iAs. Importantly, this also allowed for conversion of fibroblasts derived throughout the human lifespan.

### Induced astrocytes support the function of induced neurons in co-cultures derived from the same starting fibroblast population

Rodent astrocytes are frequently used to support PSC-derived as well as directly converted neurons in co-culture. However, given the vast difference between rodent and human astrocytes, it would be desirable to directly convert neurons and astrocytes from the same starting population. Such co-cultures would enable disease modeling and studies of human neuron-astrocyte interactions. To establish this, we generated induced neurons (iNs), through direct conversion of HEFs using *Ascl1* and *Ngn2* overexpression (Ladewig et al., 2012), and cultured them alone or together with SB-HEF-iAs.

**Figure 7:**
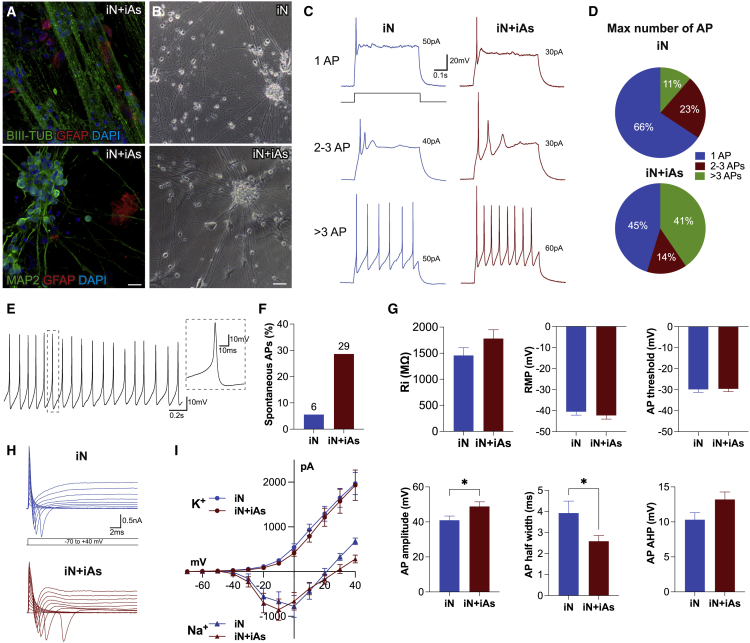
Improved functionality of induced neurons in co-cultures with iAs (A) Maximum-intensity projection immunofluorescence images of iNs in co-culture with SB-HEF-iAs (iN + iAs) at 6–8 weeks. (B) Bright-field images of iN alone and iN + iAs co-cultures. (C) Example traces of whole-cell current clamp recordings of iNs with three different patterns of action potential (AP) firing. APs are induced by depolarization current step at membrane potential of −70 mV. Current injected in each trace is indicated. (D) iNs grouped based on number of APs fired. (E) Example trace of recorded iNs with spontaneous APs at resting membrane potential (RMP). Inset shows magnified view of a single AP. (F) Percentage of iNs with spontaneous APs at RMP. (G) Intrinsic membrane properties and AP parameters of iNs. (H) Example traces of inward Na^+^ and outward K^+^ whole-cell currents evoked by voltage steps ranging from −70 to +40 mV in voltage clamp mode from holding potential of −70 mV. (I) Outward K^+^ and inward Na^+^ peak currents plotted against voltage steps shown in (H). Individual color levels are adjusted for visibility in (A). Data are presented as mean ± SEM of n = 38–40 cells from three independent experiments. Unpaired t test was performed for statistical analysis in (G). ^∗^p < 0.05. Scale bars, 20 μm (A) and 50 μm (B). See also Figure S7.

iNs expressed neuronal markers βIII-TUBULIN and MAP2 (Figure 7A), and we observed that when iNs were cultured alone, cells started to detach between days 33 and 47 in approximately 50% of coverslips. In contrast, when co-cultured with SB-HEF-iAs, cells detached only in around 4% of coverslips (Figures 7B and S7).

To test whether iAs influence the functional properties of neurons, we performed whole-cell patch-clamp recordings from iNs cultured alone or in co-culture with iAs at days 45–52 (Figures 7C–7I). We divided iNs by the ability to fire action potentials (APs). All recorded cells fired at least a single AP, but strikingly iNs in co-cultures had a higher proportion of cells able to repetitively and sustainably fire higher numbers (>3) of APs in response to increasing depolarizing current steps (41% in co-cultures versus 23% in iNs alone) (Figures 7C and 7D). The proportion of spontaneously active cells at RMP was also higher in the co-culture group (29% compared with 6%) (Figures 7E and 7F). We observed that AP characteristics such as amplitude and half-width were significantly different and closer to more mature neurons with higher AP amplitude when cultured with iAs (Figure 7G). Other intrinsic parameters (Ri and RMP) and AP characteristics (AP threshold and afterhyperpolarization) were not statistically different between groups (Figure 7G).

Another key property of functional neurons is the presence of active voltage-gated Na^+^ and K^+^ channels. Both fast inward Na^+^ and slow outward K^+^ currents could be induced by stepping voltage from −70 to +40 mV with 10 mV increments in voltage clamp configuration (Figure 7H); however, we did not observe any difference between groups in I/V curves obtained by quantification of these currents (Figure 7I). We did not observe any clear spontaneous postsynaptic currents in either iN alone or in co-cultures, indicating that a longer time is needed to reach full neuronal maturation and connectivity.

In conclusion, we present for the first time a co-culture system of functional human neurons and astrocytes obtained through direct conversion from the same starting fibroblast population, in which iAs support the maturation of iNs. This co-culture system has promising potential for disease modeling and the study of astrocyte-neuron interactions.

## Discussion

Here we describe effective generation of iAs from human fibroblasts, derived throughout the lifespan, using transcription factor-mediated direct conversion. iAs resembled primary fetal human astrocytes at a molecular, phenotypical, and functional level. We also, for the first time, describe a functional co-culture system with iAs and iNs obtained through direct conversion from the same starting human fibroblast population.

There are several methods to generate astrocytes from human embryonic stem cells (hESCs) and induced PSCs (iPSCs) (Canals et al., 2018; Mertens et al., 2016). However, methods to directly convert fibroblasts to astrocytes, such as the one described here, have only been briefly explored but have important advantages. Besides being faster, direct conversion preserves genetic mosaicism present in starting fibroblast populations, whereas iPSCs are clonal (Abyzov et al., 2012; Mertens et al., 2016). If the level of mosaicism is similar in astrocytes and fibroblasts, which is likely, directly converted cells would be better at recapitulating genetic heterogeneity. Furthermore, age-related epigenetic properties are erased during iPSC reprogramming and yield rejuvenated cells. Direct conversion studies on iNs and induced oligodendrocytes, on the other hand, have been shown to generate cells that retain aging phenotypes (Chanoumidou et al., 2021; Mertens et al., 2015). Thus, for modeling of sporadic neurodegenerative disorders, in which age is the most significant risk factor, direct conversion constitutes an attractive approach for generation of diseased cells.

The potency of *Sox9*, *Nfia*, and *Nfib* inducing astrocytic cell fate is in accordance with studies performed in developing embryonic chick spinal cord, showing that these transcription factors control the initiation of gliogenesis (Molofsky et al., 2012). However, we discovered that the yield of converted cells was higher when *Sox9* and *Nfib* (SB) were overexpressed. Supporting this finding, it was recently shown that transient overexpression of *NFIA* in hESC-derived neural stem cells yields more astrocytes compared with continuous overexpression, indicating that *NFIA* is important for initial specification of astrocytes but needs to be downregulated for maturation (Tchieu et al., 2019). This deviates from what was reported for direct conversion of mouse fibroblasts whereby SAB was more efficient than SB (Caiazzo et al., 2015). However, it is in line with our recent finding that SB efficiently drives astrocytic induction in hPSCs (Canals et al., 2018). We cannot exclude that sequential expression of first *Nfia* and then *Sox9* and *Nfib* would yield results similar to those described here but would require a more complex expression system.

Our approach improved the yield of directly converted astrocytes by over 40- and 120-fold using SAB or SB, respectively, compared with a previous study (Caiazzo et al., 2015). The starting fibroblast population could in part explain the increased efficiency. We opted to use embryonic fibroblasts to optimize conversion while Caiazzo et al. used neonatal fibroblasts. Indeed, conversion of postnatal fibroblasts was shown to be less efficient, possibly due to reduced cell plasticity, proliferation, and onset of senescence in older fibroblasts (Ahlenius et al., 2016). However, we also successfully converted adult and aging fibroblasts. Therefore, another plausible explanation is that by using selectable vectors we obtain a higher number of transduced cells and increased level of transgene expression, which is supported by our finding that freshly prepared viruses was crucial to conversion of postnatal fibroblasts. However, we also observed unconverted cells expressing at least one transgene, indicating that other mechanisms, such as repressive factors, might be involved in controlling conversion, as reported for neuronal conversion (Drouin-Ouellet et al., 2017).

Several lines of evidence, including morphological observations, immunocytochemical analysis, and gene expression profiling, support our interpretation that iAs are of astrocytic lineage. Importantly, we extensively characterized known functional properties of astrocytes in iAs and demonstrated that they take up glutamate, form functional gap junctions, are immunocompetent, display regulated calcium signaling, have appropriate electrophysiological properties, and support neuronal functionality (Sofroniew and Vinters, 2010). Interestingly, iAs expressed several markers reported in mature astrocytes (Lattke et al., 2021). However, since comparison with bona fide adult human astrocytes *in vivo* is not possible, it is difficult to judge exactly how mature iAs are, although we conclude that they are at least as mature as primary human fetal astrocytes.

Our finding that functional astrocytes can be derived directly from human fibroblasts is supported by a previous study that used chemical reprogramming to generate iAs from human foreskin fibroblasts (Tian et al., 2016). Chemical iAs were obtained at similar purity, could take up glutamate, and displayed induced calcium response. However, our method requires less time to generate astrocytes with additional functional properties such as gap-junction coupling, electrophysiological properties, and immunocompetence, and, importantly, iAs derived from all ages display morphology more closely resembling that of bona fide human astrocytes. In addition, the majority of established direct conversion methods are based on transcription factors, which makes it relatively easy to produce a variety of neural and non-neural cells without changing methodology and, as we show here, combine into useful co-cultures.

Furthermore, single-cell gene expression analysis of SAB-HEF-iAs allowed us to assess heterogeneity during conversion and revealed that the process yields a complex mixture of cells, which is not ideal for certain purposes. However, we identified three genes, *S100B*, *FABP7*, and *DCN*, which clearly separated control fibroblasts from astrocytes. Based on these genes, we identified a population that clustered closer to human primary fetal astrocytes than other populations.

SB-HEF-iAs expressed mainly hindbrain and spinal cord markers but also forebrain markers. These findings might suggest a more posterior identity, but perhaps a more plausible explanation is that iAs represent a generic astrocyte type.

Finally, we showed that iAs can be co-cultured with iNs obtained through direct conversion from the same starting fibroblasts and that iAs supported functionality of iNs. Similar co-culture systems based on hPSCs have been used to identify cell-autonomous and non-autonomous disease mechanisms and study astrocyte-neuron interactions, for instance in amyotrophic lateral sclerosis (Birger et al., 2019).

In conclusion, we have shown that overexpression of *Sox9* and *Nfib* in human fibroblasts effectively generates iAs with molecular and functional phenotypes similar to primary human astrocytes. Fibroblast-derived iAs, and in combination with iNs ultimately from patients with neurological diseases, have potential to become informative models to elucidate the role of astrocytes in disease, especially for studies on sporadic and age-related neurological disorders.

## Experimental procedures

### Human tissue

Human tissue was obtained from dead aborted human fetuses 6–9 weeks post conception according to guidelines approved by the Lund-Malmö Ethical Committee. Human dermal neonatal (C-004-5C) and adult (C-013-5C) fibroblasts were bought from Invitrogen. Aged human fibroblast from an 82-year-old woman (GM01706) and a 96-year-old man (GM00731) were obtained from Coriell biorepository.

### Direct conversion

Fibroblasts were seeded on day −2 and transduced on day −1 with rtTA, tetO-Sox9-puromycin, tetO-Nfib-hygromycin, and tetO-Nfia-blasticidin, either alone or in combinations, in Fibroblast medium (Dulbecco’s modified Eagle’s medium [DMEM], 10% fetal bovine serum [FBS], 1% GlutaMAX, 1% non-essential amino acid [NEAA], and 1% sodium pyruvate, all from Gibco) containing 8 μg/mL Polybrene (Sigma). Transgene expression was induced by addition of 2.5 μg/mL doxycycline (Sigma-Aldrich) at day 0 and kept throughout experiments. On day 1, a 3-day puromycin (Gibco) and a 5-day blasticidin (Gibco) and hygromycin (Gibco) selection was started during which medium was changed daily. For FGF2 + Serum-free + SM condition, medium was changed on day 3 to 3:4 Fibroblast medium/FGF medium (Neurobasal, B-27, 1% NEAA, 1% GlutaMAX, 0.2% FBS, 8 ng/mL FGF [Peprotech], 5 ng/mL CNTF [Peprotech], and 10 ng/mL BMP4 [Peprotech]). At day 4 the medium was changed to 1:1 Fibroblast medium/FGF medium and at day 6 1:4 Fibroblast medium/FGF medium. At day 7 the medium was changed to FGF medium and at day 9 half of the medium was changed. At day 11, half of the medium was changed to Serum-free medium (1:1 DMEM-F12, HEPES:Neurobasal, 1% N-2, 1% GlutaMAX, 1% sodium pyruvate, 5 μg/mL *N*-acetyl-L-cysteine [Sigma-Aldrich], and 5 μg/mL heparin-binding EGF-like growth factor [Sigma-Aldrich]) supplemented with 10 ng/mL CNTF, 10 ng/μL BMP4, and 500 μg/mL dibutyryl cAMP (Sigma-Aldrich). Thereafter, half of the medium was changed every second or third day. If needed, cells were split 1:3.

### Statistical analysis

Data are presented as mean ± SEM of three to six independent viral transduction experiments performed on separate days (n) unless otherwise stated.

See supplemental experimental procedures for further details.

## Author contributions

E.Q. was responsible for conceptualization, methodology, validation, investigation, visualization, formal analysis, funding acquisition, and preparation of the manuscript. F.T. performed calcium imaging and data analysis, and assisted in preparation of the manuscript. N.A., A.G.R., and M.G.H. performed electrophysiological recordings and data analysis, and assisted in preparation of the manuscript. O.G.Z. performed data analysis and assisted in preparation of the manuscript. M.K. supervised A.G.R. I.C. was responsible for methodology and supervision. H.A. was responsible for conceptualization, methodology, supervision, project administration, manuscript writing, and funding acquisition.

## Conflict of interests

E.Q. is now an employee at AstraZeneca. E.Q. and H.A. have filed a patent application based on findings described in this study.

## Supplemental information

Document S1. Figures S1–S6, Tables S1–S4, and supplemental experimental procedures

Document S2. Article plus supplemental information
