# Supplementary material for: Transcription factor-based direct conversion of human fibroblasts to functional astrocytes
Source: Stem Cell Reports. 2022 Jun 23;17(7):1620–35. doi: 10.1016/j.stemcr.2022.05.015 (PMC9287681; doi:10.1016/j.stemcr.2022.05.015)
Supplement: Document S1. Figures S1–S6, Tables S1–S4, and supplemental experimental procedures [file mmc1.pdf]

Stem Cell Reports, Volume *17*

Supplemental Information

Transcription factor-based direct conversion of human fibroblasts to functional astrocytes

Ella Quist, Francesco Trovato, Natalia Avaliani, Oskar G. Zetterdahl, Ana Gonzalez-Ramos, Marita G. Hansen, Merab Kokaia, Isaac Canals, and Henrik Ahlenius

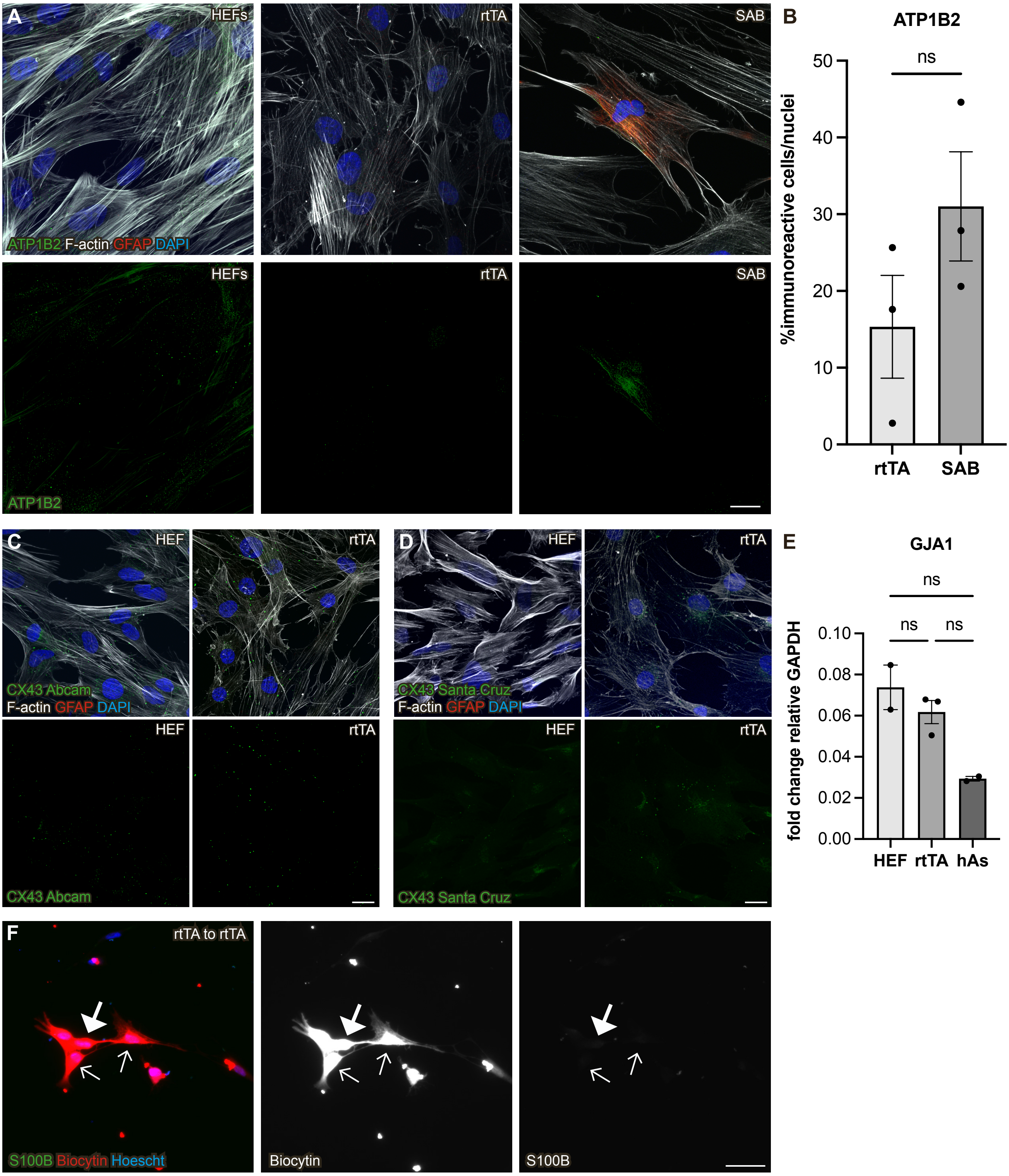
A
HEFs
rtTA
SAB
ATP1B2 F-actin GFAP DAPI
HEFs
rtTA
SAB
ATP1B2
B
ATP1B2
ns
%immunoreactive cells/nuclei
50
40
30
20
10
0
rtTA
SAB
C
HEF
rtTA
CX43 Abcam
F-actin GFAP DAPI
HEF
rtTA
CX43 Abcam
D
HEF
rtTA
CX43 Santa Cruz
F-actin GFAP DAPI
HEF
rtTA
CX43 Santa Cruz
E
GJA1
ns
ns
ns
fold change relative GAPDH
0.10
0.08
0.06
0.04
0.02
0.00
HEF
rtTA
hAs
F
rtTA to rtTA
S100B Biocytin Hoescht
Biocytin
S100B

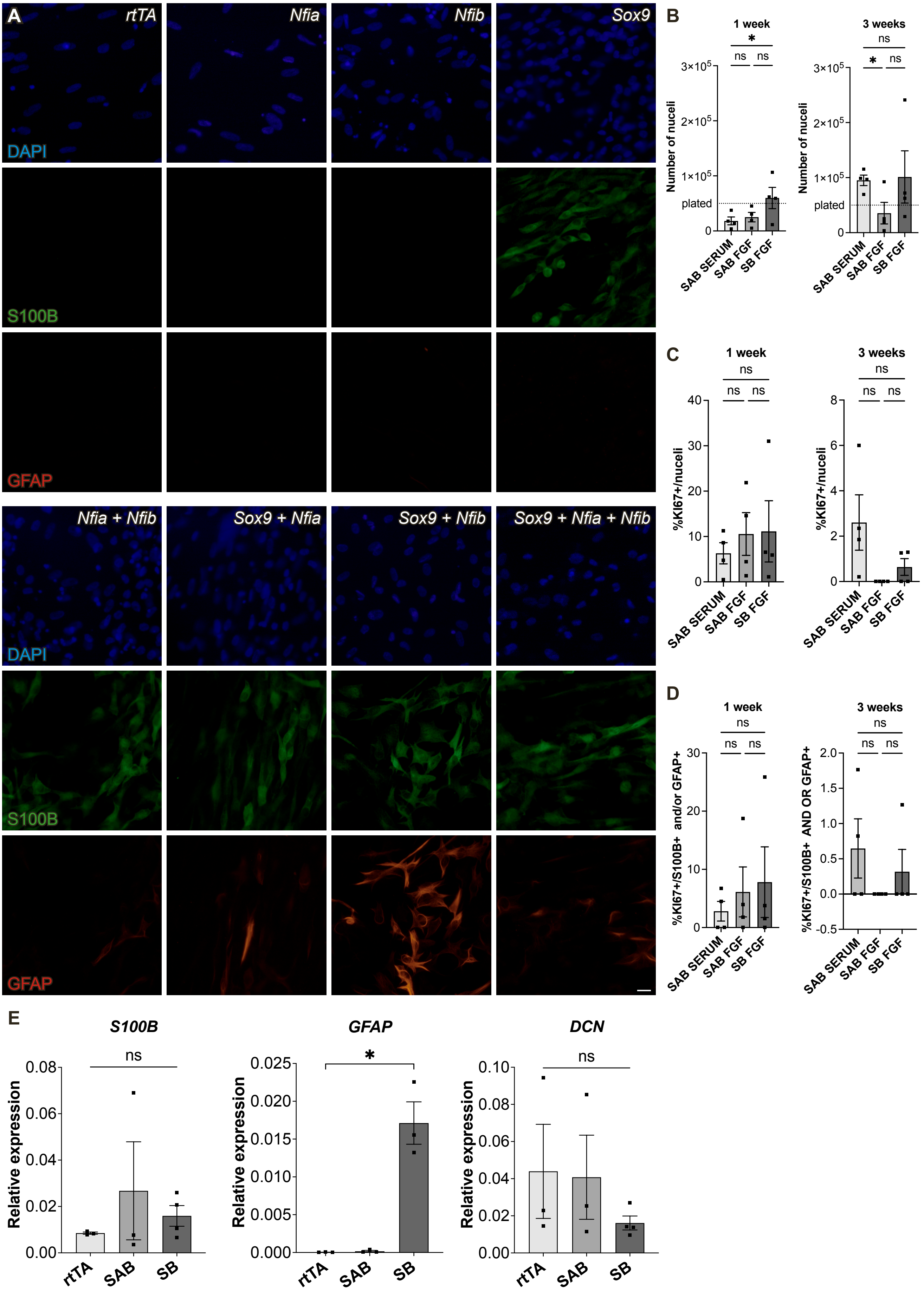

A
rtTA
Nfia
Nfib
Sox9
DAPI
S100B
GFAP
Nfia + Nfib
Sox9 + Nfia
Sox9 + Nfib
Sox9 + Nfia + Nfib
DAPI
S100B
GFAP
B
1 week
*
ns
ns
Number of nuceli
3×10^5
2×10^5
1×10^5
plated
0
SAB SERUM
SAB FGF
SB FGF
3 weeks
ns
*
ns
C
1 week
ns
ns
ns
%KI67+/nuceli
40
30
20
10
0
3 weeks
8
6
4
2
D
%KI67+/S100B+ and/or GFAP+
%KI67+/S100B+ AND OR GFAP+
2.0
1.5
1.0
0.5
0.0
-0.5
E
S100B
GFAP
DCN
Relative expression
0.08
0.06
0.04
0.02
0.00
0.025
0.020
0.015
0.010
0.005
0.000
0.10
rtTA
SAB
SB

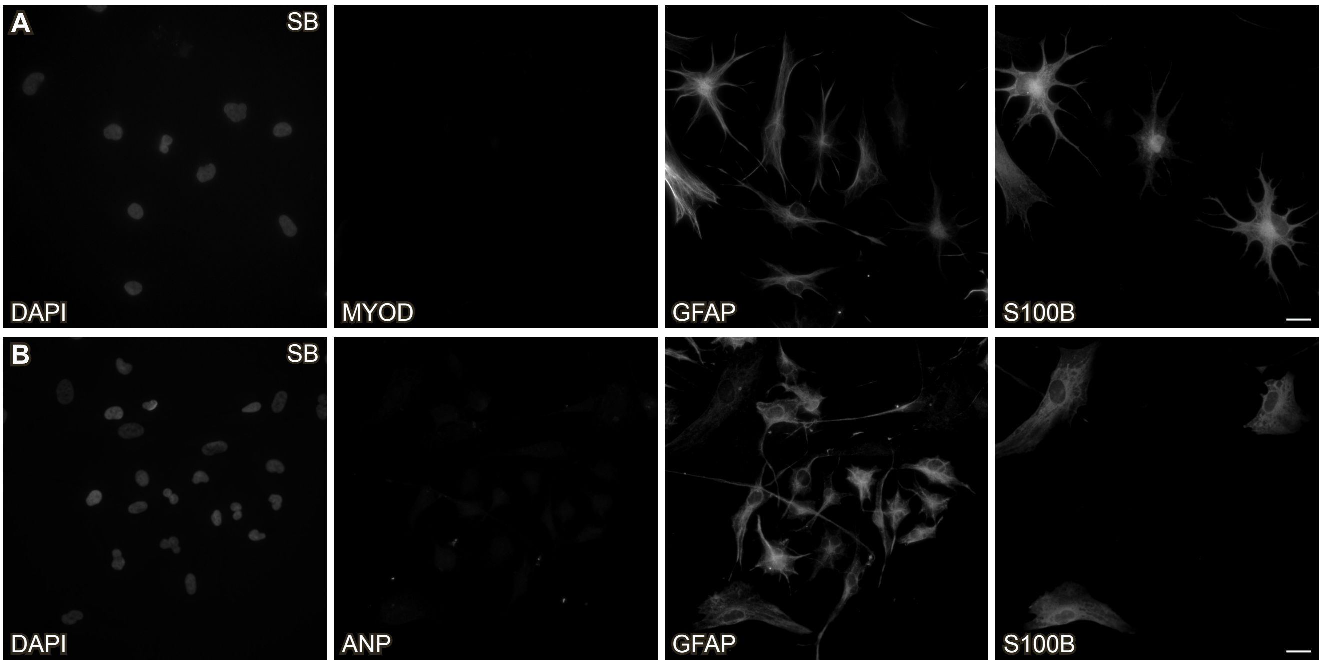
A
SB
DAPI
MYOD
GFAP
S100B
B
SB
DAPI
ANP
GFAP
S100B

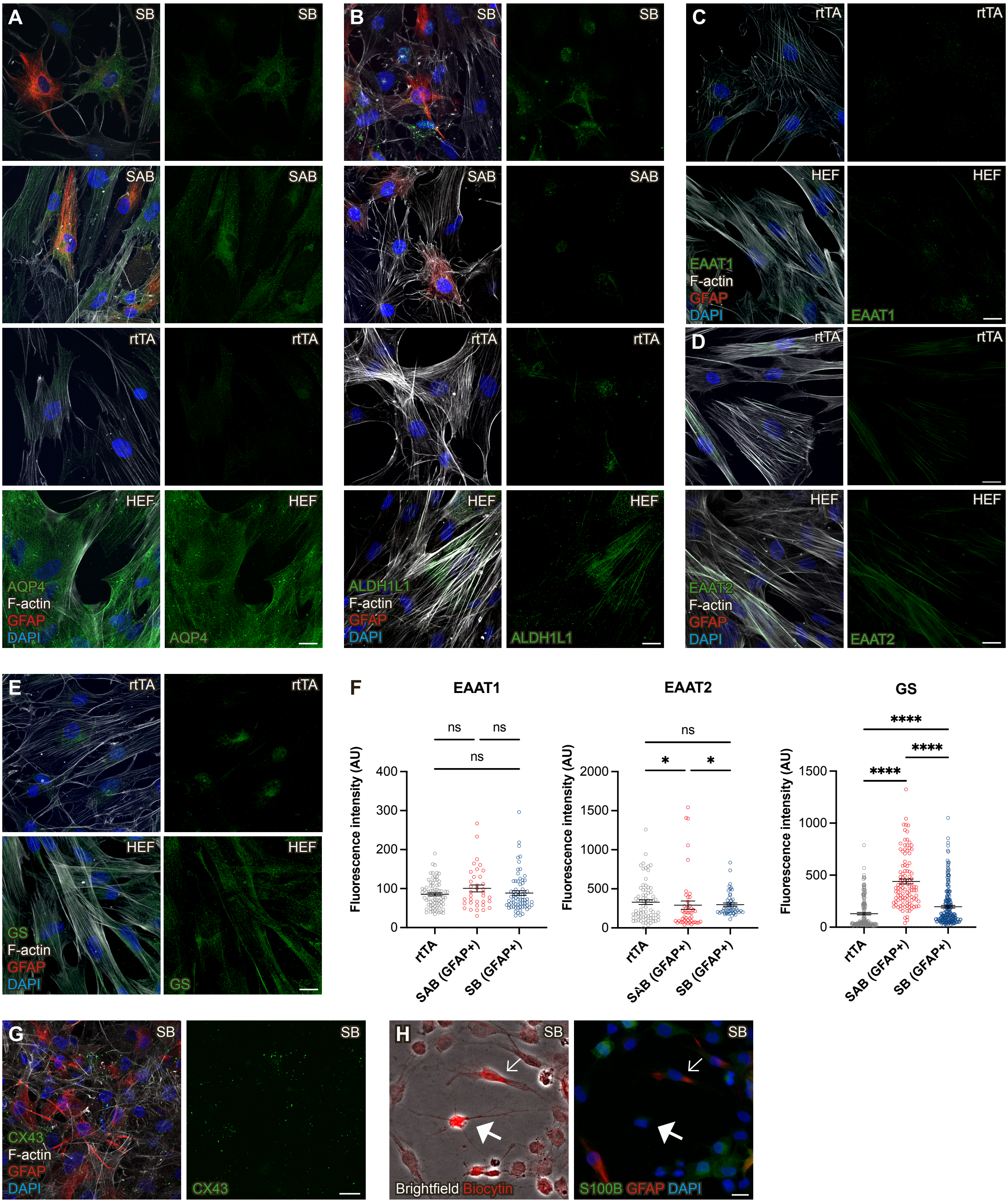
A
B
C
D
E
F
G
H
SB
SAB
rtTA
HEF
AQP4
F-actin
GFAP
DAPI
ALDH1L1
EAAT1
EAAT2
GS
EAAT1
EAAT2
GS
Fluorescence intensity (AU)
ns
*
****
rtTA
SAB (GFAP+)
SB (GFAP+)
0
100
200
300
400
500
1000
1500
2000
CX43
Brightfield Biocytin
S100B GFAP DAPI

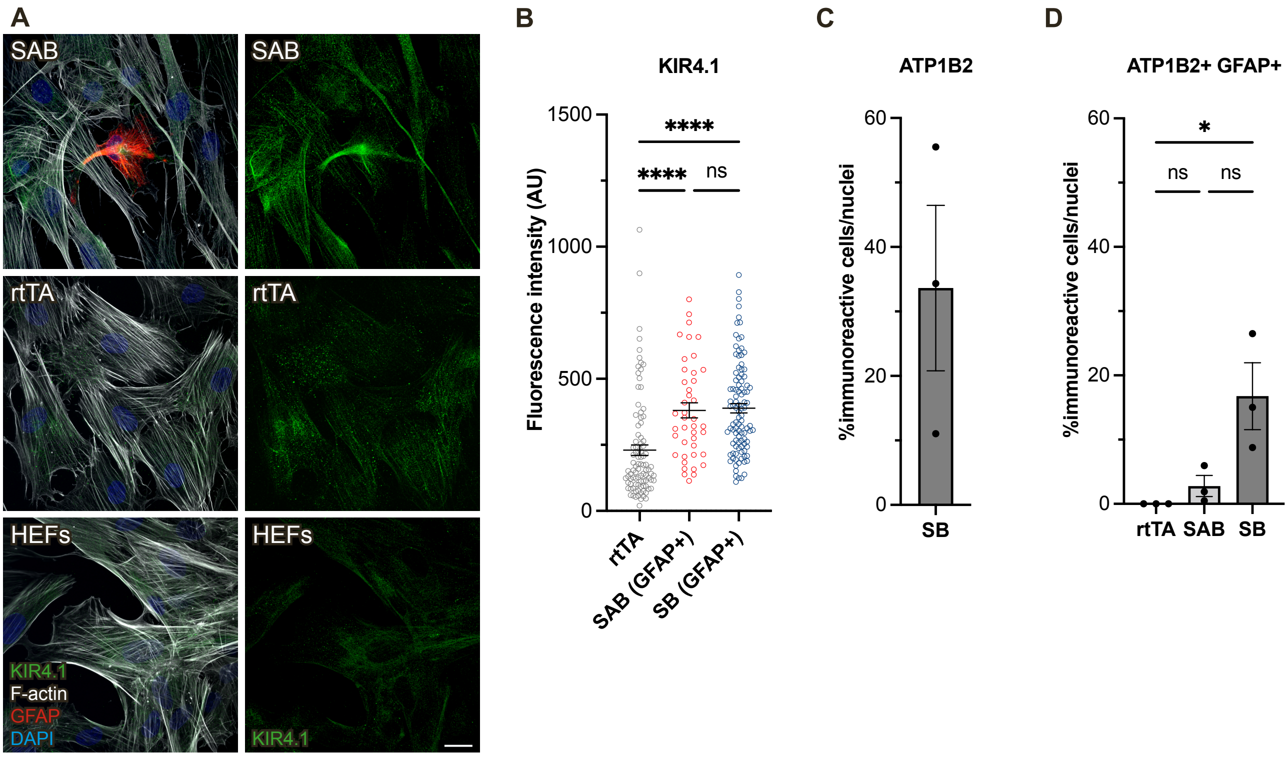
A
SAB
SAB
rtTA
rtTA
HEFs
HEFs
KIR4.1
F-actin
GFAP
DAPI
KIR4.1
B
KIR4.1
Fluorescence intensity (AU)
1500
1000
500
0
****
****
ns
rtTA
SAB (GFAP+)
SB (GFAP+)
C
ATP1B2
%immunoreactive cells/nuclei
60
40
20
0
SB
D
ATP1B2+ GFAP+
%immunoreactive cells/nuclei
60
40
20
0
*
ns
ns
rtTA
SAB
SB

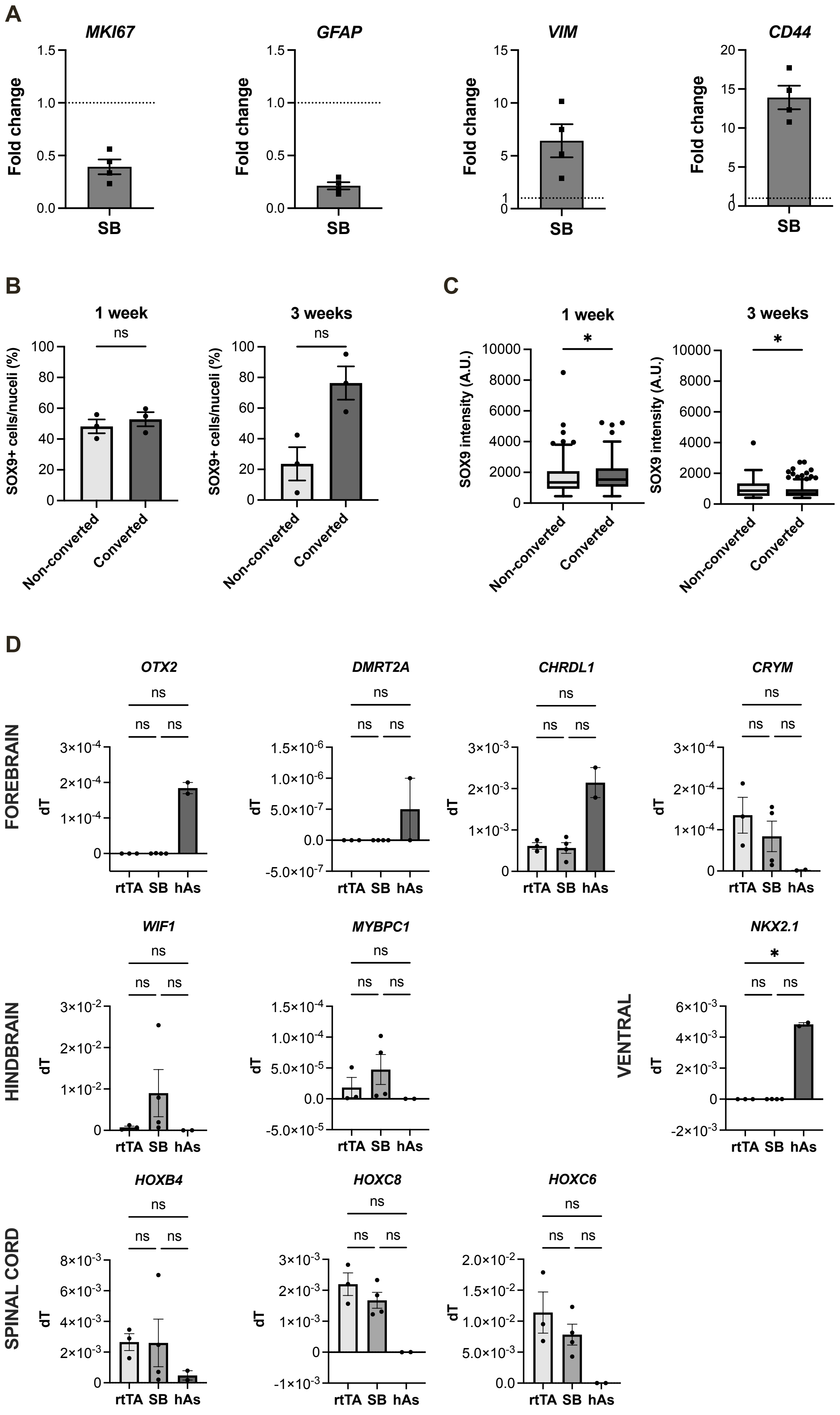
A
MKI67
GFAP
VIM
CD44
Fold change
SB
B
1 week
3 weeks
ns
SOX9+ cells/nuceli (%)
Non-converted
Converted
C
SOX9 intensity (A.U.)
*
D
FOREBRAIN
OTX2
DMRT2A
CHRDL1
CRYM
HINDBRAIN
WIF1
MYBPC1
VENTRAL
NKX2.1
SPINAL CORD
HOXB4
HOXC8
HOXC6
dT
rtTA
hAs

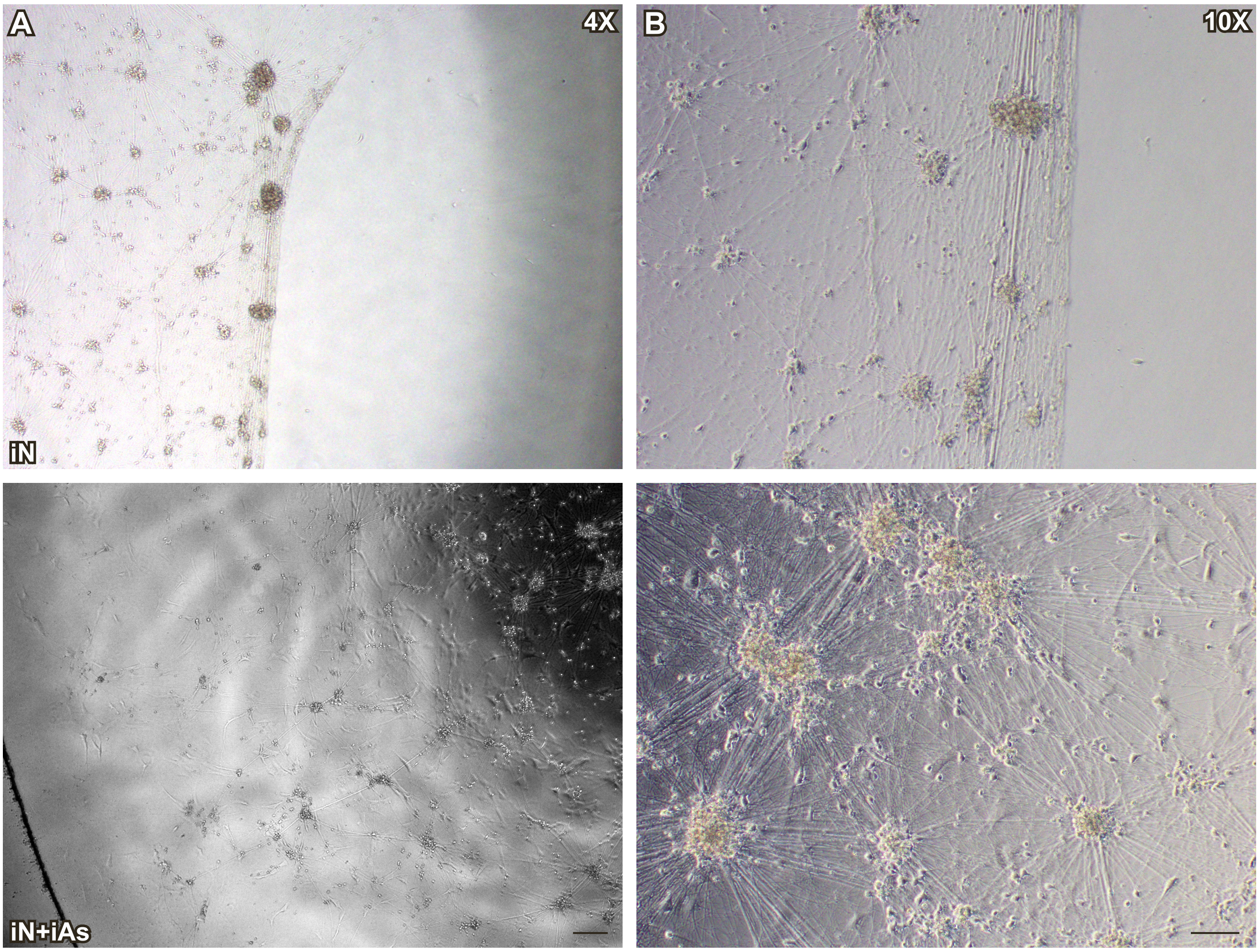
A
4X
iN
B
10X
iN+iAs

Supplemental Information

SUPPLEMENTAL FIGURES

Figure S1. Immunocytochemical analysis and biocytin spread in control HEFs. Related to Figure 2.
(A) Representative maximum intensity projection immunofluorescence images of ATP1B2 co-stained with GFAP and F-actin in rtTA-infected control HEFs (rtTA) and SAB-infected HEFs (SAB) at 5 weeks and untreated HEFs. **(B)** Quantification of ATP1B2 immunoreactive cells at 5 weeks related to total number of nuclei. **(C-D)** Representative maximum intensity projection immunofluorescence images of CX43 using two different antibodies in untreated HEFs and rtTA-infected control HEF at 5 weeks. (**E**) Gene expression of *GJA1* as determined by RT-qPCR shown relative to *GAPDH* in untreated HEFs, rtTA-infected control HEFs at 5 weeks and human fetal cortical astrocytes (hAs) (**F)** Example immunofluorescence image of biocytin injected rtTA-infected control HEFs. Arrowheads indicate cells with biocytin and thick full arrowhead biocytin-injected cell. Scale bar: 20 µm (A, C-D), 50 µm (F). Data are presented as mean±SEM of n=2-3 independent viral transduction experiments or RNA isolations done on separate days. Mann-Whitney (B) and Kruskal-Wallis (E) tests with significance level $p<0.05$ were performed for statistical analysis.

Figure S2. Transcription factor screen using *Nfia, Nfib* and *Sox9*. Related to Figure 4. (A) Representative immunofluorescence images of GFAP and S100B, 3 weeks after induction of *Nfia*, *Nfib* and *Sox9* either alone, in pairs or all three in combination. **(B)** Quantification of total number of nuclei in SAB-infected HEFs in serum containing media (SAB SERUM), SAB-infected HEFs in FGF2+Serum-free+SM media (SAB FGF) and SB-infected HEFs in FGF2+Serum-free+SM media (SB FGF) at 1 and 3 weeks. **(C)** KI67 expression related to number of nuclei. **(D)** KI67 expression in S100B and/or GFAP immunoreactive cells (converted cells). **(E)** Gene expression of *S100B*, *GFAP* and *DCN* as determined by RT-qPCR shown relative to *GAPDH* in rtTA-infected control HEFs and SAB- or SB-infected HEFs at 5 weeks. Scale bar: 20 µm (A). Data are presented as mean±SEM of n=3-4 independent viral transduction experiments. Friedman test (B-C) and Kruskal-Wallis (E) tests were performed for statistical analysis. *$p<0.05$.

Figure S3. SB-HEF-iAs conversion does not induce muscle or renal epithelia cells.
(A-B) Representative immunofluorescence images of MYOD and ANP co-stained with GFAP and S100B in SB-HEF-iAs at 5 weeks. Scale bar: 20 µm.

Figure S4. Additional characterization of SB-HEF-iAs. Related to Figure 4.
(A-B) Representative maximum intensity projection immunofluorescence images of AQP4 and ALDH1L1 co-stained with GFAP and F-actin of rtTA-infected control HEFs (rtTA), SAB-infected HEFs (SAB), SB-infected HEFs (SB), all at 5 weeks, and untreated HEFs. (**C-E)** Representative maximum intensity projection immunofluorescence images of EAAT1, EAAT2 and GS co-stained with GFAP and F-actin of rtTA-infected control HEFs (rtTA) at 5 weeks and untreated HEFs. (**F**) Absolute fluorescence intensity measurement of EAAT1, EAAT2 and GS staining in rtTA-infected control HEFs (rtTA), GFAP+ SAB-infected HEFs (SAB) and GFAP+ SB-infected HEFs (SB) at 5 weeks. **(G)** Representative maximum intensity projection immunofluorescence image of CX43 co-stained with GFAP and F-actin in SB-infected HEFs (SB) at 5 weeks. (**H)** Example immunofluorescence image of SB-HEF-iAs at 5 weeks showing spread of biocytin between S100B+ cell to S100B+ and GFAP+ cell. Arrowheads indicate cells with biocytin and thick full arrowhead biocytin-injected cell. Individual color levels adjusted for visibility (H). Scale bar: 50 µm (A, B, C, E, G, H). Data are presented as mean±SEM of n=72-211 cells from 3 independent viral transduction experiments. Kruskal-Wallis tests was performed for statistical analysis. *$p<0.05$. ****$p<0.0001$.

Figure S5. KIR4.1 immunocytochemistry, expression levels and ATP1B2 quantification. Related to Figure 4.
(A) Representative maximum intensity projection immunofluorescence images of potassium buffering protein KIR4.1 co-stained with GFAP and F-actin in rtTA-infected control HEFs (rtTA), SAB-infected HEFs (SAB) both at 5 weeks, and untreated HEFs. (**B**) Absolute intensity measurement KIR4.1 in rtTA-infected control HEFs (rtTA), GFAP+ SAB-infected HEFs (SAB) and GFAP+ SB-infected HEFs (SB) at 5 weeks. **(C)** Quantification of ATP1B2 expressing cells at 5 weeks related to total number of nuclei. **(D)** Quantification of ATP1B2 and GFAP co-expressing cells at 5 weeks related to total number of nuclei. Scale bar: 20 µm (A). Data are presented as mean±SEM of n=3-4 independent viral

transduction experiments done on separate days. Kruskal-Wallis tests were performed for statistical analysis. *p<0.05. ****p<0.0001.

Figure S6. Gene and transcription factor expression in SB-HEF-iAs. Related to Figure 5.
(A) Gene expression of *MKI67, GFAP, VIM* and *CD44* in SB-HEF-iAs at 5 weeks following 24 h treatment with TNFα, IL-1α and Cq1 as determined by RT-qPCR shown relative to untreated SB-HEF-iAs. **(B)** Quantification of SOX9 immunoreactive cells in S100B and GFAP negative cells (non-converted) and S100B and/or GFAP positive cells (converted) relative to number of nuclei in SB-transduced HEFs at 1 and 3 weeks. **(C)** Absolute fluorescence intensity of SOX9 immunoreactive cells in S100B and GFAP negative cells (non-converted) and S100B and/or GFAP positive cells (converted) in SB HEFs at 1 and 3 weeks. **(D)** Gene expression of forebrain, midbrain, ventral and spinal cord markers in rtTA-HEFs and SB-HEF-iAs at 5 weeks and human fetal cortical astrocytes (hAs) as determined by RT-qPCR shown relative to *GAPDH.* Data are plotted as mean$\pm$SEM of n=3-4 independent viral transduction experiments (A, B, D) or n=2 separate RNA isolations (hAs) (D). and median, 25th and 75th percentiles with Tukey whiskers of n=133-215 cells from 3 independent viral transduction experiments (C). Mann-Whitney test (B, C) and Kruskal-Wallis test (D) was performed for statistical analysis *p<0.05.

Figure S7. iN co-cultures with SB-HEF-iAs shows improved attachment to coverslips. Related to Figure 7.
(A-B) Brightfield images illustrating iN detachment in monocultures but not in co-cultures. Scale bar: 200 µm (A), 100 µm (B).

SUPPLEMENTAL TABLES

Table S1. Primary antibodies for immunocytochemistry.

Antigen	Dilution	Company	Catalog number
Rabbit anti-S100	1:400	Dako	Z0311
Rabbit anti-S100	1:1	Dako	GA504
Guinea pig anti-GFAP	1:500	Synaptic Systems	173004
Rabbit anti-GLT1	1:250	Invitrogen	701988
Rabbit anti-GLAST	1:200	Abcam	ab416
Rabbit anti-GS	1:1000	Abcam	ab49873
Rabbit anti-CX43	1:1000	Abcam	ab11370
Rabbit anti-CX43	1:100	Santa Cruz Biotechnology	sc9059
Rabbit anti-ALDH1L1	1:500	Abcam	ab87117
Rabbit anti-AQP4	1:100	Abcam	ab5971
Rabbit anti-KIR4.1	1:1000	Alomone labs	APC-035
Rabbit anti-ATP1B2	1:50	Atlas Antibodies	HPA010698
Mouse anti-β-TUBULIN III	1:500	Sigma-Aldrich	T8660
Chicken anti-MAP2	1:5000	Abcam	ab5392
Phallodin-TRITC	2.5 ug/ml	Sigma-Aldrich	P1951
Mouse anti-MYOD	1:250	BD Biosciences	554130
Mouse anti-Aminopeptidase N (ANP)	1:100	R&D Systems	MAB3815

Table S2. Secondary antibodies for immunocytochemistry.

Antigen	Dilution	Company	Catalog number
Donkey anti-rabbit AF488	1:500	Life Technologies	A21206
Donkey anti-mouse AF488	1:500	Life Technologies	A21202
Donkey anti-chicken AF488	1:500	Jackson Immunoresearch	703-545-155
Donkey anti-rabbit AF568	1:500	Life Technologies	A10042
Goat anti-guinea pig AF568	1:500	Life Technologies	A11075
Donkey anti-rabbit AF647	1:500	Life Technologies	A31573
Donkey anti-guinea pig AF647	1:500	Jackson Immunoresearch	706-605-148

Table S3. TaqMan assays used for RT-qPCR.

Gene	Assay ID
GAPDH	Hs02758991_g1
GJA1	Hs00748445_s1
S100B	Hs00902901_m1
GFAP	Hs00909233_m1
DCN	Hs00370384_m1
IL-6	Hs00174131_m1
CXCL10	Hs00171042_m1
CXCL8	Hs00174103_m1
CCL5	Hs00982282_m1
C3D	Hs00163811_m1
MKI67	Hs01032443_m1
CD44	Hs01075862_m1
VIM	Hs00958111_m1
MYBPC1	Hs00159451_m1
WIF1	Hs00183662_m1
HOXB4	Hs00256884_m1
HOXC8	Hs00224073_m1
HOXC6	Hs00171690_m1
OTX2	Hs00222238_m1
DMRT2A	Hs00294890_m1
CHRDL1	Hs01035484_m1
CRYM	Hs00157121_m1
NKX2.1	Hs00968940_m1

Table S4. TaqMan assays used for single cell gene expression analysis. Related to Figure 3.

Marks	Gene	Assay ID
House-keeping	*YWHAZ*	Hs03044281_g1
House-keeping	*GAPDH*	Hs02758991_g1
House-keeping	*UBC*	Hs00824723_m1
House-keeping	*ACTB*	Hs01060665_g1
Astrocytes	*SOX9*	Hs00165814_m1
Astrocytes	*NFIA*	Hs00325656_m1
Astrocytes	*NFIB*	Hs01029174_m1
Astrocytes	*ATF3*	Hs00231069_m1
Astrocytes	*ID4*	Hs00155465_m1
Astrocytes	*EZR*	Hs00931646_m1
Astrocytes	*SLC1A3*	Hs00188193_m1
Astrocytes	*RUNX2*	Hs01047973_m1
Astrocytes	*GPC4*	Hs00155059_m1
Fetal astrocytes	*NOTCH1*	Hs01062014_m1
Fetal astrocytes	*CNTFR*	Hs00181798_m1
Fetal astrocytes	*TNC*	HS01115665_m1
Fetal astrocytes	*TUBA1A*	Hs00362387_m1
Fetal astrocytes	*FABP7*	Hs00361426_m1
Fetal astrocytes	*NNAT*	Hs00193590_m1
Mature astrocytes	*KCNJ10*	Hs00158426_m1

Mature astrocytes	*ATP1B2*	Hs00155922_m1
Mature astrocytes	*ATP1A2*	Hs00265131_m1
Mature astrocytes	*ALDOC*	Hs00902799_g1
Mature astrocytes	*S100B*	Hs00902901_m1
Mature astrocytes	*IGFBP7*	Hs00266026_m1
Mature astrocytes	*SLC25A18*	Hs01017349_m1
Mature astrocytes	*SLC15A2*	Hs01113665_m1
Mature astrocytes	*SLC14A1*	Hs00998199_m1
Mature astrocytes	*F3*	Hs01076029_m1
Mature astrocytes	*HEPACAM*	Hs00404147_m1
Mature astrocytes	*MLC1*	Hs01126548_m1
Mature astrocytes	*FGFR3*	Hs00179829_m1
Mature astrocytes	*DIO2*	Hs00255341_m1
Mature astrocytes	*BMPR1B*	Hs01010965_m1
Mature astrocytes	*SLC4A4*	Hs00186798_m1
Fibroblasts	*DCN*	Hs00370384_m1
Fibroblasts	*ECM1*	Hs00189435_m1
Fibroblasts	*TSLP*	Hs00263639_m1
Fibroblasts	*TGFB1I1*	Hs00210887_m1
Fibroblasts	*VCAM1*	Hs01003372_m1
Proliferating cells	*MKI67*	Hs01032443_m1
Proliferating cells	*TOP2A*	Hs01032137_m1
Neural stem cells	*PAX6*	Hs00240871_m1
Neurons	*MYT1L*	Hs00903951_m1
Neurons	*SYP*	Hs00300531_m1
Oligodendrocytes	*OLIG2*	Hs00377820_m1
Oligodendrocytes	*MBP*	Hs00921945_m1

SUPPLEMENTAL EXPERIMENTAL PROCEDURES

Human tissue and cell culture

Human tissue was obtained from dead aborted human fetuses 6-9 week post-conception from Lund and Malmö University Hospitals. Developmental stage of the embryos was determined by crown-to-rump length and careful evaluation of external features of the embryos and internal features of the nervous system. Tissue was microdissected under a stereo-microscope (Leica) in ice-cold hibernation medium (Gibco). Human embryonic dermal fibroblasts (HEFs) were isolated after removal of the central nervous system, spinal ganglia and internal organs, residual tissue was transferred several times to a clean petri dish containing sterile cold hibernation medium to remove contaminant cells. The epidermis was then carefully removed by tearing the tissue with sterile forceps. Sub-dissected epidermis was washed several times with hibernation medium prior to enzymatic digestion with 0.25% Trypsin-EDTA (Sigma-Aldrich) at 37°C for 5 minutes and was manually triturated to reach a single cell suspension. Cells were plated onto 0.1% gelatin coated flasks (Sigma-Aldrich) in Fibroblast medium (DMEM, 10% FBS, 1% GlutaMAX, 1% NEAA and 1% Sodium Pyruvate, all from Gibco), passaged after reaching confluence with 0.25% Trypsin-EDTA and seeded in uncoated T25 or T75 flasks. HEFs were expanded for two passages, cryopreserved in 85% Fibroblast medium, 5% FBS and 10% DMSO (Sigma-Aldrich) and used for direct conversion experiments at passage 3-7. HEFs used for immunocytochemical analysis were after thawing expanded one passage, seeded on 0.167 mg/ml Matrigel-coated glass coverslips and fixed after 3 days.

Human fetal cortical astrocytes (hAs) were derived as previously described (Miskinyte et al., 2018) from fetuses 7-9 week post-conception and expanded in poly-D-lysine (Sigma-Aldrich)/ fibronectin (Life Technologies) coated flasks in Fetal astrocyte medium (DMEM-F12, 10% FBS 1% GlutaMAX and 1% N-2, all from Gibco). hAs were used at passage 5-8. hAs used for immunocytochemical analysis were seeded on 0.167 mg/ml Matrigel-coated glass coverslips and fixed after 3-4 days.

Postnatal fibroblasts were initially expanded according to the manufacturer instructions after which they were expanded and cryopreserved as described for HEFs.

Vectors and viral production

FUW-rtTA (Addgene #20342) and tetO-Ascl1-Puromycin (Addgene #97329) constructs were from AddGene. TetO-FUW-Sox9-Puromycin (Addgene #117269), tetO-FUW-Nfia-Blasticidin (Addgene #117270) and tetO-FUW-Nfib-Hygromycin (Addgene #117271) were generated as described (Canals et al., 2018). TetO-Ngn2-Hygromycin was a kind gift from Dr. M.Mall DFKZ Heidelberg. Lentiviral particles were produced as previously described (Quist et al., 2021).

Direct conversion

Selection antibiotics were titrated and following concentration used: *Nfia* alone blasticin 4 µg/ml, *Nfib* alone 50 µg/ml hygromycin, *Sox9* alone 1 µg/ml puromycin, *Sox9+Nfia* 1 µg/ml blasticin and 0.5 µg/ml puromycin, *Sox9+Nfib* 1 µg/ml puromycin and 50 µg/ml hygromycin, *Nfia+Nfib* 2 µg/ml blasticin and 50 µg/ml hygromycin and *Sox9+Nfia+Nfib* 1 µg/ml blasticin, 0.5-1 µg/ml puromycin and 25-100 µg/ml hygromycin.

Human embryonic and postnatal fibroblasts were seeded in 0.083 mg/ml Matrigel-coated 24-well plates (50,000 or 20 000 cells/well respectively) or 6-well plates (200,000 or 80,000 cells/well respectively) on day -2. For rtTA-infected controls half the number of cells were seeded due to their rapid proliferation without selection and rtTA-HEFs cultured in 6-well plates were split 1:6 using 0.25% Trypsin-EDTA at day 9-10, collected with FGF medium supplemented with 0.1% BSA (Gibco) and seeded in new Matrigel-coated 6-well plates.

For serum-containing condition the Fibroblast medium was changed every second or third day until day 21-23.

For Serum-free condition (1:1 DMEM-F12, HEPES:Neurobasal, 1% N-2, 1% GlutaMAX, 1% Sodium Pyruvate, 5 µg/ml N-Acetyl-L-cysteine (Sigma-Aldrich) and 5 µg/ml Heparin-binding EGF-like growth factor (Sigma-Aldrich)) and Serum-free + SM condition (10 ng/ml CNTF (Peprotech), 40 ng/ml hLIF (Peprotech), 40ng/ml BMP4 (Peprotech) and 500 µg/ml dbcAMP (Sigma-Aldrich)), the Serum-free medium (+SM) was gradually introduced after selection periods were finished at day 6. Thereafter, half of the culture medium was changed every second or third day.

For electrophysiological recordings, biocytin-injections and immunocytochemical analysis at day 37-42, cells were dissociated with accutase (Gibco) and seeded on 0.167 mg/ml Matrigel-coated glass coverslips (5,000-20,000 cells/coverslip) 4-7 days before analysis.

Astrocytic identity of obtained cells was analyzed at day 21-23 and day 33-42.

Immunocytochemistry

Cells were washed with KPBS, fixed in 4% paraformaldehyde in KPBS for 20 min at room temperature (RT) and washed with KPBS. Blocking and permeabilization was performed with 0.025 % Triton X-100 in KPBS containing either 5% normal donkey serum (NDS) (Millipore) or 2.5% NDS and 2.5% normal goat serum (NGS) (Millipore) (blocking solution) for 1 h at RT. Primary antibodies were applied overnight at 4°C in blocking solution. Cells were washed two times with 0.025 % Triton X-100 and once with blocking solution for 5 min each. Secondary antibodies and 1 µg/ml Hoechst 33342 (Invitogen) were applied for 2 h at RT in blocking solution. For F-actin staining Phallodin-TRITC were applied with secondary antibodies. Cells were washed with 0.025% Triton X-100 for 5 min, if DAPI (Sigma-Aldrich) was used for nuclear staining it was included at 0.5 µg/ml in this wash, followed by two washes with KPBS for 5 min after which wells were filled with KPBS containing 0.1% sodium azide (Sigma-Aldrich) and coverslips washed once with MilliQ and mounted on glass slides using Dabco:PVA. Primary and secondary antibodies used are listed in Table S1 and S2.

Image acquisition, processing and quantification

Images were acquired using Olympus BX61 or Olympus IX73 using CellSens software or Zeiss LSM 780 confocal microscope using Zen software. HDR was used for acquisition of punctate protein expression. Brightness and contrast were adjusted for entire images, Individual color levels only when needed to reflect what was observed at the microscope and if so indicated in figure legends.

Images for quantifications were acquired using Olympus IX51 and IX73 inverted microscopes with a 20X magnification and CellSens software. The number of S100B and GFAP immunoreactive cells were counted in 10-20 randomly selected fields of view in one to two wells of each independent viral transduction experiments. Exposure times were set to be as similar as to the intensity as when viewed directly with the microscope and were kept constant for acquisition of all wells in each infection and staining. Images of secondary antibody only controls were acquired with the same exposure settings used during each acquisition session. Quantification of nuclei and immunoreactive cells were performed in ImageJ. Total number of nuclei and immunoreactive cells in each well was quantified based on the area of the wells and the fields of view area. For ATP1B2 quantifications at least 100 cells for each independent viral transduction were counted.

The fluorescence intensity measurements were performed on at least three independent biological replicates for each experimental condition. For each replicate, all images were acquired using constant parameters. The analysis was performed as absolute fluorescence intensity measurement for cells identified as GFAP positive in SAB-HEF-iAs and SB-HEF-iAs conditions in at least four different fields randomly acquired from the culture. Imaging corrections were performed for both camera noise and for field illumination for all acquired fields.

Glutamate uptake assay

rtTA-infected, SAB or SB-infected HEFs or human adult fibroblasts were cultured in 6-well plates and analyzed at 33-34 days after induction. Human fetal astrocytes used as controls were cultured on PDL- and fibronectin coated T25 flasks or Matrigel-coated Ibidi dishes. Cells were washed three times with HBSS (Mg2+ and Ca2+) (Gibco) after which 125 µM Glutamic acid (Sigma-Aldrich) in HBSS was applied. Samples were collected after 30 min and kept at 4°C until analysis. Amount of glutamate was measured using a Fluorometric kit (Abcam ab138883). Measured absorbances for standards and samples were corrected using a blank sample measurements and HBSS respectively. Glutamate uptake was determined by comparing the amount of glutamate in the media to the initial concentration. All standards and samples were measured in two technical replicates.

Electrophysiology

For whole cell patch-clamp recordings, coverslips were transferred to the recording chamber and constantly perfused with carbogenated artificial cerebrospinal fluid (ACSF), containing (in mM): 119 NaCl, 2.5 KCl, 1.3 $MgSO_4$, 2.5 $CaCl_2$, 26 $NaHCO_3$, 1.25 NaH_2PO_4, and 11 glucose (pH ~7.4, osmolarity ~305 mOsm). Patch pipette was filled with internal solution containing (in mM): K-gluconate 122.5, KCl 17.5, NaCl 8, KOH-HEPES 10, KOH-EGTA 0.2, MgATP 2, and Na3GTP 0.3 (pH ~7.2, 295 mOsm). Average pipette tip resistance was ~4-5 MΩ and recordings were done at 34˚C. Pipette current was corrected online before gigaseal formation while fast capacitive currents were compensated for during cell-attached configuration. All recordings were done using a HEKA double patch EPC10 amplifier (HEKA Elektronik, Lambrecht, Germany) and sampled at 10 KHz. PatchMaster software was used for data acquisition and Fitmaster, IgorPro and NeuroMatic (Rothman and Silver, 2018) for offline analysis.

Single cell recordings of SAB/SB-HEF-iAs were performed at day 37-42 and compared to untreated HEFs and human fetal cortical astrocytes. 5,000-10,000 cells were plated on glass coverslips 3-9 days before patch clamp recordings. Only S100B+ or GFAP+ SAB/SB-HEF-iAs, as identified with biocytin co-labeling or SAB/SB-HEF-iAs with a clear astrocytic morphology were included in analysis. SAB-HEF-iAs from two independent viral transduction and SB-HEF-iAs from four independent viral transductions were used.

Resting membrane potential (RMP) was measured in current clamp mode at 0 pA immediately after establishing whole-cell configuration. Series resistance and input resistance (Ri) were calculated from a 5, or 10 mV pulse and monitored throughout the experiment. Whole-cell currents were induced in voltage-clamp mode either by delivering voltage steps in 10 mV increments for 200 milliseconds (ms) at a holding potential of −80 mV, or a protocol that, from the holding potential of −60 mV, stepped the membrane potential to −100 mV for 200 ms before a 100 ms long depolarizing ramp to +120 mV. To minimize recording errors, series resistance was compensated to 60-80% and recordings with leak currents >100pA were excluded. Moreover, quality of recording was monitored with a test pulse throughout the recording.

Electrophysiological properties of iNs were recorded at day 45-52. RMP, Ri and series resistance was measured similar to above described. Ability to generate action potential (AP) was determined by 500 ms square depolarizing current step injections at RMP, with 10 pA increments and ramp injection of 1 second depolarizing current, that was also used to determine action potential threshold. AP amplitude was measured from threshold to peak, the half AP amplitude width was determined as the time between the rising and decaying phase of the AP measured at half the amplitude of the AP, and the afterhyperpolarization (AHP) amplitude was determined as the difference between the AHP peak and the AP threshold. Whole-cell sodium and potassium currents were observed in voltage-clamp mode at a holding potential of −70 mV and 200ms voltage steps were delivered in 10 mV increments.

Biocytin labelling
To confirm identity of recorded cells for astrocyte experiments and visualize diffusion through gap junctions individual cells were filled with biocytin (1-3 mg/ml, Biotium in internal solution) using a patch pipette during electrophysiology recordings. To detect biocytin post hoc, Alexa Fluor 568 conjugated Streptavidin (ThermoFisher Scientific) diluted 1:500 in 0.025% TKPBS with 5% NDS was added for 1h at RT and co-stained with S100B or GFAP antibody. SAB-HEF-iAs from two independent viral transductions and SB-HEF-iAs from four independent viral transductions were used.

Gene Expression
For RT-qPCR analysis cells were at day 33-34 washed once with DPBS (-/-) (Gibco) before RNA was collected and purified using RNeasy® Mini Kit (Qiagen). DNase I (Qiagen) digestion was performed to prevent DNA contamination. RNA concentration and purity were determined with a Nanodrop ND-1000 Spectrophotometer (Saveen & Werner). cDNAs were prepared using qScript cDNA Synthesis Kit (Quanta Biosciences). For each sample two to three technical replicates were performed as well as a no reverse transcriptase reaction (NRT) to exclude DNA contamination. 50 ng cDNA were used for each qPCR reaction and was performed using TaqMan Universal PCR Master Mix and TaqMan Gene Expression Assays (both ThermoFisher Scientific) (Table S3) on an iQ5 real-time PCR detection system (Biorad). For each TaqMan Assay a no-template control was included. Data are expressed relative to the house-keeping gene GAPDH using the ΔCt method.

Single-cell RT-qPCR analysis was done as previously described (Miskinyte et al., 2017). Genes were selected from multiple sources based on reported expression in fibroblasts, fetal and mature astrocytes, neural stem cells, neurons and oligodendrocytes (complete list in Table S4) (Allen et al., 2012; Khazaei et al., 2018; Molofsky and Deneen, 2015; Philippeos et al., 2018; Sloan et al., 2017; Tiwari et al., 2018; Uhlén et al., 2015; Zhang et al., 2016). In brief, single viable (based on DRAQ7 (Abcam) staining) rtTA-infected and SAB-infected HEFs at day 21-23 from four independent viral transductions and human fetal astrocytes were sorted in a FACSAria II (BD Biosciences) to individual wells of 96-well plates containing lysis buffer. Linearity controls (0-, 10- and 20-cells) were included in each plate. Plates were snap frozen and stored at -80°C until analysis. RT-PCR pre-amplification were done using CellsDirect™ One-Step qRT-PCR Kit (ThermoFisher Scientific) and TaqMan assays (ThermoFisher Scientific). XenoRNA™ Control (TaqMan™ Cells-to-CT™ Control Kit, ThermoFisher Scientific) and 3 NRT controls were included to control for presence of RT-PCR inhibitors and DNA contamination respectively. cDNA levels were determined using the BioMark dynamic array system (Fluidigm). Amplification curves were manually inspected in the Fluidigm Real-Time PCR Analysis software to ensure proper loading and reading of the chips. Data was analyzed with the Auto (Global) Ct threshold method using a Linear (Derivate) baseline correction and quality threshold set at 0.6 and further filtered and processed using the SCExV webtool (Lang et al., 2015). Cells with non-detectable (Ct>25) levels of house-keeping gene UBC and genes with detectable levels (Ct<25) in NRT controls or presence of failed amplification curves were excluded as well as house-keeping genes, xRNA and 0-, 10- and 20-cells controls. Data was normalized to the median Ct of each cell, Ct-values inverted, z-scored normalized by gene, non-detectable gene expression was artificially set to the minimum of the z scored values minus 1 and hierarchical clustering analysis performed using ward.D algorithm to clustered based on correlation distance. Groups were manually colored based on condition (rtTA-infected HEFs, SAB-infected HEFs and human fetal astrocytes respectively) to generate heat map, violin plots and DDRTree.

Cytokine stimulation
30 ng/mL TNFα (R&D Systems), 3 ng/mL IL-1α (Sigma-Aldrich) and 400 ng/mL C1q (MyBioSource) were applied in a full media change. 24 hours after stimulation cells were either fixed for immunocytochemistry or RNA was collected for RT-qPCR.

Calcium imaging
rtTA-HEFs, SB-HEF-iAs and human fetal astrocytes were cultured in 6w plates and imaged in DMEM/F12 without phenol red containing 25mM HEPES (both from ThermoFisher Scientific). Cells were washed once with DMEM/F12, loaded with 2 µM Fluo4 (Life Technologies) prepared in Pluronic Acid (Invitrogen) in DMEM/F12 for 30 min at 37 C after which a full media change to 2 ml DMEM/F12 was performed. Imaging was done using Olympus IX73 microscope with a 10X objective at 1Hz for 5 min. Baseline recording were done for 60 s after which 500 µl 90 µM ATP was added next to the imaging field. Data was analyzed using Fiji-ImageJ software. Ca^{2+} signals are presented as relative fluorescence changes (dF/F0) from specified regions of interest (ROIs). Single ROIs were manually selected based on overlays of Fluo-4 fluorescence maximum intensity projection and brightfield

images of the recorded field. Data was corrected for background intensity levels at each time point based on the signal detected in an area not containing any cells. Calcium elevation events were detected with thresholds of 3 times the standard deviation from baseline, which was set as the time interval preceding the stimulus. In experiments that presented spontaneous Ca^{2+} oscillations in the baseline time window, F0 was calculated in the time interval in between spontaneous transients. For post hoc identification of S100B and GFAP expressing cells ROI selection was manually done on immunocytochemical images of recorded fields.

Generation of iNs and iAs/iN co-cultures

HEF-iNs and SB-HEF-iAs were generated in parallel using the same starting population of HEFs. Co-cultures were set up at day 9-10.

iNs were generated by transducing HEFs with rtTA, tetO-Ascl1-Puromycin and tetO-Ngn2-Hygromycin following the same procedure as described for iAs until day 1 when a 3-day selection period with puromycin (1 µg/ml) and 5-day selection period with hygromycin (50 µg/ml) was started. On day 3, 1:1 Fibroblast medium and Early iN medium (1:1 DMEM-F12, HEPES:Neurobasal, 1% GlutaMAX, 2% B-27 and 1% N-2 supplemented with 1 µg/ml mouse laminin (Gibco), 400 µg/ml db-cAMP, 150 µg/ml Noggin (Peprotech), 10 µM SB431542 (Peprotech), 3 µM CHIR99021 (Sigma-Aldrich), 5 µM Forskolin (Peprotech), 0.5 µM LDN-193189 (Selleck) and 0.5 µM A83-1 (StemCell Technologies) were added. At day 4 a full medium change to Early iN medium was performed after which half of the medium was changed every 2nd to 3rd day. At day 9-10 HEF-iN and SB-HEF-iAs were seeded together. Cells were washed with DPBS (-/-), dissociated using accutase containing 100 U/ml DNase I (Roche), collected with FGF medium/Early iN containing 0.1% BSA and 100U/ml DNase I and pelleted by centrifugation (5 min, 300 rcf). 10,000-20,000 SB-HEF-iAs were seeded on poly-D-lysine/laminin-coated glass coverslips by applying cell suspensions as a drop and allowed to attach after which HEF-iNs were passed through 40 µm strainer and 60,000-80,000 HEF-iNs were seeded in a 1:1 FGF medium:Early iN medium alone or on top of SB-HEF-iAs as a drop. Once cells had attached medium were filled to 500 µl. The day after a full medium change were performed after which half medium changes were done every second to third day. At day 11, FGF medium was replaced with Serum-free medium supplemented with 10 ng/ml CNTF, 10 ng/ul BMP4 and 500 µg/ml dbcAMP and at day 18 Early iN medium was replaced by Late iN medium (1:1 DMEM-F12, HEPES:Neurobasal, 1% GlutaMAX, 2% B-27 and 1% N-2 supplemented with 1 µg/ml mouse laminin, 500 µg/ml db-cAMP, 10ng/ml GDNF (Peprotech) and 10 ng/ml NT3 (Peprotech). If signs on detachment were observed 10 µg/ml laminin were added when changing medium.

Statistical analysis

Statistical analysis was performed using GraphPad Prism (version 7.02-9.2.0). Statistical significance was determined by the following statistical tests; S100B and GFAP quantifications two-tailed paired t-test and Kruskal-Wallis test, ATP1B2 and SOX9 quantifications Mann-Whitney test, RT-qPCR data Mann-Whitney test and Kruskal-Wallis test, glutamate uptake Kruskal-Wallis test and two-tailed Wilcoxon matched-pairs rank test, cell number quantifications and KI67 quantifications Friedman test, fluorescence intensity measurements and calcium signaling data Kruskal-Wallis test. For electrophysiological recordings of uninfected HEFs, SAB-HEF-iAs, SB-HEF-iAs and hAs Kruskal-Wallis tests were performed except for the current/voltage (I/V) curve where statistical significance was analyzed using multiple unpaired t-tests. For electrophysiological recordings of iNs unpaired t-tests were performed. All Kruskal-Wallis tests were followed by Dunn’s multiple comparison test. Significance level of 0.05 were used in all statistical tests.

SUPPLEMENTAL REFERENCES

Allen, N.J., Bennett, M.L., Foo, L.C., Wang, G.X., Chakraborty, C., Smith, S.J., and Barres, B.A. (2012). Astrocyte glypicans 4 and 6 promote formation of excitatory synapses via GluA1 AMPA receptors. Nature *486*, 410-4. 10.1038/nature11059.

Canals, I., Ginisty, A., Quist, E., Timmerman, R., Fritze, J., Miskinyte, G., Monni, E., Hansen, M.G., Hidalgo, I., Bryder, D., et al. (2018). Rapid and efficient induction of functional astrocytes from human pluripotent stem cells. Nat Methods *15*, 693-6. 10.1038/s41592-018-0103-2.

Khazaei, N., Rastegar-Pouyani, S., O'Toole, N., Wee, P., Mohammadnia, A., and Yaqubi, M. (2018). Regulating the transcriptomes that mediate the conversion of fibroblasts to various nervous system neural cell types. J Cell Physiol *233*, 3603-14. doi:10.1002/jcp.26221.

Lang, S., Ugale, A., Erlandsson, E., Karlsson, G., Bryder, D., and Soneji, S. (2015). SCExV: a webtool for the analysis and visualisation of single cell qRT-PCR data. BMC Bioinformatics *16*, 320. 10.1186/s12859-015-0757-z.

Miskinyte, G., Devaraju, K., Gronning Hansen, M., Monni, E., Tornero, D., Woods, N.B., Bengzon, J., Ahlenius, H., Lindvall, O., and Kokaia, Z. (2017). Direct conversion of human fibroblasts to functional excitatory cortical neurons integrating into human neural networks. Stem Cell Res Ther *8*, 207. 10.1186/s13287-017-0658-3.

Miskinyte, G., Gronning Hansen, M., Monni, E., Lam, M., Bengzon, J., Lindvall, O., Ahlenius, H., and Kokaia, Z. (2018). Transcription factor programming of human ES cells generates functional neurons expressing both upper and deep layer cortical markers. PLoS One *13*, e0204688. 10.1371/journal.pone.0204688.

Molofsky, A.V., and Deneen, B. (2015). Astrocyte development: A Guide for the Perplexed. Glia *63*, 1320-9. 10.1002/glia.22836.

Philippeos, C., Telerman, S.B., Oulès, B., Pisco, A.O., Shaw, T.J., Elgueta, R., Lombardi, G., Driskell, R.R., Soldin, M., Lynch, M.D., et al. (2018). Spatial and Single-Cell Transcriptional Profiling Identifies Functionally Distinct Human Dermal Fibroblast Subpopulations. J Invest Dermatol *138*, 811-25. 10.1016/j.jid.2018.01.016.

Quist, E., Ahlenius, H., and Canals, I. (2021). Transcription Factor Programming of Human Pluripotent Stem Cells to Functionally Mature Astrocytes for Monocultures and Cocultures with Neurons. In Methods Mol Biol, H. Ahlenius, ed. (New York, NY: Humana), pp. 133-48.

Rothman, J.S., and Silver, R.A. (2018). NeuroMatic: An Integrated Open-Source Software Toolkit for Acquisition, Analysis and Simulation of Electrophysiological Data. Front Neuroinform *12*, 14. 10.3389/fninf.2018.00014.

Sloan, S.A., Darmanis, S., Huber, N., Khan, T.A., Birey, F., Caneda, C., Reimer, R., Quake, S.R., Barres, B.A., and Pasca, S.P. (2017). Human Astrocyte Maturation Captured in 3D Cerebral Cortical Spheroids Derived from Pluripotent Stem Cells. Neuron *95*, 779-90. 10.1016/j.neuron.2017.07.035.

Tiwari, N., Pataskar, A., Péron, S., Thakurela, S., Sahu, S.K., Figueres-Oñate, M., Marichal, N., López-Mascaraque, L., Tiwari, V.K., and Berninger, B. (2018). Stage-Specific Transcription Factors Drive Astrogliogenesis by Remodeling Gene Regulatory Landscapes. Cell Stem Cell *23*, 557-71. 10.1016/j.stem.2018.09.008.

Uhlén, M., Fagerberg, L., Hallström, B.M., Lindskog, C., Oksvold, P., Mardinoglu, A., Sivertsson, Å., Kampf, C., Sjöstedt, E., Asplund, A., et al. (2015). Proteomics. Tissue-based map of the human proteome. Science *347*, 1260419. 10.1126/science.1260419

Zhang, Y., Sloan, S.A., Clarke, L.E., Caneda, C., Plaza, C.A., Blumenthal, P.D., Vogel, H., Steinberg, G.K., Edwards, M.S., Li, G., et al. (2016). Purification and Characterization of Progenitor and Mature Human Astrocytes Reveals Transcriptional and Functional Differences with Mouse. Neuron *89*, 37-53. 10.1016/j.neuron.2015.11.013.
