# Supplementary material for: Transcription factor-based direct conversion of human fibroblasts to functional astrocytes
Source: Stem Cell Reports. 2022 Jun 23;17(7):1620–35. doi: 10.1016/j.stemcr.2022.05.015 (PMC9287681; doi:10.1016/j.stemcr.2022.05.015)
Supplement: Document S2. Article plus supplemental information [file mmc5.pdf]

Stem Cell Reports
Article
OPEN ACCESS

Transcription factor-based direct conversion of human fibroblasts to functional astrocytes

Ella Quist,[1,2,*] Francesco Trovato,[1,2,3] Natalia Avaliani,[2] Oskar G. Zetterdahl,[1,2,4] Ana Gonzalez-Ramos,[5] Marita G. Hansen,[2] Merab Kokaia,[5] Isaac Canals,[4] and Henrik Ahlenius[1,2,*]
[1]Lund University, Faculty of Medicine, Department of Clinical Sciences Lund, Neurology, Stem Cells, Aging and Neurodegeneration, Lund, Sweden
[2]Lund Stem Cell Center, Lund, Sweden
[3]Lund University, Skane University Hospital, Department of Clinical Sciences Lund, Neurosurgery, Lund, Sweden
[4]Lund University, Faculty of Medicine, Department of Clinical Sciences Lund, Neurology, Glial and Neuronal Biology, Lund, Sweden
[5]Lund University, Skane University Hospital, Department of Clinical Sciences Lund, Epilepsy Center, Lund, Sweden
*Correspondence: ella.quist@med.lu.se (E.Q.), henrik.ahlenius@med.lu.se (H.A.)
https://doi.org/10.1016/j.stemcr.2022.05.015

SUMMARY

Astrocytes are emerging key players in neurological disorders. However, their role in disease etiology is poorly understood owing to inaccessibility of primary human astrocytes. Pluripotent stem cell-derived cells fail to mimic age and due to their clonal origin do not mimic genetic heterogeneity of patients. In contrast, direct conversion constitutes an attractive approach to generate human astrocytes that capture age and genetic diversity. We describe efficient direct conversion of human fibroblasts to functional induced astrocytes (iAs). Expression of the minimal combination *Sox9* and *Nfib* generates iAs with molecular, phenotypic, and functional properties resembling primary human astrocytes. iAs could be obtained by conversion of fibroblasts covering the entire human lifespan. Importantly, iAs supported function of induced neurons obtained through direct conversion from the same fibroblast population. Fibroblast-derived iAs will become a useful tool to elucidate the biology of astrocytes and complement current *in vitro* models for studies of late-onset neurological disorders.

INTRODUCTION

Astrocytes are a major cell type in the mammalian central nervous system (CNS) and are crucial for brain functionality (Molofsky et al., 2012). They provide trophic and functional support to neurons, take up and recycle neurotransmitters, and modulate neurotransmission (Allen and Eroglu, 2017). Astrocytes maintain brain homeostasis, form functional syncytia, communicate intra- and intercellularly, and respond to injury and inflammation (Verkhratsky and Nedergaard, 2018).

With these essential roles, it is not surprising that astrocyte dysfunction is associated with a range of neurological disorders. However, there is poor understanding of molecular roles of astrocytes and their interplay with other CNS cells in disease contexts (Molofsky et al., 2012). Traditionally, rodents have been used to study astrocyte biology but interspecies differences raise concerns regarding translational potential to humans (Zhang et al., 2016). Another hurdle is limited accessibility of astrocytes from living healthy individuals and patients with neurological diseases. However, recent advances in stem cell technology have emerged to generate human astrocytes for disease modeling.

Recently, we developed efficient generation of functional astrocytes from human pluripotent stem cells (Canals et al., 2018). However, for modeling aging, sporadic, or late-onset diseases, direct conversion protocols bypassing pluripotency are needed, as they preserve genetic mosaicism of the starting population and retain age-related features (Mertens et al., 2016). In addition, direct conversion avoids ethical concerns regarding embryonic stem cells and circumvents the need for reprogramming to pluripotency, making direct conversion an easy and faster approach to obtain patient-derived astrocytes. However, generation of induced astrocytes (iAs) by direct conversion from human fibroblasts has only been briefly explored (Caiazzo et al., 2015; Tian et al., 2016) remains inefficient and functional characterization is scarce.

Here, we describe substantially improved generation of iAs from human fibroblasts obtained from individuals covering the entire lifespan. In addition, we demonstrate that iAs are phenotypically and functionally similar to native human astrocytes. We also show for the first time functional co-culture of iAs and induced neurons (iNs) obtained from the same human starting fibroblast population, which constitutes a powerful tool for the study of astrocyte-neuron interactions and for disease modeling.

RESULTS

Sox9, *Nfia*, and *Nfib* convert human embryonic fibroblasts to induced astrocytes

Gliogenic transcription factors or small molecules have been shown to convert mouse fibroblasts to induced astrocytes (Caiazzo et al., 2015; Tian et al., 2016). However, conversion of human fibroblasts has not been studied in detail, and efficiency in generating mature functional human astrocytes remains low.

 © 2022 The Authors.
This is an open access article under the CC BY license (http://creativecommons.org/licenses/by/4.0/).

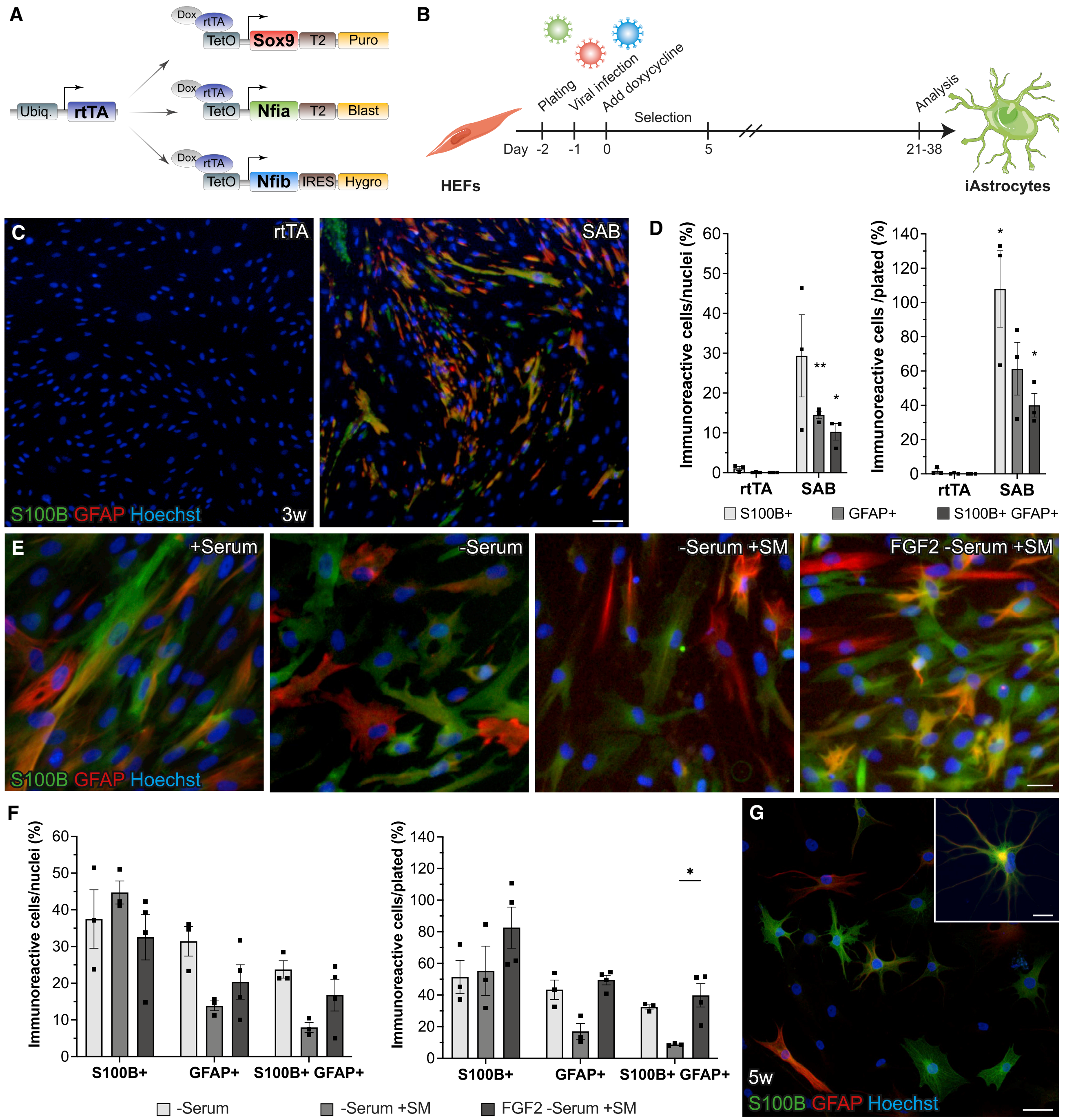

Figure 1. *Sox9*, *Nfia*, and *Nfib* convert human embryonic fibroblasts to induced astrocytes

(A) Schematic of doxycycline-inducible lentiviral constructs.

(B) Experimental overview of direct conversion to induced astrocytes (iAs).

(C) Representative immunofluorescence images of GFAP and S100B in rtTA- and SAB-infected HEFs at 3 weeks.

(D) Quantification of S100B, GFAP, and S100B/GFAP immunoreactive cells at 3 weeks related to total number of nuclei (purity) and plated cells (yield).

(E) Representative immunofluorescence images of SAB-HEF-iAs at 3 weeks converted in serum-containing medium (+Serum), serum-free medium (−Serum), serum-free medium with small molecules (−Serum + SM), or low-serum-containing medium with SM and FGF2 followed by serum-free medium with SM (FGF2 − Serum + SM).

(legend continued on next page)

To improve this, we cloned cDNAs of *Sox9*, *Nfia*, and *Nfib* (SAB), able to convert mouse fibroblasts, into doxycycline-inducible lentiviral vectors containing puromycin, blasticidin, and hygromycin resistance genes, respectively (Canals et al., 2018), to enable the selection of cells with high expression of transgenes. We then infected human embryonic fibroblasts (HEFs) with a vector constitutively expressing reverse tetracycline-controlled transactivator (rtTA) alone, as control, or together with SAB vectors, induced expression using doxycycline, and the day after started a 5-day selection period (Figures 1A and 1B). Cells were kept in serum-containing medium commonly used for primary mouse astrocytes. Three weeks after induction, we performed immunocytochemistry for canonical astrocyte markers S100B and glial fibrillary acidic protein (GFAP). Strikingly, we observed around 30% and 15% of SAB-infected HEFs expressing S100B and GFAP, respectively, and 10% co-expressed S100B and GFAP, which corresponded to a yield of 110% and 60% of S100B- and GFAP-expressing cells, while the yield of S100B and GFAP co-expressing cells was 40% (Figures 1C and 1D). This is in stark contrast to previous conversion of neonatal fibroblasts whereby the yield was only 2% (Caiazzo et al., 2015). rtTA-infected controls never expressed GFAP and only very rarely low-expressing S100B cells were found (Figure 1C).

We observed that the majority of $S100B^+$ and $GFAP^+$ cells had elongated or polygonal fibroblast-like morphology (Figures 1C and 1E) resembling human primary astrocytes cultured with serum but normally not present *in vivo* (Zhang et al., 2016). We hypothesized that introducing serum-free medium adapted for astrocytes as well as supplementation with small molecules important for astrogliogenesis could improve conversion and morphology of converted cells. To address this we tested three different conditions: (1) serum-free medium alone (Serum-free); (2) serum-free medium with leukemia inhibitory factor, bone morphogenetic protein 4 (BMP4), ciliary neurotrophic factor (CNTF), and cyclic AMP (cAMP) (Serum-free + SM); and (3) an initially low concentration (0.2%) of serum-containing medium with fibroblast growth factor 2 (FGF2), together with BMP4 and CNTF between days 4 and 11 followed by serum-free medium with BMP4, CNTF, and cAMP (FGF2 + Serum-free + SM).

By gradually switching to Serum-free medium, many $S100B^+$ and $GFAP^+$ SAB-infected HEFs developed a star-like morphology with thick processes. However, there was still a substantial number of cells displaying elongated or polygonal morphology. Interestingly, in Serum-free + SM medium, $S100B^+$ and $GFAP^+$ cells displayed smaller somas and more thin, branched processes. Strikingly, FGF2 + Serum-free + SM medium resulted in a larger proportion of $S100B^+$ and $GFAP^+$ cells that developed morphologies similar to that of stellate primary astrocytes (Figure 1E). Furthermore, quantification revealed that this condition resulted in the highest yield of converted cells, with more than 80% $S100B^+$, around 50% $GFAP^+$, and 40% $S100B^+/GFAP^+$ cells (Figure 1F).

We obtained slightly higher purity of $GFAP^+$ and S100B/GFAP co-expressing cells using Serum-free medium without SM (Figure 1F) but reasoned that the overall higher yield of cells expressing S100B and GFAP, and improved astrocytic morphology when using FGF2 + Serum-free + SM medium, was the best condition and was therefore used in subsequent experiments. Finally, by culturing SAB-infected HEFs for an additional 2 weeks some cells developed clear stellate astrocytic morphologies (Figure 1G). In addition, 31% expressed ATP1B2, described to be enriched in astrocytes (Zhang et al., 2016). However, expression was not specific to converted cells, as 15% of control cells also expressed ATP1B2 (Figures S1A and S1B).

Taken together, these findings indicate that selection of cells expressing high levels of transcription factors and optimization of media enables effective direct conversion of human fibroblasts to astrocytes, which we hereafter refer to as SAB-HEF-iAs.

Human induced astrocytes are functional

Astrocytes take up glutamate and convert to glutamine to prevent toxic accumulation and maintain synaptic transmission (Allen and Eroglu, 2017). To assess this crucial function in SAB-HEF-iAs, we analyzed the expression of glutamate transporters EAAT1, EAAT2, and glutamine synthetase (GS), responsible for glutamate-glutamine conversion (Verkhratsky and Nedergaard, 2018). We found that the vast majority of SAB-HEF-iAs expressed GS and were decorated with EAAT1 and EAAT2 puncta, although the latter was expressed at low levels (Figures 2A–2C). Next we assessed glutamate uptake. Importantly, SAB-HEF-iAs took up glutamate to similar extent as primary human fetal astrocytes and significantly more than rtTA-infected control HEFs (rtTA-HEFs) (Figure 2D).

(F) Quantification of purity and yield of S100B, GFAP, and S100B/GFAP immunoreactive cells at 3 weeks.
(G) Immunofluorescence image of SAB-HEF-iAs at 5 weeks converted in FGF2 − Serum + SM medium.
Puro, puromycin resistance gene; Blast, blasticidin resistance gene; Hygro, hygromycin resistance gene; HEFs, human embryonic fibroblasts. Individual color levels are adjusted for visibility in (C), (E), and (G). Data are presented as mean ± SEM of n = 3–4 independent experiments. Two-tailed paired t tests (D) and Kruskal-Wallis test (F) were performed for statistical analysis. $^*p < 0.05$, $^{**}p < 0.01$. Scale bars, 100 μm (C), 25 μm (E), 50 μm (G), and 20 μm (G, inset).

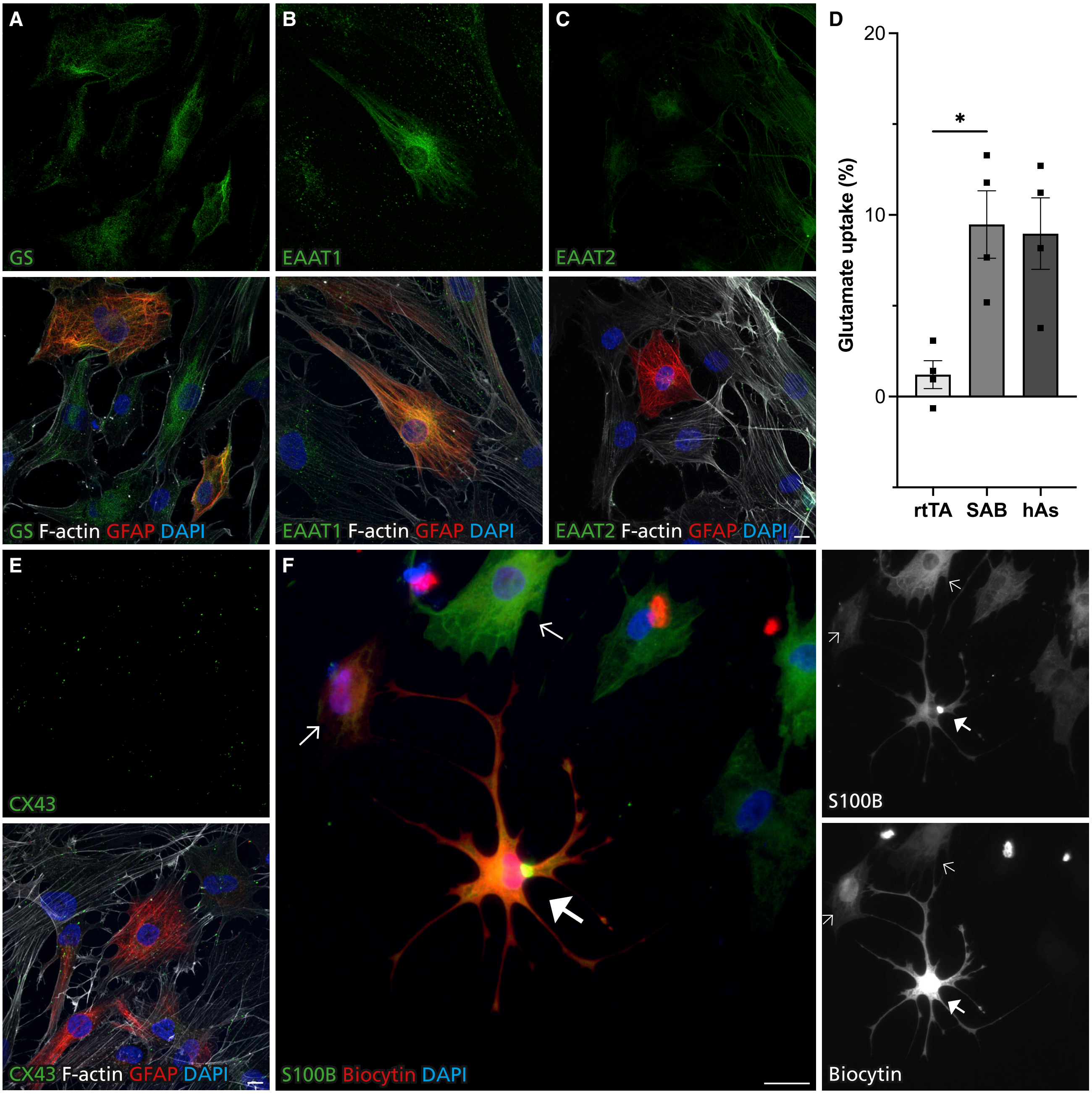

Figure 2. iAs express canonical astrocytic proteins and perform astrocytic functions

(A–C) Representative maximum-intensity projection immunofluorescence images of GS and glutamate transporters in SAB-HEF-iAs.
(D) Glutamate uptake of rtTA-infected control HEFs, SAB-HEF-iAs, and primary human fetal astrocytes (hAs).
(E) Representative maximum-intensity projection immunofluorescence images of CX43 in SAB-HEF-iAs.
(F) Example immunofluorescence image of SAB-HEF-iAs showing spread of biocytin between $S100B^+$ cells. Arrows indicate cells with biocytin and thick full arrow the injected cell. Individual color levels are adjusted for visibility.
Data are presented as mean ± SEM of n = 4 independent viral transduction experiments at 5 weeks. Kruskal-Wallis test was performed for statistical analysis in (D). *p < 0.05. Scale bars, 10 μm (A, B, C, and E) and 25 μm (F). See also Figure S1.

Another important property of astrocytes is to form functional syncytia through gap-junction coupling, essential for buffering ions and metabolites to maintain brain homeostasis (Sofroniew and Vinters, 2010). To investigate this, we first analyzed the presence of the gap-junction protein CX43 and found that $GFAP^+$ SAB-HEF-iAs expressed

CX43+ puncta (Figure 2E). Next, we injected single cells with biocytin, a dye that diffuses through functional gap junctions. We observed that injected S100B+ SAB-HEF-iAs spread biocytin to neighboring S100B+ SAB-HEF-iAs (Figure 2F) as well as non-converted fibroblasts. In line with previous reports (Zhang and Cui, 2017), rtTA-HEFs expressed CX43, confirmed using two different antibodies as well as by qRT-PCR, and biocytin spread also occurred between these cells (Figures S1C–S1F).

Taken together, these findings indicate that SAB-HEF-iAs perform key astrocytic functions.

Human induced astrocytes show transcriptional similarity to primary human astrocytes

Despite efficient conversion, SAB transduced cultures likely consist of both iAs and non-converted cells. To investigate molecular phenotypes and heterogeneity of SAB-infected HEFs, we performed single-cell gene expression. We analyzed the expression of 30 genes, enriched in astrocytes, fibroblasts, or neural lineages, in 20 rtTA-infected HEFs, 78 SAB-infected HEFs, and 23 human primary fetal astrocytes. As expected, we found primary astrocytes to express a number of astrocytic genes not expressed in rtTA-HEFs, which on the other hand expressed the fibroblast-enriched gene *DCN* (Philippeos et al., 2018). This allowed us to identify several populations within SAB-infected HEFs. One expressed astrocyte genes (*S100B* and *FABP7*) but not *DCN*. Another expressed both astrocyte genes and *DCN*, and one population expressed only *DCN* (Figures 3A and 3B). Very few cells expressed neural progenitor (*PAX6*) or neuronal (*SYP*) genes (Figure 3A) and we never observed any immunoreactivity for βIII-TUBULIN (data not shown), excluding that conversion induced a neuronal fate. Furthermore, DDRTree clustering indicated that SAB-infected HEFs follow two main trajectories during conversion, both diverging from rtTA-HEFs and, importantly, one leading toward and clustering with primary human fetal astrocytes (Figure 3C). These findings confirm that SAB-infected HEFs is a heterogeneous population consisting of non-converted and partially converted cells but importantly containing one subpopulation that shows similarity to human fetal astrocytes.

Omitting *Nfia* improves efficiency of conversion

Our immunocytochemical and transcriptional analysis showed that conversion could be further optimized. Recently, several reports have pointed toward the importance of nuclear factor 1A (NFIA) in the gliogenic switch but not for astrocyte maturation (Tchieu et al., 2019; Tiwari et al., 2018). To investigate individual contribution of *Sox9*, *Nfia*, and *Nfib* and establish a minimal combination for optimal conversion, we performed a screen where HEFs were infected with single, pairs, or all three transcription factors (Figure S2A). Three weeks after induction, we analyzed the expression of GFAP and S100B by immunocytochemistry. In conditions with *Nfia* or *Nfib* alone we found S100B+ cells to be extremely rare. In contrast, *Sox9* was sufficient to induce S100B expression in some cells. However, we could not detect any GFAP-expressing cells using either *Sox9* or *Nfia*, whereas rare GFAP+ cells were found using *Nfib*. The *Nfia* + *Nfib* condition resulted in some S100B+ but few GFAP+ cells. On the other hand, with *Sox9* + *Nfia* or *Sox9* + *Nfib* we observed more cells expressing S100B and GFAP. Strikingly, *Sox9* + *Nfib*-infected HEFs generated substantially more S100B+ and GFAP+ cells as compared with all three transcription factors. In addition, a larger proportion of cells displayed astrocytic morphologies when using *Sox9* + *Nfib* (Figure S2A). This was even more striking after an additional 2 weeks when F-actin staining, used for better visualization of morphology, revealed an apparent improved astrocytic morphology compared with SAB-HEF-iAs (Figure 4A).

Quantification at 3 weeks confirmed our observation that the *Sox9* + *Nfib* (SB) condition improved conversion with a yield of 255%, 156%, and 96% and purity of 49%, 19%, and 12% S100B+, GFAP+, and S100B+/GFAP+ co-expressing cells, respectively (Figure 4B). This is approximately a 3-fold increase in yield compared with SAB-infected HEFs, while purity remained similar. The increased yield could be explained by better survival or increased proliferation. To assess this, we quantified total number and Ki67+ cells at 1 and 3 weeks. Total cell numbers were higher in SB compared with SAB conditions at 1 week. At 3 weeks, SAB cultures with serum had similar number of cells as SB, but higher than SAB in FGF2 + Serum-free + SM conditions (Figure S2B). Proliferation was overall low, especially at 3 weeks when less than 1% of S100B- and/or GFAP-expressing cells were Ki67+. We could not detect any significant differences between conditions, only a tendency toward higher proliferation in SAB-infected HEFs cultured with serum at 3 weeks (Figures S2C and S2D).

Taken together, these data indicate that SB-infected HEFs have higher survival, but proliferation most likely does not contribute to the increased yield.

Furthermore, using qRT-PCR we observed similar levels of *S100B* in SAB and SB but increased *GFAP* and further decreased expression of *DCN*, supporting downregulation of a fibroblast gene program and upregulation of astrocytic genes when using SB (Figure S2E).

To exclude that conversion induces skeletal muscle or renal epithelia, cells that also express GFAP, we analyzed the expression of MyoD and ANP by immunocytochemistry but never detected any immunoreactive cells (Figure S3).

Furthermore, AQP4 and ALDH1L1, reported as astrocyte-enriched markers (Verkhratsky and Nedergaard, 2018), were expressed in GFAP+ cells in both SAB- and

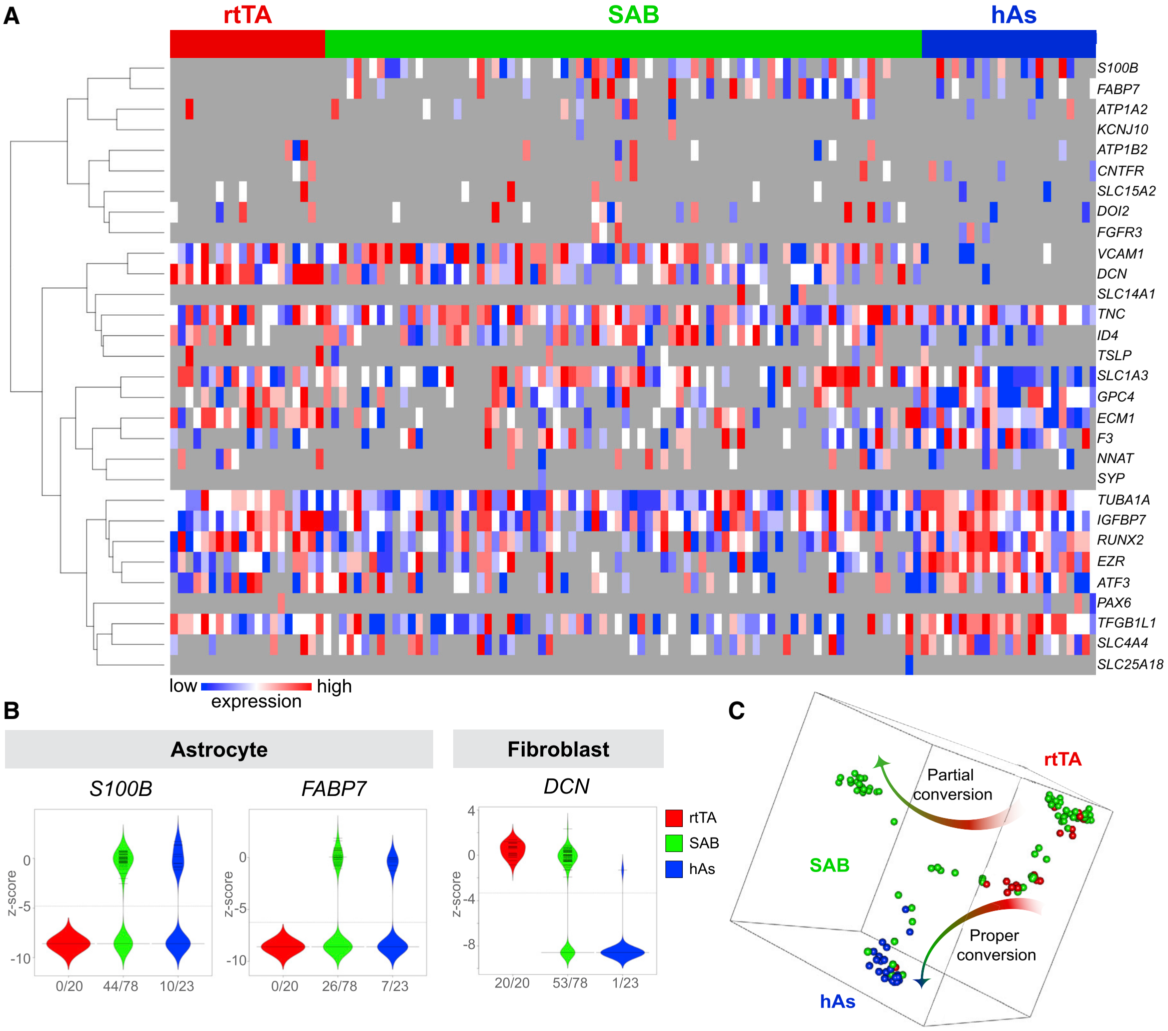

Figure 3. Single-cell gene expression analysis reveals heterogeneity and transcriptional similarities between iAs and human astrocytes

(A) Hierarchical clustering based on expression of astrocyte, fibroblast, and neural lineage genes in rtTA-infected control HEFs (red), SAB-infected HEFs (green), and primary human fetal astrocytes (hAs) (blue).

(B) Up- and downregulated genes during conversion. Data are presented as inverted Ct values *Z*-scored.

(C) DDRTree clustering reveals two trajectories of SAB-infected HEFs emerging from rtTA-HEFs.

Data were collected from n = 4 independent experiments. See also Table S4.

SB-infected HEFs and surprisingly also in control fibroblasts (Figures S4A and S4B), though in line with previous studies (Khan et al., 2018; Mola et al., 2009).

Overall, these findings indicate that; single transcription factors are not sufficient, *Nfia* is not needed, and the optimal transcription factor combination for efficient conversion is *Sox9* and *Nfib*.

Sox9 and *Nfib* are sufficient to induce functional astrocytes

Having established *Sox9* + *Nfib*, hereafter referred to as SB-HEF-iAs, as the best combination, we next assessed functional properties by testing the ability to take up glutamate. Similar to SAB-HEF-iAs, the vast majority of SB-HEF-iAs expressed EAAT1 and EAAT2 as well as GS (Figures 4C–4E). In line with previous studies, EAAT1, EAAT2, and GS immunoreactivity was also observed in controls (Cooper et al., 1998; Soni et al., 1991) (Figures S4C–S4E). We compared levels of these markers in rtTA-HEFs as well as in GFAP$^+$ SAB- and SB-HEF-iAs by measuring fluorescence intensity. We observed no difference between conditions in EAAT1, whereas EAAT2 intensity was higher in SB-HEF-iAs than in SAB-HEF-iAs and GS was higher in both SAB-HEF-iAs and SB-HEF-iAs as compared with rtTA-HEFs (Figure S4F).

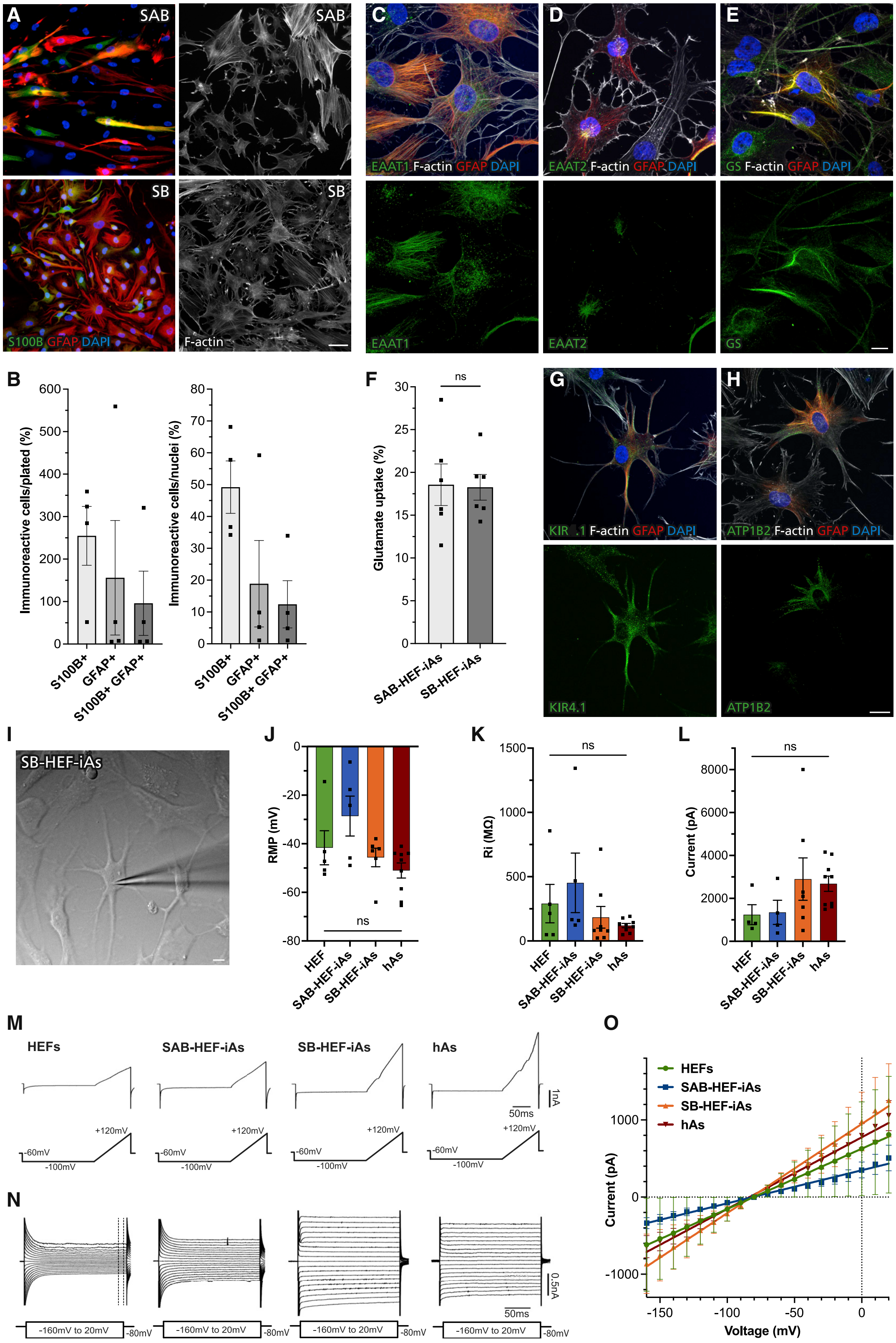

(legend on next page)

Importantly, SB-HEF-iAs had glutamate uptake similar to that of SAB-HEF-iAs, suggesting that they are equally functional (Figure 4F). Furthermore, SB-HEF-iAs also expressed CX43 and spread biocytin to neighboring S100B$^+$ and GFAP$^+$ iAs, suggesting formation of functional gap junctions (Figures S4G and S4H).

Functional syncytia are crucial for homeostatic functions of astrocytes, but expression of ion channels and transporters is also important for buffering. We observed that GFAP$^+$ SAB-HEF-iAs and SB-HEF-iAs expressed higher levels of KIR4.1 than rtTA-HEFs, with SB-HEF-iAs displaying the highest levels (Figures 4G, S5A, and S5B). In addition, ATP1B2 was expressed in 34% of SB-infected cells, and 17% of cells co-expressed ATP1B2 and GFAP, compared with 3% in the SAB condition (Figures 4H, S5C, and S5D).

Expression of KIR4.1 and ATP1B2 suggested that HEF-iAs had machinery to buffer potassium and attain their most critical homeostatic function (Nwaobi et al., 2016). To assess this further, we performed electrophysiological recordings of SAB-HEF-iAs and SB-HEF-iAs at days 37–42 as well as of control HEFs and primary human fetal astrocytes (Figures 4I–4O). First we analyzed passive membrane properties, such as resting membrane potential (RMP) and input resistance (Ri). Interestingly, SB-HEF-iAs had an RMP of −46 mV and Ri of 184 MΩ, similar to human fetal astrocytes, which was −51 mV and 120 MΩ, respectively. This is in line with previous reports from *in vitro* stem cell-derived and fetal human astrocytes (Neyrinck et al., 2021). On the other hand, SAB-HEF-iAs had lower hyperpolarized RMP of −28 mV and higher Ri of 452 MΩ, and HEFs an RMP of −42 mV and Ri of 291 MΩ (Figures 4J and 4K).

Next, we analyzed whole-cell currents, which in astrocytes predominantly reflect the presence of K$^+$ channels. We executed two protocols in voltage clamp mode. First, a ramp of depolarizing currents was evoked and quantified at the peak where voltage reached +120 mV (Figures 4L and 4M). In all groups, large outward-rectifying currents at potentials higher than −40 mV were observed. Induced currents were bigger, although not significantly so, in SB astrocytes and human primary astrocytes compared with SAB-HEF-iAs and HEFs, respectively. This observation agrees with previous data that primary cultured astroglial cells express large outward-rectifying K$^+$ currents (Bevan and Raff, 1985).

Second, we induced whole-cell currents using a voltage-step protocol ranging from −160 mV to +20 mV at holding potential of −80 mV and used the steady-state current to plot a quasi-linear current/voltage (I/V) curve, characteristic for astrocytes (Figures 4N and 4O). Mature astrocytes have more voltage-gated K$^+$ conductance, leading to membrane hyperpolarization, general high cell conductance, and low membrane resistance, reflected in the linear I/V curve with a steeper slope (Dallérac et al., 2013). Interestingly, SB-HEF-iAs had characteristics closest matching to human astrocytes, although data between groups were variable.

These findings show that both SAB-HEF-iAs and SB-HEF-iAs have electrophysiological features characteristic for astrocytes and indicate that SB-HEF-iAs performs slightly better.

In summary, by removing *Nfia*, significantly more iAs are generated and a larger proportion displays morphologies closer to bona fide astrocytes. Furthermore, SB-HEF-iAs have similar or better astrocyte marker expression and function compared with SAB-HEF-iAs.

SB-HEF-iAs display ATP-induced calcium signaling and are immunocompetent

Astrocytes are known to respond to stimuli by regulating intracellular calcium levels. To assess this, we performed

Figure 4. *Sox9* and *Nfib* improve conversion of human embryonic fibroblasts to induced astrocytes
(A) Representative immunofluorescence images of HEFs 5 weeks after induction of *Sox9*, *Nfia*, and *Nfib* (SAB) or *Sox9* and *Nfib* (SB).
(B) Quantification of yield and purity of S100B, GFAP, and S100B/GFAP immunoreactive cells at 3 weeks.
(C–E) Representative maximum-intensity projection immunofluorescence images of glutamate transporters and GS in SB-HEF-iAs at 5 weeks.
(F) Glutamate uptake in SAB-HEF-iAs and SB-HEF-iAs at 5 weeks.
(G and H) Representative maximum-intensity projection immunofluorescence images of KIR4.1 and ATP1B2 in SB-HEF-iAs at 5 weeks.
(I) Example image of whole-cell patch-clamp recording of SB-HEF-iAs with patch pipette indicating a recorded cell.
(J and K) Resting membrane potential (RMP) and input resistance (Ri) values.
(L) Values of maximum current induced by ramp depolarization protocol.
(M) Current trace examples induced by ramp depolarization.
(N) Examples of current traces induced by voltage steps ranging from −160 to +20 mV. Dotted lines indicate portion of the trace where steady-state currents were quantified.
(O) Current/voltage (I/V) curve typical for astrocytes built from data obtained in (N).
hAs, primary human fetal astrocytes. Individual color levels are adjusted for visibility in (A). Data are presented as mean ± SEM of n = 2–6 independent experiments. Data points in (J), (K), and (L) represent individual cells. Wilcoxon matched-pairs rank test (F), Kruskal-Wallis test (J, K, L), and multiple unpaired t tests (O) with significance level $p < 0.05$ were performed for statistical analysis. Scale bars, 50 μm (A) and 10 μm (C, D, E, G, H, and I). See also Figures S2, S4, and S5.

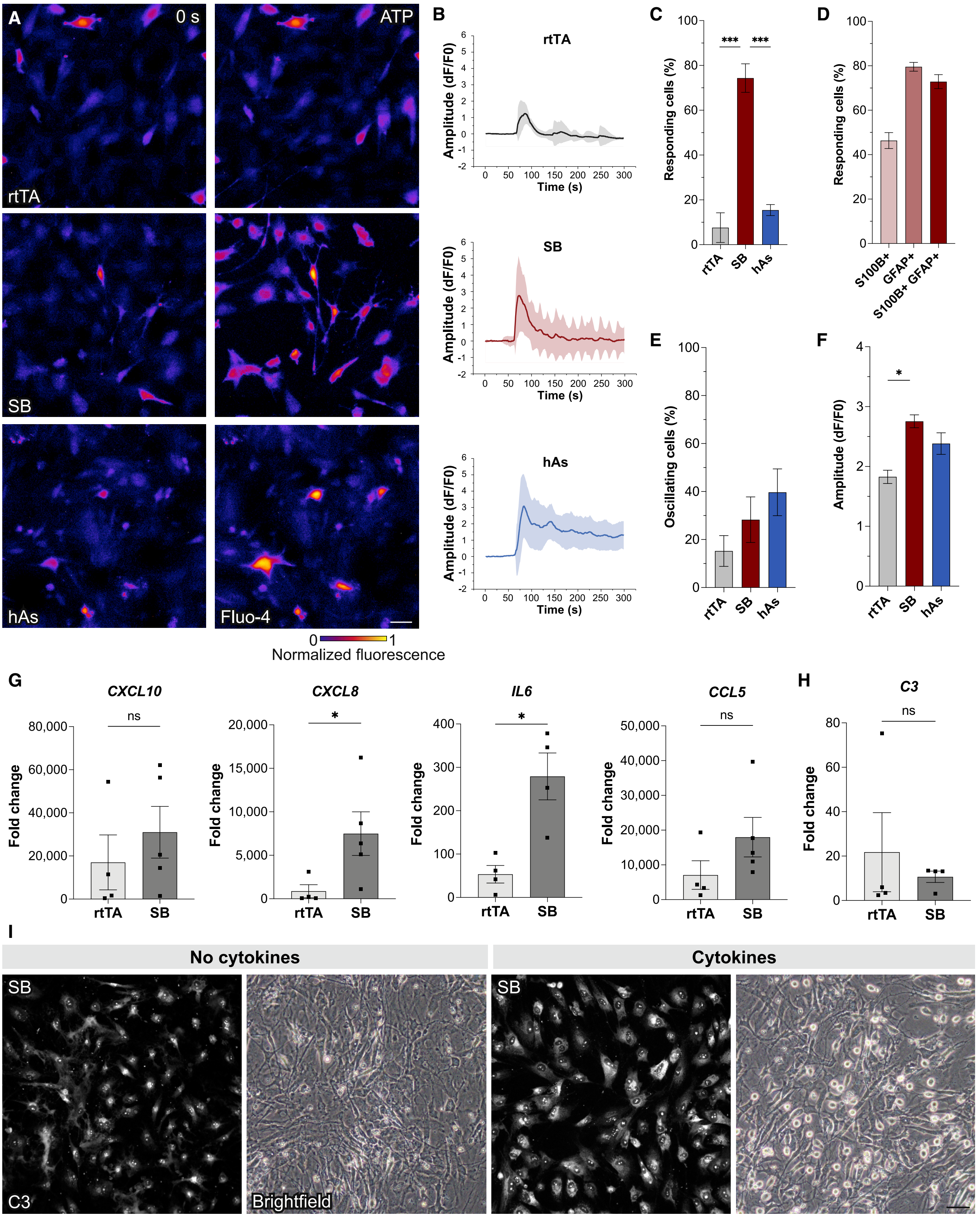

(legend on next page)

calcium imaging experiments with Fluo4-loaded SB-HEF-iAs at days 37–38. We observed a clear response to ATP in SB-HEF-iAs with 74% of cells having ATP-induced calcium waves that was distinct from rtTA-HEFs and even human fetal astrocytes, in which only 7.6% and 15% of cells responded, respectively (Figures 5A–5C; Videos S1, S2, and S3). Importantly, post hoc identification revealed that 46%, 80%, and 73% of $S100B^+$, $GFAP^+$, and $S100B^+$/$GFAP^+$ co-expressing SB-HEF-iAs, respectively, responded to ATP, indicating that the majority of $GFAP^+$ cells display ATP-induced calcium waves (Figure 5D). Of the responding cells, slightly more SB-HEF-iAs showed oscillations, though not significantly more than rtTA-HEFs (Figure 5E). However, the amplitude of evoked responses was higher in SB-HEF-iAs (Figure 5F). This suggests that SB-HEF-iAs acquired intercellular calcium signaling similar to and even more prominent than primary fetal astrocytes that is distinct from control HEFs.

Next, we assessed immunological functionality, since astrocytes participate in regulation of CNS inflammation (Sofroniew and Vinters, 2010). We stimulated SB-HEF-iAs for 24 h with C1q, tumor necrosis factor α (TNFα) and interleukin-1α (IL-1α), a cocktail shown to activate mouse and human pluripotent stem cell (hPSC)-derived astrocytes (Barbar et al., 2020; Liddelow et al., 2017), and analyzed gene and protein expression. Strikingly, SB-HEF-iAs upregulated cytokines *CXCL10*, *CXCL8*, *IL-6*, and *CCL5*, and immune-related complement factor *C3*. Upregulation of *CXCL8* and *IL-6* was significantly higher in SB-HEF-iAs as compared with rtTA-HEFs (Figure 5G). We also observed downregulation of *MKI67* and *GFAP* and upregulation of *VIM* and *CD44* in cytokine-treated SB-HEF-iAs (Figure S6A), in line with previous studies on hPSC-derived astrocytes (Barbar et al., 2020). In addition we observed that SB-HEF-iAs had higher levels of C3D protein accompanied by rounding of the soma (Figures 5H and 5I), similar to what was described for hPSC-derived astrocytes (Tchieu et al., 2019). These data suggest that SB-HEF-iAs are immunocompetent.

Taken together, SB-HEF-iAs acquired astrocytic functional properties that are distinct from control HEFs.

To assess the importance of reprogramming factor expression during conversion, we analyzed SOX9 using immunocytochemistry in SB-infected HEFs. We found no difference in the number of $SOX9^+$ cells between converted and non-converted cells at 1 and 3 weeks. However, intensity of SOX9 staining was higher in converted than in non-converted cells at 1 week. Surprisingly, at 3 weeks there was slightly higher intensity in non-converted cells (Figures S6B and S6C), suggesting that initial high levels of transgenes are important although other factors such as repressive mechanisms might also control conversion.

Finally, in an attempt to infer subtype identity to iAs, we performed qRT-PCR for markers of different developing CNS regions in SB-HEF-iAs at 5 weeks as well as human cortical fetal astrocytes. SB-HEF-iAs expressed mainly markers for hindbrain (*MYBPC1* and *WIF1*) and spinal cord (*HOXB4*, *HOXC8*, and *HOXC6*) but also some forebrain markers (*OTX2*, *DMRT2A*, *CHRDL1*, and *CRYM*) (Figure S6D) while fetal cortical astrocytes expressed forebrain and ventral markers (*NKX2.1*). These results could indicate that SB-HEF-iAs have a more hindbrain/spinal cord identity compared with human cortical fetal astrocytes.

Conversion of postnatal, adult, and aged human fibroblasts to astrocytes

To enable modeling of neurological disorders using patient fibroblasts and utilizing advantages of direct conversion such as preservation of genetic heterogeneity and age- and disease-related features, successful conversion of postnatal human fibroblasts is essential. Therefore, we applied our SB protocol to neonatal and adult human fibroblasts. Indeed, 3 weeks after induction, we observed $S100B^+$ and $GFAP^+$ cells by immunostaining. Importantly, strengthening our previous findings, substantially more $S100B^+$

Figure 5. SB-HEF-iAs display ATP-induced calcium response and are immunocompetent

(A) Background subtracted images of live-cell calcium imaging experiments on Fluo-4-loaded rtTA-HEFs and SB-HEF-iAs at 5 weeks, and primary human fetal astrocytes (hAs).

(B) Mean relative fluorescence changes of calcium traces of ATP-responding cells normalized to baseline (dF/F0).

(C) Percentage of ATP-responding cells using a threshold of 3 × SD of baseline.

(D) Percentage of ATP-responding cells of S100B, GFAP, and S100B/GFAP co-expressing SB-HEF-iAs.

(E) Percentage of oscillating cells of ATP-responding cells.

(F) Amplitude of ATP-evoked response in responding cells.

(G and H) qRT-PCR analysis upon stimulation with TNFα, IL-1α, and C1q. Data are presented as fold change relative to non-stimulated controls.

(I) Representative immunostaining and bright-field image of SB-HEF-iAs following treatment with TNFα, IL-1α, and C1q.

Data are presented as mean ± SEM, except for calcium traces (B) presented as mean ± SD, of n = 3–4 independent experiments. Kruskal-Wallis test (C–F) and Mann-Whitney test (G and H) were performed for statistical analysis. $*p < 0.05$, $***p < 0.001$. Scale bars, 50 μm (A and I). See also Figure S6 and Videos S1–S3.

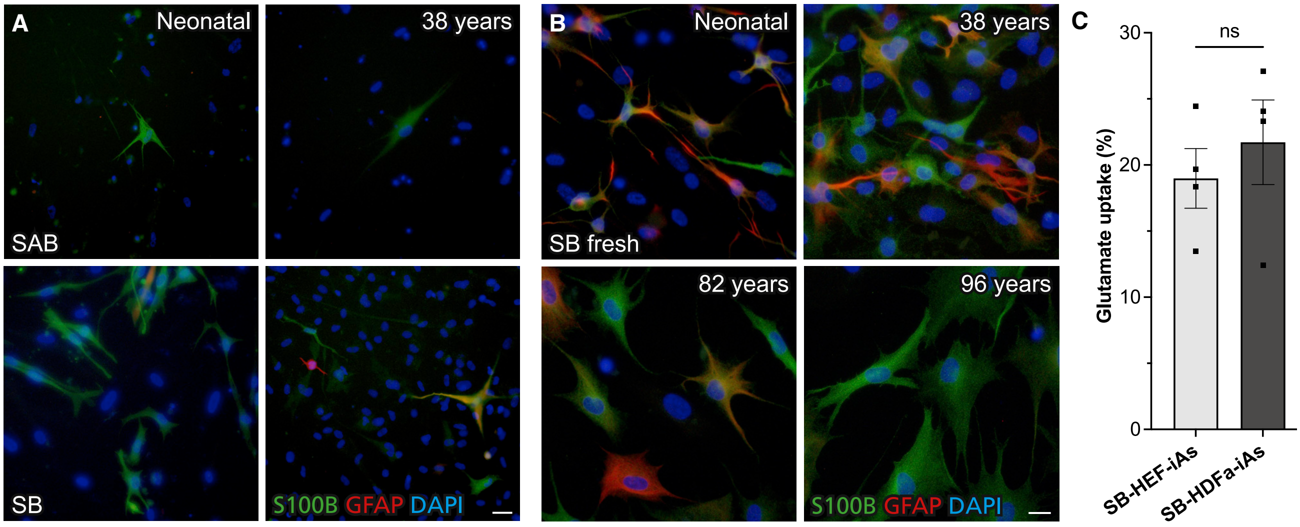

Figure 6. Direct conversion of human neonatal, adult, and aged fibroblasts to induced astrocytes
(A and B) Immunofluorescence images of human neonatal and adult fibroblasts (38–96 years) 3 weeks after induction of SAB or SB (A) or freshly prepared SB (B).
(C) Glutamate uptake of SB-infected HEFs and human adult fibroblasts (38 years) at 5 weeks.
Individual color levels are adjusted for visibility in (A) and (B). Data are presented as mean ± SEM of n = 4 parallel independent experiments. Two-tailed Wilcoxon matched-pairs rank test with significance level $p < 0.05$ was performed for statistical analysis in (C). Scale bars, 50 μm (A) and 20 μm (B).

and GFAP^{+} cells were observed by overexpressing SB as compared with SAB (Figure 6A).

Although we could obtain iAs from postnatal cells, conversion was not as efficient as in HEFs, which is in line with previous reports on direct conversion (Tian et al., 2016). We speculated that this could be due to adult fibroblasts requiring higher titer virus and increased transgene expression. We therefore tested freshly prepared, not previously frozen, viral preparations. Strikingly, this improved the conversion of neonatal as well as adult fibroblasts and allowed for conversion of aged fibroblasts (Figure 6B). In line with previous reports (Gatto et al., 2020), we observed that iAs obtained from adults were larger compared to neonatal iAs. To assess the functionality of iAs obtained from adult fibroblasts, we analyzed glutamate uptake and found that adult iAs took up glutamate at levels similar to those of as HEF-iAs (Figure 6C).

These findings indicate that removal of *Nfia* improved direct conversion of neonatal and adult fibroblasts to iAs. Importantly, this also allowed for conversion of fibroblasts derived throughout the human lifespan.

Induced astrocytes support the function of induced neurons in co-cultures derived from the same starting fibroblast population

Rodent astrocytes are frequently used to support PSC-derived as well as directly converted neurons in co-culture. However, given the vast difference between rodent and human astrocytes, it would be desirable to directly convert neurons and astrocytes from the same starting population. Such co-cultures would enable disease modeling and studies of human neuron-astrocyte interactions. To establish this, we generated induced neurons (iNs), through direct conversion of HEFs using *Ascl1* and *Ngn2* overexpression (Ladewig et al., 2012), and cultured them alone or together with SB-HEF-iAs.

iNs expressed neuronal markers βIII-TUBULIN and MAP2 (Figure 7A), and we observed that when iNs were cultured alone, cells started to detach between days 33 and 47 in approximately 50% of coverslips. In contrast, when co-cultured with SB-HEF-iAs, cells detached only in around 4% of coverslips (Figures 7B and S7).

To test whether iAs influence the functional properties of neurons, we performed whole-cell patch-clamp recordings from iNs cultured alone or in co-culture with iAs at days 45–52 (Figures 7C–7I). We divided iNs by the ability to fire action potentials (APs). All recorded cells fired at least a single AP, but strikingly iNs in co-cultures had a higher proportion of cells able to repetitively and sustainably fire higher numbers (>3) of APs in response to increasing depolarizing current steps (41% in co-cultures versus 23% in iNs alone) (Figures 7C and 7D). The proportion of spontaneously active cells at RMP was also higher in the co-culture group (29% compared with 6%) (Figures 7E and 7F). We observed that AP characteristics such as amplitude and half-width

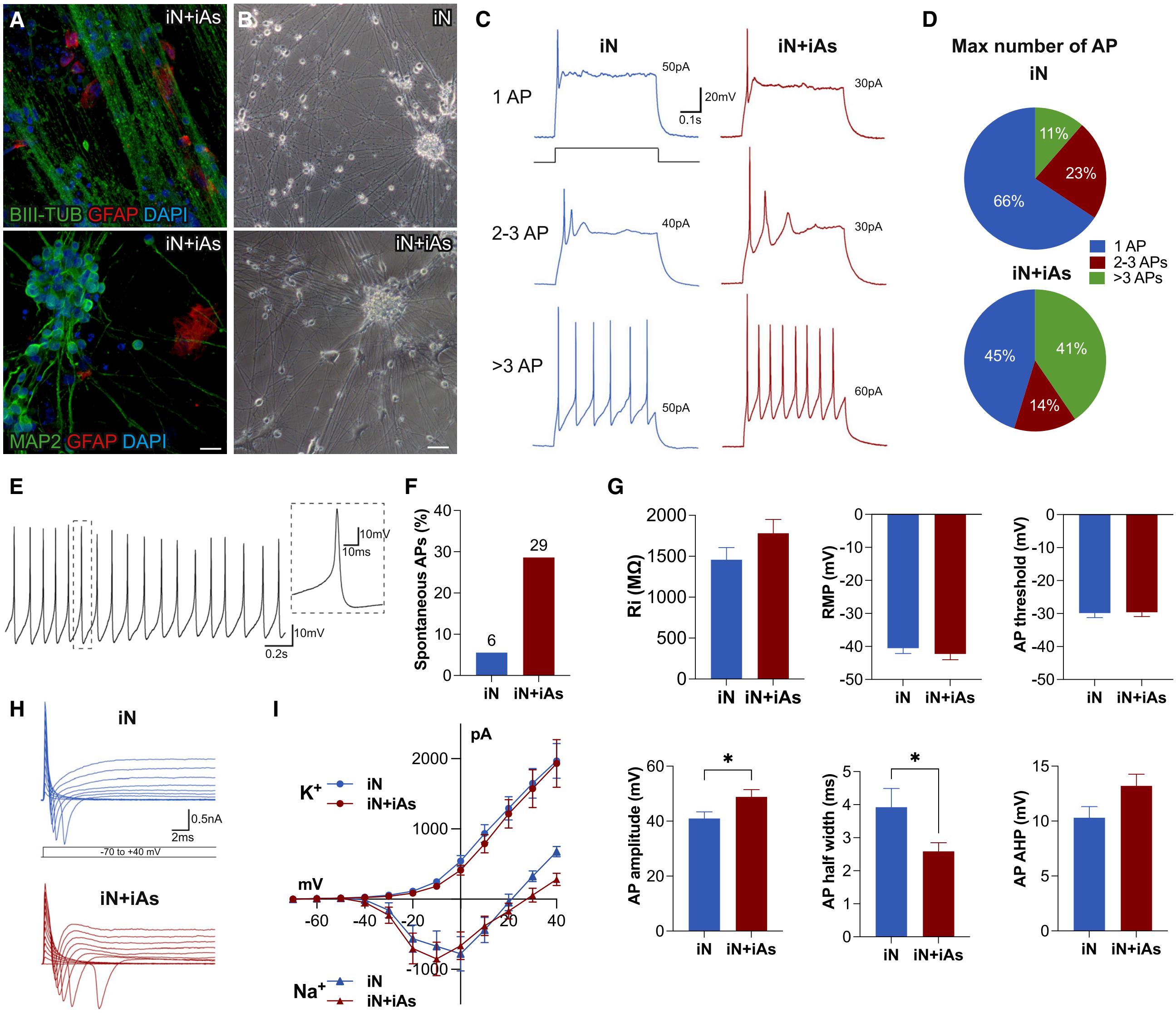

Figure 7. Improved functionality of induced neurons in co-cultures with iAs

(A) Maximum-intensity projection immunofluorescence images of iNs in co-culture with SB-HEF-iAs (iN + iAs) at 6–8 weeks.

(B) Bright-field images of iN alone and iN + iAs co-cultures.

(C) Example traces of whole-cell current clamp recordings of iNs with three different patterns of action potential (AP) firing. APs are induced by depolarization current step at membrane potential of −70 mV. Current injected in each trace is indicated.

(D) iNs grouped based on number of APs fired.

(E) Example trace of recorded iNs with spontaneous APs at resting membrane potential (RMP). Inset shows magnified view of a single AP.

(F) Percentage of iNs with spontaneous APs at RMP.

(G) Intrinsic membrane properties and AP parameters of iNs.

(H) Example traces of inward Na^+ and outward K^+ whole-cell currents evoked by voltage steps ranging from −70 to +40 mV in voltage clamp mode from holding potential of −70 mV.

(I) Outward K^+ and inward Na^+ peak currents plotted against voltage steps shown in (H).

Individual color levels are adjusted for visibility in (A). Data are presented as mean ± SEM of n = 38–40 cells from three independent experiments. Unpaired t test was performed for statistical analysis in (G). *$p < 0.05$. Scale bars, 20 μm (A) and 50 μm (B). See also Figure S7.

were significantly different and closer to more mature neurons with higher AP amplitude when cultured with iAs (Figure 7G). Other intrinsic parameters (Ri and RMP) and AP characteristics (AP threshold and afterhyperpolarization) were not statistically different between groups (Figure 7G).

Another key property of functional neurons is the presence of active voltage-gated Na^+ and K^+ channels. Both fast inward Na^+ and slow outward K^+ currents could be induced by stepping voltage from −70 to +40 mV with 10 mV increments in voltage clamp configuration (Figure 7H); however, we did not observe any difference between groups in I/V curves obtained by quantification of these currents (Figure 7I). We did not observe any clear spontaneous postsynaptic currents in either iN alone or in co-cultures, indicating that a longer time is needed to reach full neuronal maturation and connectivity.

In conclusion, we present for the first time a co-culture system of functional human neurons and astrocytes obtained through direct conversion from the same starting fibroblast population, in which iAs support the maturation of iNs. This co-culture system has promising potential for disease modeling and the study of astrocyte-neuron interactions.

DISCUSSION

Here we describe effective generation of iAs from human fibroblasts, derived throughout the lifespan, using transcription factor-mediated direct conversion. iAs resembled primary fetal human astrocytes at a molecular, phenotypical, and functional level. We also, for the first time, describe a functional co-culture system with iAs and iNs obtained through direct conversion from the same starting human fibroblast population.

There are several methods to generate astrocytes from human embryonic stem cells (hESCs) and induced PSCs (iPSCs) (Canals et al., 2018; Mertens et al., 2016). However, methods to directly convert fibroblasts to astrocytes, such as the one described here, have only been briefly explored but have important advantages. Besides being faster, direct conversion preserves genetic mosaicism present in starting fibroblast populations, whereas iPSCs are clonal (Abyzov et al., 2012; Mertens et al., 2016). If the level of mosaicism is similar in astrocytes and fibroblasts, which is likely, directly converted cells would be better at recapitulating genetic heterogeneity. Furthermore, age-related epigenetic properties are erased during iPSC reprogramming and yield rejuvenated cells. Direct conversion studies on iNs and induced oligodendrocytes, on the other hand, have been shown to generate cells that retain aging phenotypes (Chanoumidou et al., 2021; Mertens et al., 2015). Thus, for modeling of sporadic neurodegenerative disorders, in which age is the most significant risk factor, direct conversion constitutes an attractive approach for generation of diseased cells.

The potency of *Sox9*, *Nfia*, and *Nfib* inducing astrocytic cell fate is in accordance with studies performed in developing embryonic chick spinal cord, showing that these transcription factors control the initiation of gliogenesis (Molofsky et al., 2012). However, we discovered that the yield of converted cells was higher when *Sox9* and *Nfib* (SB) were overexpressed. Supporting this finding, it was recently shown that transient overexpression of *NFIA* in hESC-derived neural stem cells yields more astrocytes compared with continuous overexpression, indicating that *NFIA* is important for initial specification of astrocytes but needs to be downregulated for maturation (Tchieu et al., 2019). This deviates from what was reported for direct conversion of mouse fibroblasts whereby SAB was more efficient than SB (Caiazzo et al., 2015). However, it is in line with our recent finding that SB efficiently drives astrocytic induction in hPSCs (Canals et al., 2018). We cannot exclude that sequential expression of first *Nfia* and then *Sox9* and *Nfib* would yield results similar to those described here but would require a more complex expression system.

Our approach improved the yield of directly converted astrocytes by over 40- and 120-fold using SAB or SB, respectively, compared with a previous study (Caiazzo et al., 2015). The starting fibroblast population could in part explain the increased efficiency. We opted to use embryonic fibroblasts to optimize conversion while Caiazzo et al. used neonatal fibroblasts. Indeed, conversion of postnatal fibroblasts was shown to be less efficient, possibly due to reduced cell plasticity, proliferation, and onset of senescence in older fibroblasts (Ahlenius et al., 2016). However, we also successfully converted adult and aging fibroblasts. Therefore, another plausible explanation is that by using selectable vectors we obtain a higher number of transduced cells and increased level of transgene expression, which is supported by our finding that freshly prepared viruses was crucial to conversion of postnatal fibroblasts. However, we also observed unconverted cells expressing at least one transgene, indicating that other mechanisms, such as repressive factors, might be involved in controlling conversion, as reported for neuronal conversion (Drouin-Ouellet et al., 2017).

Several lines of evidence, including morphological observations, immunocytochemical analysis, and gene expression profiling, support our interpretation that iAs are of astrocytic lineage. Importantly, we extensively characterized known functional properties of astrocytes in iAs and demonstrated that they take up glutamate, form functional gap junctions, are immunocompetent, display regulated calcium signaling, have appropriate electrophysiological properties, and support neuronal functionality

(Sofroniew and Vinters, 2010). Interestingly, iAs expressed several markers reported in mature astrocytes (Lattke et al., 2021). However, since comparison with bona fide adult human astrocytes *in vivo* is not possible, it is difficult to judge exactly how mature iAs are, although we conclude that they are at least as mature as primary human fetal astrocytes.

Our finding that functional astrocytes can be derived directly from human fibroblasts is supported by a previous study that used chemical reprogramming to generate iAs from human foreskin fibroblasts (Tian et al., 2016). Chemical iAs were obtained at similar purity, could take up glutamate, and displayed induced calcium response. However, our method requires less time to generate astrocytes with additional functional properties such as gap-junction coupling, electrophysiological properties, and immunocompetence, and, importantly, iAs derived from all ages display morphology more closely resembling that of bona fide human astrocytes. In addition, the majority of established direct conversion methods are based on transcription factors, which makes it relatively easy to produce a variety of neural and non-neural cells without changing methodology and, as we show here, combine into useful co-cultures.

Furthermore, single-cell gene expression analysis of SAB-HEF-iAs allowed us to assess heterogeneity during conversion and revealed that the process yields a complex mixture of cells, which is not ideal for certain purposes. However, we identified three genes, *S100B*, *FABP7*, and *DCN*, which clearly separated control fibroblasts from astrocytes. Based on these genes, we identified a population that clustered closer to human primary fetal astrocytes than other populations.

SB-HEF-iAs expressed mainly hindbrain and spinal cord markers but also forebrain markers. These findings might suggest a more posterior identity, but perhaps a more plausible explanation is that iAs represent a generic astrocyte type.

Finally, we showed that iAs can be co-cultured with iNs obtained through direct conversion from the same starting fibroblasts and that iAs supported functionality of iNs. Similar co-culture systems based on hPSCs have been used to identify cell-autonomous and non-autonomous disease mechanisms and study astrocyte-neuron interactions, for instance in amyotrophic lateral sclerosis (Birger et al., 2019).

In conclusion, we have shown that overexpression of *Sox9* and *Nfib* in human fibroblasts effectively generates iAs with molecular and functional phenotypes similar to primary human astrocytes. Fibroblast-derived iAs, and in combination with iNs ultimately from patients with neurological diseases, have potential to become informative models to elucidate the role of astrocytes in disease, especially for studies on sporadic and age-related neurological disorders.

EXPERIMENTAL PROCEDURES

Human tissue

Human tissue was obtained from dead aborted human fetuses 6–9 weeks post conception according to guidelines approved by the Lund-Malmö Ethical Committee. Human dermal neonatal (C-004-5C) and adult (C-013-5C) fibroblasts were bought from Invitrogen. Aged human fibroblast from an 82-year-old woman (GM01706) and a 96-year-old man (GM00731) were obtained from Coriell biorepository.

Direct conversion

Fibroblasts were seeded on day −2 and transduced on day −1 with rtTA, tetO-Sox9-puromycin, tetO-Nfib-hygromycin, and tetO-Nfia-blasticidin, either alone or in combinations, in Fibroblast medium (Dulbecco's modified Eagle's medium [DMEM], 10% fetal bovine serum [FBS], 1% GlutaMAX, 1% non-essential amino acid [NEAA], and 1% sodium pyruvate, all from Gibco) containing 8 μg/mL Polybrene (Sigma). Transgene expression was induced by addition of 2.5 μg/mL doxycycline (Sigma-Aldrich) at day 0 and kept throughout experiments. On day 1, a 3-day puromycin (Gibco) and a 5-day blasticidin (Gibco) and hygromycin (Gibco) selection was started during which medium was changed daily. For FGF2 + Serum-free + SM condition, medium was changed on day 3 to 3:4 Fibroblast medium/FGF medium (Neurobasal, B-27, 1% NEAA, 1% GlutaMAX, 0.2% FBS, 8 ng/mL FGF [Peprotech], 5 ng/mL CNTF [Peprotech], and 10 ng/mL BMP4 [Peprotech]). At day 4 the medium was changed to 1:1 Fibroblast medium/FGF medium and at day 6 1:4 Fibroblast medium/FGF medium. At day 7 the medium was changed to FGF medium and at day 9 half of the medium was changed. At day 11, half of the medium was changed to Serum-free medium (1:1 DMEM-F12, HEPES:Neurobasal, 1% N-2, 1% GlutaMAX, 1% sodium pyruvate, 5 μg/mL *N*-acetyl-L-cysteine [Sigma-Aldrich], and 5 μg/mL heparin-binding EGF-like growth factor [Sigma-Aldrich]) supplemented with 10 ng/mL CNTF, 10 ng/μL BMP4, and 500 μg/mL dibutyryl cAMP (Sigma-Aldrich). Thereafter, half of the medium was changed every second or third day. If needed, cells were split 1:3.

Statistical analysis

Data are presented as mean ± SEM of three to six independent viral transduction experiments performed on separate days (n) unless otherwise stated.

See supplemental experimental procedures for further details.

SUPPLEMENTAL INFORMATION

Supplemental information can be found online at https://doi.org/10.1016/j.stemcr.2022.05.015.

AUTHOR CONTRIBUTIONS

E.Q. was responsible for conceptualization, methodology, validation, investigation, visualization, formal analysis, funding

acquisition, and preparation of the manuscript. F.T. performed calcium imaging and data analysis, and assisted in preparation of the manuscript. N.A., A.G.R., and M.G.H. performed electrophysiological recordings and data analysis, and assisted in preparation of the manuscript. O.G.Z. performed data analysis and assisted in preparation of the manuscript. M.K. supervised A.G.R. I.C. was responsible for methodology and supervision. H.A. was responsible for conceptualization, methodology, supervision, project administration, manuscript writing, and funding acquisition.

ACKNOWLEDGMENTS

We thank Lund Stem Cell Center Imaging, Fluidigm, Bioinformatics, and FACS core facilities for expert assistance, and E. Monni and Z. Kokaia for providing human fetal fibroblasts and astrocytes; M. Eldeeb, I. Hidalgo, and M. Jassinskaja for FACS for single-cell gene expression analysis; M. Mall and J. Kaspar for providing tetO-Ngn2-hygromycin plasmid; and members of Stem Cells, Aging and Neurodegeneration and Stem Cell and Restorative Neurology groups for reagent sharing and feedback. This work was supported by grants from Sävstaholm and Hardebo Foundations to E.Q., as well as the Swedish Research Council, Swedish Brain Foundation, Petrus and Augusta Hedlund, and Crafoord Foundations to H.A. Graphical abstract was created with BioRender.com.

CONFLICT OF INTERESTS

E.Q. is now an employee at AstraZeneca. E.Q. and H.A. have filed a patent application based on findings described in this study.

Received: September 24, 2021
Revised: May 20, 2022
Accepted: May 23, 2022
Published: June 23, 2022

REFERENCES

Abyzov, A., Mariani, J., Palejev, D., Zhang, Y., Haney, M.S., Tomasini, L., Ferrandino, A.F., Rosenberg Belmaker, L.A., Szekely, A., Wilson, M., et al. (2012). Somatic copy number mosaicism in human skin revealed by induced pluripotent stem cells. Nature *492*, 438–442. https://doi.org/10.1038/nature11629.

Ahlenius, H., Chanda, S., Webb, A.E., Yousif, I., Karmazin, J., Prusiner, S.B., Brunet, A., Südhof, T.C., and Wernig, M. (2016). FoxO3 regulates neuronal reprogramming of cells from postnatal and aging mice. Proc. Natl. Acad. Sci. U S A *113*, 8514–8519. https://doi.org/10.1073/pnas.1607079113.

Allen, N.J., and Eroglu, C. (2017). Cell biology of astrocyte-synapse interactions. Neuron *96*, 697–708. https://doi.org/10.1016/j.neuron.2017.09.056.

Barbar, L., Jain, T., Zimmer, M., Kruglikov, I., Sadick, J.S., Wang, M., Kalpana, K., Rose, I.V.L., Burstein, S.R., Rusielewicz, T., et al. (2020). CD49f is a novel marker of functional and reactive human iPSC-derived astrocytes. Neuron *107*, 436–453.e12. https://doi.org/10.1016/j.neuron.2020.05.014.

Bevan, S., and Raff, M. (1985). Voltage-dependent potassium currents in cultured astrocytes. Nature *315*, 229–232. https://doi.org/10.1038/315229a0.

Birger, A., Ben-Dor, I., Ottolenghi, M., Turetsky, T., Gil, Y., Sweetat, S., Perez, L., Belzer, V., Casden, N., Steiner, D., et al. (2019). Human iPSC-derived astrocytes from ALS patients with mutated C9ORF72 show increased oxidative stress and neurotoxicity. EBioMedicine *50*, 274–289. https://doi.org/10.1016/j.ebiom.2019.11.026.

Caiazzo, M., Giannelli, S., Valente, P., Lignani, G., Carissimo, A., Sessa, A., Colasante, G., Bartolomeo, R., Massimino, L., Ferroni, S., et al. (2015). Direct conversion of fibroblasts into functional astrocytes by defined transcription factors. Stem Cell Rep. *4*, 25–36. https://doi.org/10.1016/j.stemcr.2014.12.002.

Canals, I., Ginisty, A., Quist, E., Timmerman, R., Fritze, J., Miskinyte, G., Monni, E., Hansen, M.G., Hidalgo, I., Bryder, D., et al. (2018). Rapid and efficient induction of functional astrocytes from human pluripotent stem cells. Nat. Methods *15*, 693–696. https://doi.org/10.1038/s41592-018-0103-2.

Chanoumidou, K., Hernández-Rodríguez, B., Windener, F., Thomas, C., Stehling, M., Mozafari, S., Albrecht, S., Ottoboni, L., Antel, J., Kim, K.P., et al. (2021). One-step reprogramming of human fibroblasts into oligodendrocyte-like cells by SOX10, OLIG2, and NKX6.2. Stem Cell Rep. *16*, 771–783. https://doi.org/10.1016/j.stemcr.2021.03.001.

Cooper, B., Chebib, M., Shen, J., King, N.J., Darvey, I.G., Kuchel, P.W., Rothstein, J.D., and Balcar, V.J. (1998). Structural selectivity and molecular nature of L-glutamate transport in cultured human fibroblasts. Arch. Biochem. Biophys. *353*, 356–364. https://doi.org/10.1006/abbi.1998.0626.

Dallérac, G., Chever, O., and Rouach, N. (2013). How do astrocytes shape synaptic transmission? Insights from electrophysiology. Front. Cell. Neurosci. *7*, 159. https://doi.org/10.3389/fncel.2013.00159.

Drouin-Ouellet, J., Lau, S., Brattås, P.L., Rylander Ottosson, D., Pircs, K., Grassi, D.A., Collins, L.M., Vuono, R., Andersson Sjöland, A., Westergren-Thorsson, G., et al. (2017). REST suppression mediates neural conversion of adult human fibroblasts via microRNA-dependent and -independent pathways. EMBO Mol. Med. *9*, 1117–1131. https://doi.org/10.15252/emmm.201607471.

Gatto, N., Dos Santos Souza, C., Shaw, A.C., Bell, S.M., Myszczynska, M.A., Powers, S., Meyer, K., Castelli, L.M., Karyka, E., Mortiboys, H., et al. (2020). Directly converted astrocytes retain the ageing features of the donor fibroblasts and elucidate the astrocytic contribution to human CNS health and disease. Aging Cell *20*, e13281. https://doi.org/10.1111/acel.13281.

Khan, Q.A., Pediaditakis, P., Malakhau, Y., Esmaeilniakooshkghazi, A., Ashkavand, Z., Sereda, V., Krupenko, N.I., and Krupenko, S.A. (2018). CHIP E3 ligase mediates proteasomal degradation of the proliferation regulatory protein ALDH1L1 during the transition of NIH3T3 fibroblasts from G0/G1 to S-phase. PLoS One *13*, e0199699. https://doi.org/10.1371/journal.pone.0199699.

Ladewig, J., Mertens, J., Kesavan, J., Doerr, J., Poppe, D., Glaue, F., Herms, S., Wernet, P., Kögler, G., Müller, F.J., et al. (2012). Small molecules enable highly efficient neuronal conversion of human fibroblasts. Nat. Methods *9*, 575–578. https://doi.org/10.1038/nmeth.1972.

Lattke, M., Goldstone, R., Ellis, J.K., Boeing, S., Jurado-Arjona, J., Marichal, N., MacRae, J.I., Berninger, B., and Guillemot, F. (2021). Extensive transcriptional and chromatin changes underlie

astrocyte maturation in vivo and in culture. Nat. Commun. *12*, 4335. https://doi.org/10.1038/s41467-021-24624-5.

Liddelow, S.A., Guttenplan, K.A., Clarke, L.E., Bennett, F.C., Bohlen, C.J., Schirmer, L., Bennett, M.L., Münch, A.E., Chung, W.-S., Peterson, T.C., et al. (2017). Neurotoxic reactive astrocytes are induced by activated microglia. Nature *541*, 481–487. https://doi.org/10.1038/nature21029.

Mertens, J., Marchetto, M.C., Bardy, C., and Gage, F.H. (2016). Evaluating cell reprogramming, differentiation and conversion technologies in neuroscience. Nat. Rev. Neurosci. *17*, 424–437. https://doi.org/10.1038/nrn.2016.46.

Mertens, J., Paquola, A.C.M., Ku, M., Hatch, E., Böhnke, L., Bohnke, L., Ladjevardi, S., McGrath, S., Campbell, B., Lee, H., et al. (2015). Directly reprogrammed human neurons retain aging-associated transcriptomic signatures and reveal age-related nucleocytoplasmic defects. Cell Stem Cell *17*, 705–718. https://doi.org/10.1016/j.stem.2015.09.001.

Mola, M.G., Nicchia, G.P., Svelto, M., Spray, D.C., and Frigeri, A. (2009). Automated cell-based assay for screening of aquaporin inhibitors. Anal. Chem. *81*, 8219–8229. https://doi.org/10.1021/ac901526k.

Molofsky, A.V., Krenick, R., Ullian, E.M., Tsai, H.H., Deneen, B., Richardson, W.D., Barres, B.A., and Rowitch, D.H. (2012). Astrocytes and disease: a neurodevelopmental perspective. Genes Dev. *26*, 891–907. https://doi.org/10.1101/gad.188326.112.

Neyrinck, K., Van Den Daele, J., Vervliet, T., De Smedt, J., Wierda, K., Nijs, M., Vanbokhoven, T., D'Hondt, A., Planque, M., Fendt, S.-M., et al. (2021). SOX9-induced generation of functional astrocytes supporting neuronal maturation in an all-human system. Stem Cell Rev. Rep. *17*, 1855–1873. https://doi.org/10.1007/s12015-021-10179-x.

Nwaobi, S.E., Cuddapah, V.A., Patterson, K.C., Randolph, A.C., and Olsen, M.L. (2016). The role of glial-specific Kir4.1 in normal and pathological states of the CNS. Acta Neuropathol. *132*, 1–21. https://doi.org/10.1007/s00401-016-1553-1.

Philippeos, C., Telerman, S.B., Oulès, B., Pisco, A.O., Shaw, T.J., Elgueta, R., Lombardi, G., Driskell, R.R., Soldin, M., Lynch, M.D., and Watt, F.M. (2018). Spatial and single-cell transcriptional profiling identifies functionally distinct human dermal fibroblast subpopulations. J. Invest. Dermatol. *138*, 811–825. https://doi.org/10.1016/j.jid.2018.01.016.

Sofroniew, M.V., and Vinters, H.V. (2010). Astrocytes: biology and pathology. Acta Neuropathol. *119*, 7–35. https://doi.org/10.1007/s00401-009-0619-8.

Soni, T., Wolfrom, C., Guerroui, S., Raynaud, N., Poggi, J., Moatti, N., and Gautier, M. (1991). Respective effects of glucose and glutamine on the glutamine synthetase activity of human skin fibroblasts. Mol. Cell. Biochem. *102*, 149–154. https://doi.org/10.1007/BF00234572.

Tchieu, J., Calder, E.L., Guttikonda, S.R., Gutzwiller, E.M., Aromolaran, K.A., Steinbeck, J.A., Goldstein, P.A., and Studer, L. (2019). NFIA is a gliogenic switch enabling rapid derivation of functional human astrocytes from pluripotent stem cells. Nat. Biotechnol. *37*, 267–275. https://doi.org/10.1038/s41587-019-0035-0.

Tian, E., Sun, G., Sun, G., Chao, J., Ye, P., Warden, C., Riggs, A.D., and Shi, Y. (2016). Small-molecule-based lineage reprogramming creates functional astrocytes. Cell Rep. *16*, 781–792. https://doi.org/10.1016/j.celrep.2016.06.042.

Tiwari, N., Pataskar, A., Péron, S., Thakurela, S., Sahu, S.K., Figueres-Oñate, M., Marichal, N., López-Mascaraque, L., Tiwari, V.K., and Berninger, B. (2018). Stage-specific transcription factors drive astrogliogenesis by remodeling gene regulatory landscapes. Cell Stem Cell *23*, 557–571.e8. https://doi.org/10.1016/j.stem.2018.09.008.

Verkhratsky, A., and Nedergaard, M. (2018). Physiology of astroglia. Physiol. Rev. *98*, 239–389. https://doi.org/10.1152/physrev.00042.2016.

Zhang, X.-F., and Cui, X. (2017). Connexin 43: key roles in the skin. Biomed. Rep. *6*, 605–611. https://doi.org/10.3892/br.2017.903.

Zhang, Y., Sloan, S.A., Clarke, L.E., Caneda, C., Plaza, C.A., Blumenthal, P.D., Vogel, H., Steinberg, G.K., Edwards, M.S., Li, G., et al. (2016). Purification and characterization of progenitor and mature human astrocytes reveals transcriptional and functional differences with mouse. Neuron *89*, 37–53. https://doi.org/10.1016/j.neuron.2015.11.013.

Stem Cell Reports, Volume *17*

Supplemental Information

Transcription factor-based direct conversion of human fibroblasts to functional astrocytes

Ella Quist, Francesco Trovato, Natalia Avaliani, Oskar G. Zetterdahl, Ana Gonzalez-Ramos, Marita G. Hansen, Merab Kokaia, Isaac Canals, and Henrik Ahlenius

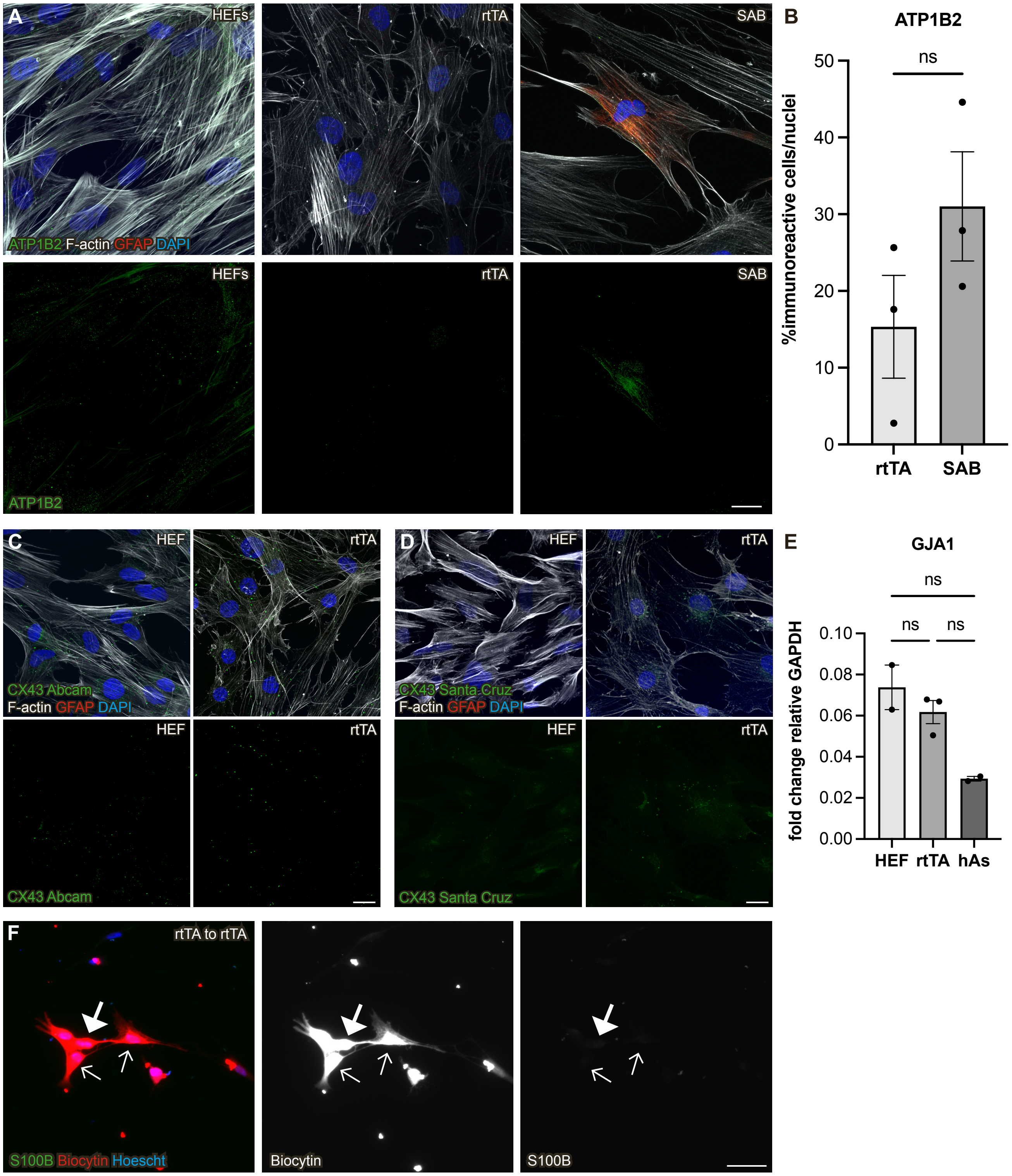

A
HEFs
rtTA
SAB
ATP1B2 F-actin GFAP DAPI
ATP1B2
B
ATP1B2
%immunoreactive cells/nuclei
ns
rtTA
SAB
C
HEF
rtTA
CX43 Abcam
F-actin GFAP DAPI
D
CX43 Santa Cruz
E
GJA1
fold change relative GAPDH
HEF
hAs
F
rtTA to rtTA
S100B Biocytin Hoescht
Biocytin
S100B

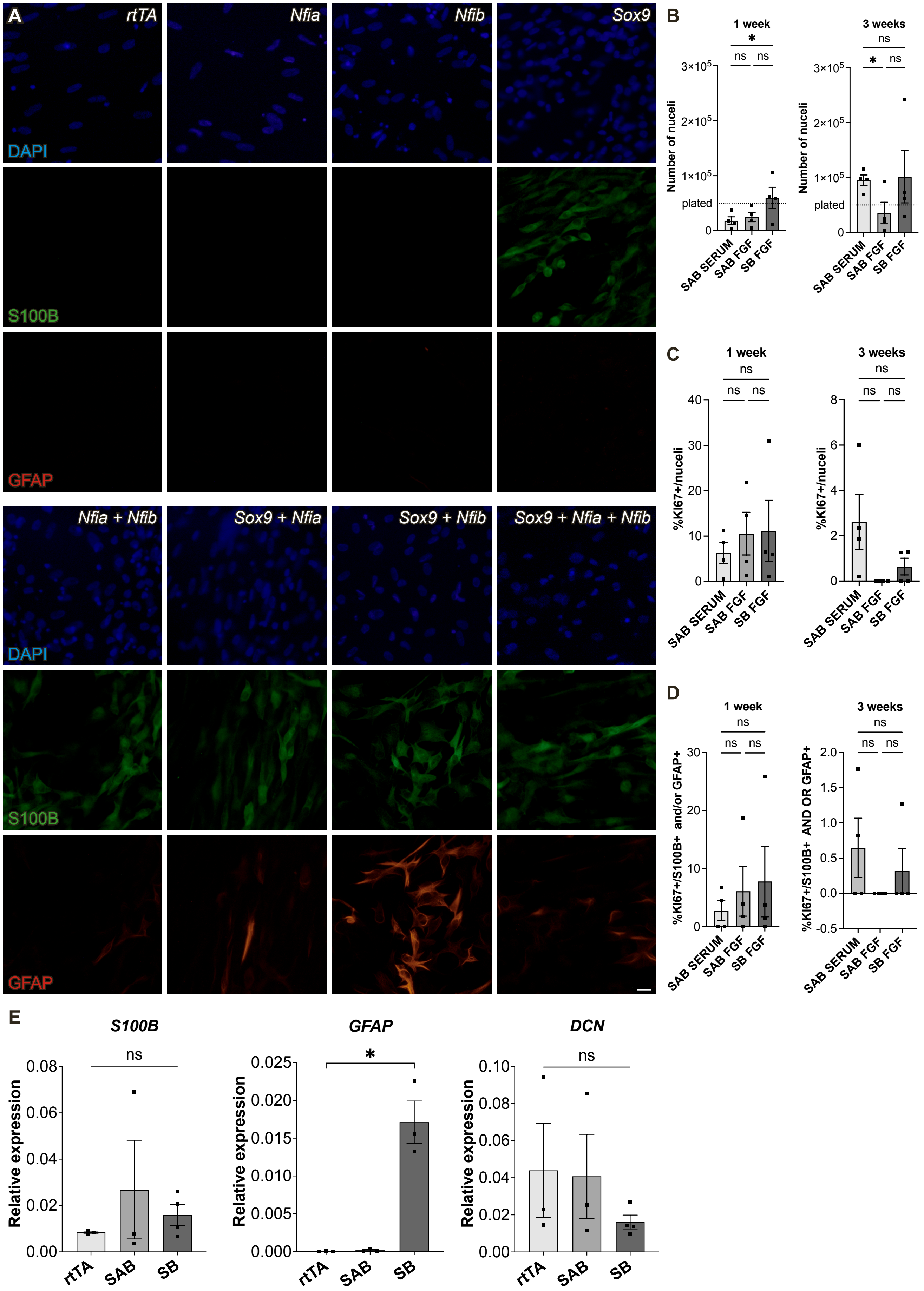

A
rtTA
Nfia
Nfib
Sox9
DAPI
S100B
GFAP
Nfia + Nfib
Sox9 + Nfia
Sox9 + Nfib
Sox9 + Nfia + Nfib
DAPI
S100B
GFAP
B
1 week
3 weeks
Number of nuceli
plated
SAB SERUM
SAB FGF
SB FGF
C
%KI67+/nuceli
D
%KI67+/S100B+ and/or GFAP+
%KI67+/S100B+ AND OR GFAP+
ns
E
S100B
GFAP
DCN
Relative expression
rtTA
SAB
SB

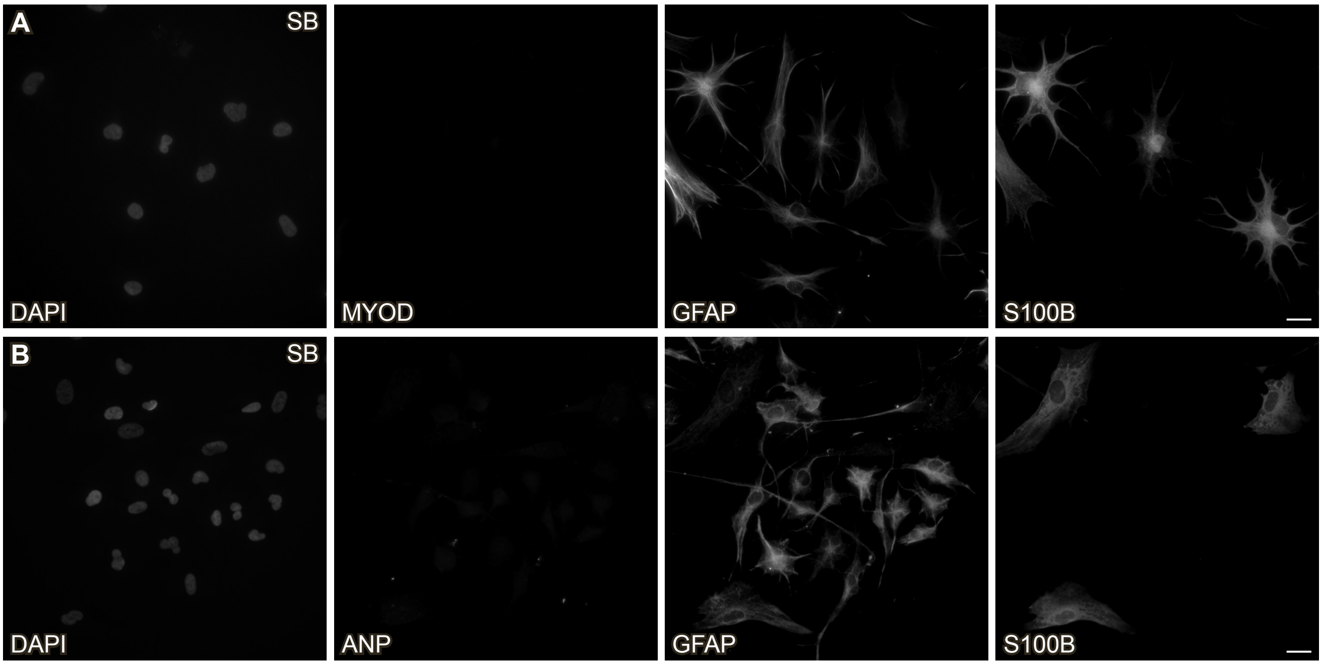
A
SB
DAPI
MYOD
GFAP
S100B
B
SB
DAPI
ANP
GFAP
S100B

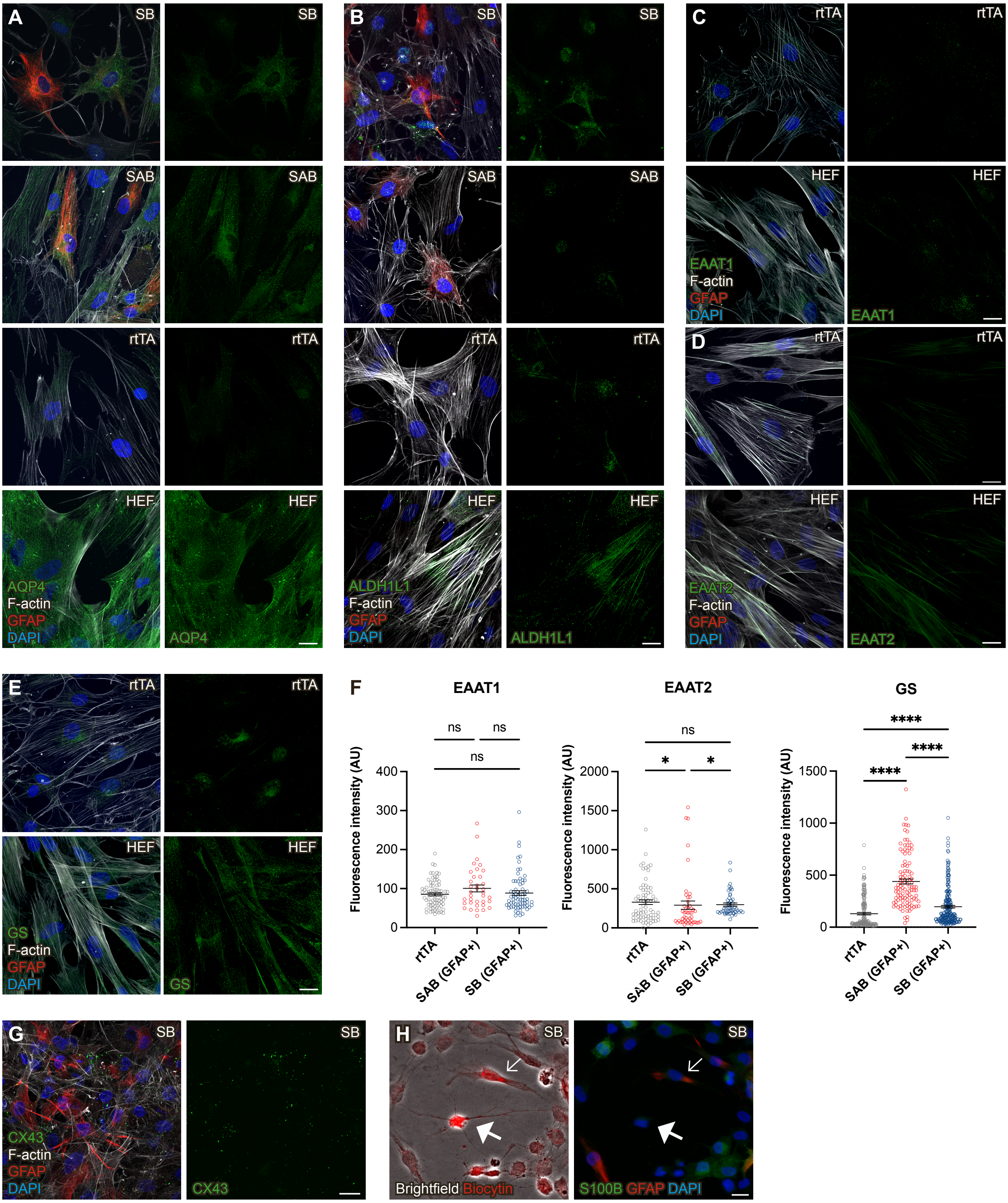

A
SB
SAB
rtTA
HEF
AQP4
F-actin
GFAP
DAPI
B
ALDH1L1
C
EAAT1
D
EAAT2
E
GS
F
EAAT1
EAAT2
Fluorescence intensity (AU)
ns
*
****
rtTA
SAB (GFAP+)
SB (GFAP+)
G
CX43
H
Brightfield Biocytin
S100B GFAP DAPI

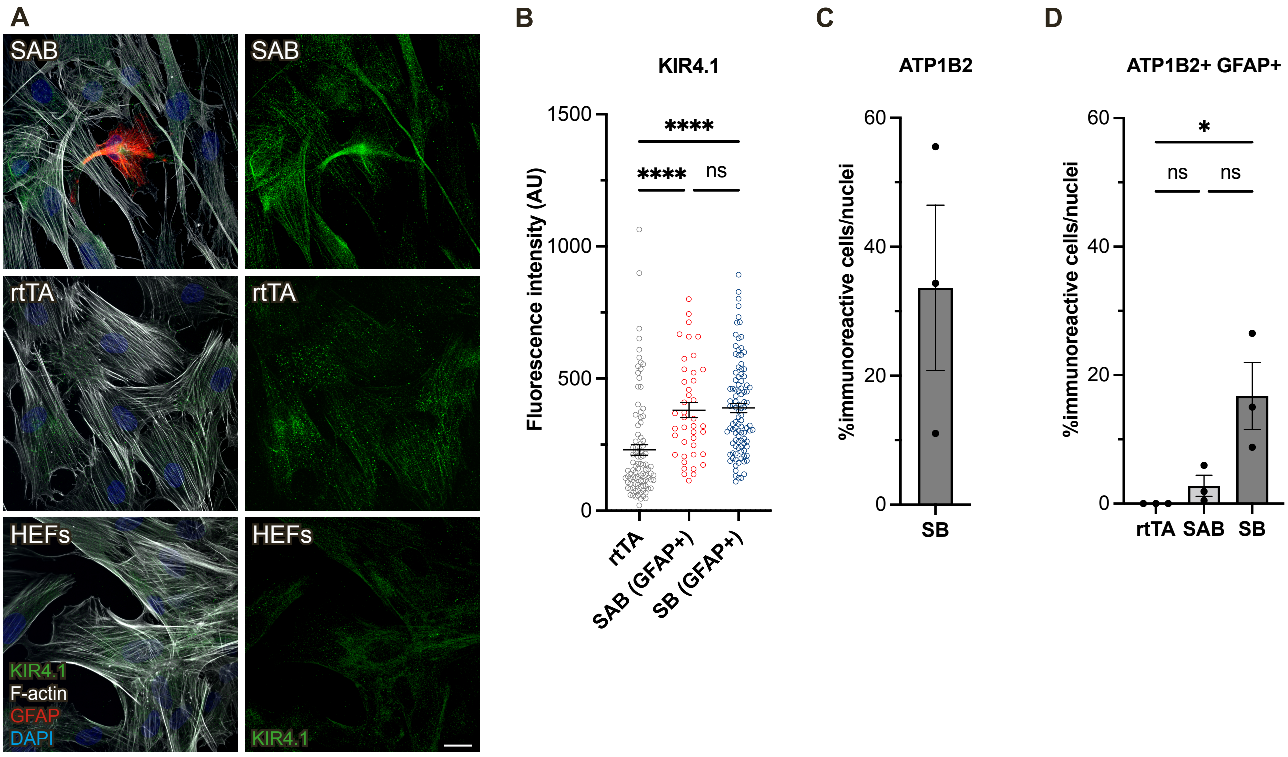

A
SAB
SAB
rtTA
rtTA
HEFs
HEFs
KIR4.1
F-actin
GFAP
DAPI
KIR4.1
B
KIR4.1
Fluorescence intensity (AU)
1500
1000
500
0
****
****
ns
rtTA
SAB (GFAP+)
SB (GFAP+)
C
ATP1B2
%immunoreactive cells/nuclei
60
40
20
0
SB
D
ATP1B2+ GFAP+
%immunoreactive cells/nuclei
60
40
20
0
*
ns
ns
rtTA
SAB
SB

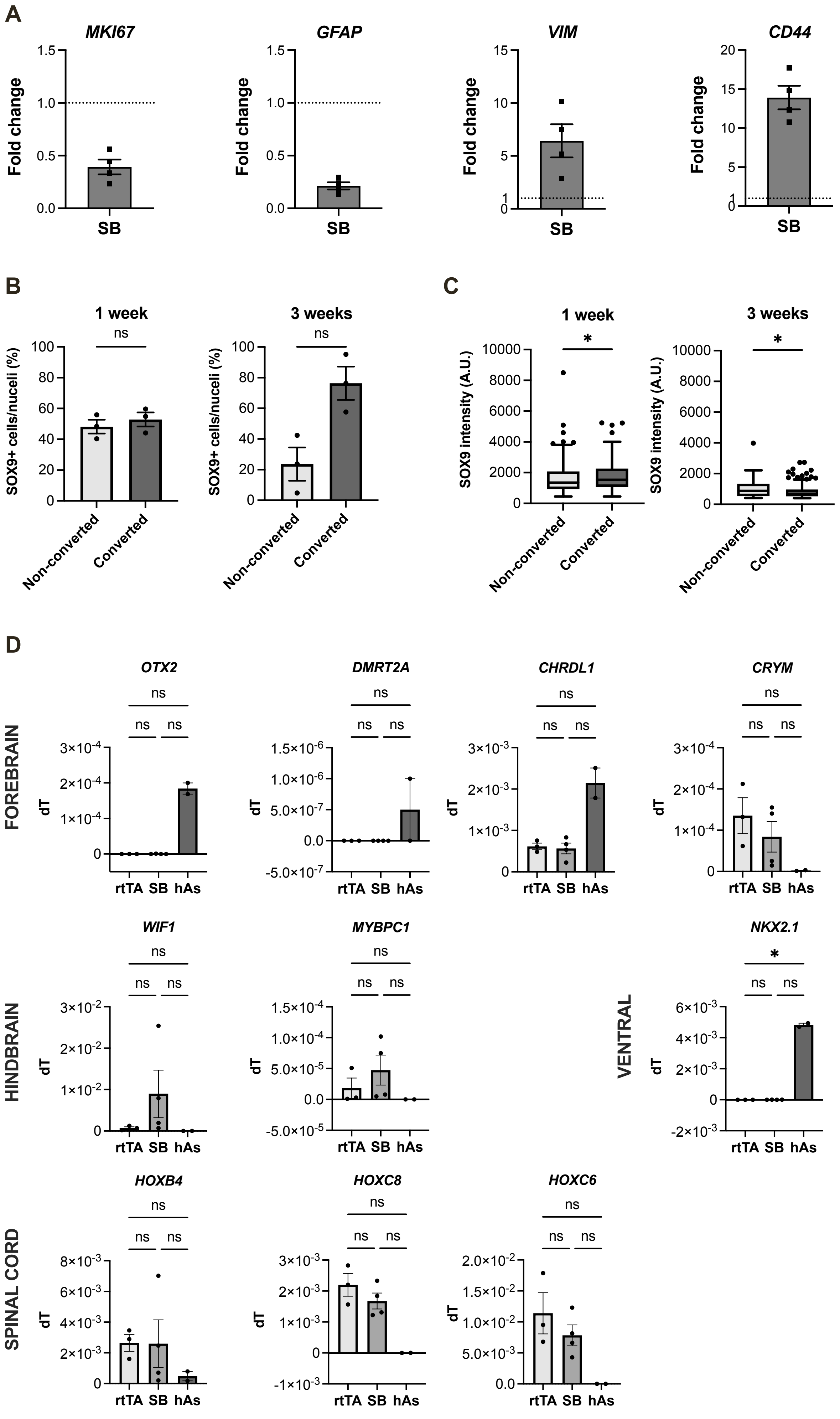
A
MKI67
GFAP
VIM
CD44
Fold change
SB
B
1 week
3 weeks
ns
SOX9+ cells/nuceli (%)
Non-converted
Converted
C
1 week
3 weeks
*
SOX9 intensity (A.U.)
Non-converted
Converted
D
FOREBRAIN
OTX2
DMRT2A
CHRDL1
CRYM
HINDBRAIN
WIF1
MYBPC1
VENTRAL
NKX2.1
SPINAL CORD
HOXB4
HOXC8
HOXC6
ns
*
dT
rtTA
SB
hAs

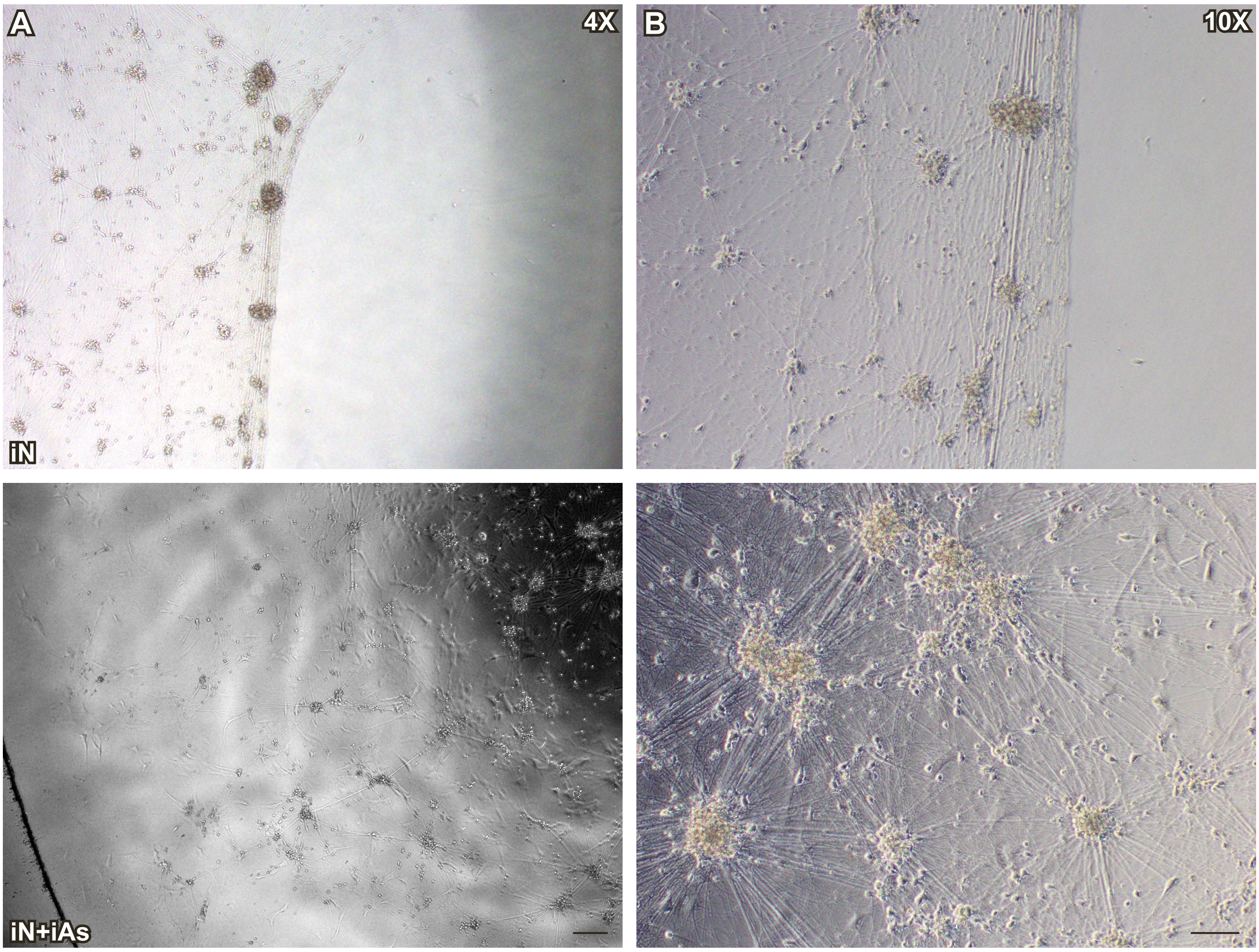
A
4X
B
10X
iN
iN+iAs

Supplemental Information

SUPPLEMENTAL FIGURES

Figure S1. Immunocytochemical analysis and biocytin spread in control HEFs. Related to Figure 2.
(A) Representative maximum intensity projection immunofluorescence images of ATP1B2 co-stained with GFAP and F-actin in rtTA-infected control HEFs (rtTA) and SAB-infected HEFs (SAB) at 5 weeks and untreated HEFs. **(B)** Quantification of ATP1B2 immunoreactive cells at 5 weeks related to total number of nuclei. **(C-D)** Representative maximum intensity projection immunofluorescence images of CX43 using two different antibodies in untreated HEFs and rtTA-infected control HEF at 5 weeks. (**E**) Gene expression of *GJA1* as determined by RT-qPCR shown relative to *GAPDH* in untreated HEFs, rtTA-infected control HEFs at 5 weeks and human fetal cortical astrocytes (hAs) (**F)** Example immunofluorescence image of biocytin injected rtTA-infected control HEFs. Arrowheads indicate cells with biocytin and thick full arrowhead biocytin-injected cell. Scale bar: 20 µm (A, C-D), 50 µm (F). Data are presented as mean±SEM of n=2-3 independent viral transduction experiments or RNA isolations done on separate days. Mann-Whitney (B) and Kruskal-Wallis (E) tests with significance level $p<0.05$ were performed for statistical analysis.

Figure S2. Transcription factor screen using *Nfia, Nfib* and *Sox9*. Related to Figure 4. (A) Representative immunofluorescence images of GFAP and S100B, 3 weeks after induction of *Nfia*, *Nfib* and *Sox9* either alone, in pairs or all three in combination. **(B)** Quantification of total number of nuclei in SAB-infected HEFs in serum containing media (SAB SERUM), SAB-infected HEFs in FGF2+Serum-free+SM media (SAB FGF) and SB-infected HEFs in FGF2+Serum-free+SM media (SB FGF) at 1 and 3 weeks. **(C)** KI67 expression related to number of nuclei. **(D)** KI67 expression in S100B and/or GFAP immunoreactive cells (converted cells). **(E)** Gene expression of *S100B*, *GFAP* and *DCN* as determined by RT-qPCR shown relative to *GAPDH* in rtTA-infected control HEFs and SAB- or SB-infected HEFs at 5 weeks. Scale bar: 20 µm (A). Data are presented as mean±SEM of n=3-4 independent viral transduction experiments. Friedman test (B-C) and Kruskal-Wallis (E) tests were performed for statistical analysis. *$p<0.05$.

Figure S3. SB-HEF-iAs conversion does not induce muscle or renal epithelia cells.
(A-B) Representative immunofluorescence images of MYOD and ANP co-stained with GFAP and S100B in SB-HEF-iAs at 5 weeks. Scale bar: 20 µm.

Figure S4. Additional characterization of SB-HEF-iAs. Related to Figure 4.
(A-B) Representative maximum intensity projection immunofluorescence images of AQP4 and ALDH1L1 co-stained with GFAP and F-actin of rtTA-infected control HEFs (rtTA), SAB-infected HEFs (SAB), SB-infected HEFs (SB), all at 5 weeks, and untreated HEFs. (**C-E)** Representative maximum intensity projection immunofluorescence images of EAAT1, EAAT2 and GS co-stained with GFAP and F-actin of rtTA-infected control HEFs (rtTA) at 5 weeks and untreated HEFs. (**F**) Absolute fluorescence intensity measurement of EAAT1, EAAT2 and GS staining in rtTA-infected control HEFs (rtTA), GFAP+ SAB-infected HEFs (SAB) and GFAP+ SB-infected HEFs (SB) at 5 weeks. **(G)** Representative maximum intensity projection immunofluorescence image of CX43 co-stained with GFAP and F-actin in SB-infected HEFs (SB) at 5 weeks. (**H)** Example immunofluorescence image of SB-HEF-iAs at 5 weeks showing spread of biocytin between S100B+ cell to S100B+ and GFAP+ cell. Arrowheads indicate cells with biocytin and thick full arrowhead biocytin-injected cell. Individual color levels adjusted for visibility (H). Scale bar: 50 µm (A, B, C, E, G, H). Data are presented as mean±SEM of n=72-211 cells from 3 independent viral transduction experiments. Kruskal-Wallis tests was performed for statistical analysis. *$p<0.05$. ****$p<0.0001$.

Figure S5. KIR4.1 immunocytochemistry, expression levels and ATP1B2 quantification. Related to Figure 4.
(A) Representative maximum intensity projection immunofluorescence images of potassium buffering protein KIR4.1 co-stained with GFAP and F-actin in rtTA-infected control HEFs (rtTA), SAB-infected HEFs (SAB) both at 5 weeks, and untreated HEFs. (**B**) Absolute intensity measurement KIR4.1 in rtTA-infected control HEFs (rtTA), GFAP+ SAB-infected HEFs (SAB) and GFAP+ SB-infected HEFs (SB) at 5 weeks. **(C)** Quantification of ATP1B2 expressing cells at 5 weeks related to total number of nuclei. **(D)** Quantification of ATP1B2 and GFAP co-expressing cells at 5 weeks related to total number of nuclei. Scale bar: 20 µm (A). Data are presented as mean±SEM of n=3-4 independent viral

transduction experiments done on separate days. Kruskal-Wallis tests were performed for statistical analysis. *p<0.05. ****p<0.0001.

Figure S6. Gene and transcription factor expression in SB-HEF-iAs. Related to Figure 5.
(A) Gene expression of *MKI67, GFAP, VIM* and *CD44* in SB-HEF-iAs at 5 weeks following 24 h treatment with TNFα, IL-1α and Cq1 as determined by RT-qPCR shown relative to untreated SB-HEF-iAs. **(B)** Quantification of SOX9 immunoreactive cells in S100B and GFAP negative cells (non-converted) and S100B and/or GFAP positive cells (converted) relative to number of nuclei in SB-transduced HEFs at 1 and 3 weeks. **(C)** Absolute fluorescence intensity of SOX9 immunoreactive cells in S100B and GFAP negative cells (non-converted) and S100B and/or GFAP positive cells (converted) in SB HEFs at 1 and 3 weeks. **(D)** Gene expression of forebrain, midbrain, ventral and spinal cord markers in rtTA-HEFs and SB-HEF-iAs at 5 weeks and human fetal cortical astrocytes (hAs) as determined by RT-qPCR shown relative to *GAPDH.* Data are plotted as mean$\pm$SEM of n=3-4 independent viral transduction experiments (A, B, D) or n=2 separate RNA isolations (hAs) (D). and median, 25th and 75th percentiles with Tukey whiskers of n=133-215 cells from 3 independent viral transduction experiments (C). Mann-Whitney test (B, C) and Kruskal-Wallis test (D) was performed for statistical analysis *p<0.05.

Figure S7. iN co-cultures with SB-HEF-iAs shows improved attachment to coverslips. Related to Figure 7.
(A-B) Brightfield images illustrating iN detachment in monocultures but not in co-cultures. Scale bar: 200 µm (A), 100 µm (B).

SUPPLEMENTAL TABLES

Table S1. Primary antibodies for immunocytochemistry.

Antigen	Dilution	Company	Catalog number
Rabbit anti-S100	1:400	Dako	Z0311
Rabbit anti-S100	1:1	Dako	GA504
Guinea pig anti-GFAP	1:500	Synaptic Systems	173004
Rabbit anti-GLT1	1:250	Invitrogen	701988
Rabbit anti-GLAST	1:200	Abcam	ab416
Rabbit anti-GS	1:1000	Abcam	ab49873
Rabbit anti-CX43	1:1000	Abcam	ab11370
Rabbit anti-CX43	1:100	Santa Cruz Biotechnology	sc9059
Rabbit anti-ALDH1L1	1:500	Abcam	ab87117
Rabbit anti-AQP4	1:100	Abcam	ab5971
Rabbit anti-KIR4.1	1:1000	Alomone labs	APC-035
Rabbit anti-ATP1B2	1:50	Atlas Antibodies	HPA010698
Mouse anti-β-TUBULIN III	1:500	Sigma-Aldrich	T8660
Chicken anti-MAP2	1:5000	Abcam	ab5392
Phallodin-TRITC	2.5 ug/ml	Sigma-Aldrich	P1951
Mouse anti-MYOD	1:250	BD Biosciences	554130
Mouse anti-Aminopeptidase N (ANP)	1:100	R&D Systems	MAB3815

Table S2. Secondary antibodies for immunocytochemistry.

Antigen	Dilution	Company	Catalog number
Donkey anti-rabbit AF488	1:500	Life Technologies	A21206
Donkey anti-mouse AF488	1:500	Life Technologies	A21202
Donkey anti-chicken AF488	1:500	Jackson Immunoresearch	703-545-155
Donkey anti-rabbit AF568	1:500	Life Technologies	A10042
Goat anti-guinea pig AF568	1:500	Life Technologies	A11075
Donkey anti-rabbit AF647	1:500	Life Technologies	A31573
Donkey anti-guinea pig AF647	1:500	Jackson Immunoresearch	706-605-148

Table S3. TaqMan assays used for RT-qPCR.

Gene	Assay ID
GAPDH	Hs02758991_g1
GJA1	Hs00748445_s1
S100B	Hs00902901_m1
GFAP	Hs00909233_m1
DCN	Hs00370384_m1
IL-6	Hs00174131_m1
CXCL10	Hs00171042_m1
CXCL8	Hs00174103_m1
CCL5	Hs00982282_m1
C3D	Hs00163811_m1
MKI67	Hs01032443_m1
CD44	Hs01075862_m1
VIM	Hs00958111_m1
MYBPC1	Hs00159451_m1
WIF1	Hs00183662_m1
HOXB4	Hs00256884_m1
HOXC8	Hs00224073_m1
HOXC6	Hs00171690_m1
OTX2	Hs00222238_m1
DMRT2A	Hs00294890_m1
CHRDL1	Hs01035484_m1
CRYM	Hs00157121_m1
NKX2.1	Hs00968940_m1

Table S4. TaqMan assays used for single cell gene expression analysis. Related to Figure 3.

Marks	Gene	Assay ID
House-keeping	*YWHAZ*	Hs03044281_g1
House-keeping	*GAPDH*	Hs02758991_g1
House-keeping	*UBC*	Hs00824723_m1
House-keeping	*ACTB*	Hs01060665_g1
Astrocytes	*SOX9*	Hs00165814_m1
Astrocytes	*NFIA*	Hs00325656_m1
Astrocytes	*NFIB*	Hs01029174_m1
Astrocytes	*ATF3*	Hs00231069_m1
Astrocytes	*ID4*	Hs00155465_m1
Astrocytes	*EZR*	Hs00931646_m1
Astrocytes	*SLC1A3*	Hs00188193_m1
Astrocytes	*RUNX2*	Hs01047973_m1
Astrocytes	*GPC4*	Hs00155059_m1
Fetal astrocytes	*NOTCH1*	Hs01062014_m1
Fetal astrocytes	*CNTFR*	Hs00181798_m1
Fetal astrocytes	*TNC*	HS01115665_m1
Fetal astrocytes	*TUBA1A*	Hs00362387_m1
Fetal astrocytes	*FABP7*	Hs00361426_m1
Fetal astrocytes	*NNAT*	Hs00193590_m1
Mature astrocytes	*KCNJ10*	Hs00158426_m1

Mature astrocytes	*ATP1B2*	Hs00155922_m1
Mature astrocytes	*ATP1A2*	Hs00265131_m1
Mature astrocytes	*ALDOC*	Hs00902799_g1
Mature astrocytes	*S100B*	Hs00902901_m1
Mature astrocytes	*IGFBP7*	Hs00266026_m1
Mature astrocytes	*SLC25A18*	Hs01017349_m1
Mature astrocytes	*SLC15A2*	Hs01113665_m1
Mature astrocytes	*SLC14A1*	Hs00998199_m1
Mature astrocytes	*F3*	Hs01076029_m1
Mature astrocytes	*HEPACAM*	Hs00404147_m1
Mature astrocytes	*MLC1*	Hs01126548_m1
Mature astrocytes	*FGFR3*	Hs00179829_m1
Mature astrocytes	*DIO2*	Hs00255341_m1
Mature astrocytes	*BMPR1B*	Hs01010965_m1
Mature astrocytes	*SLC4A4*	Hs00186798_m1
Fibroblasts	*DCN*	Hs00370384_m1
Fibroblasts	*ECM1*	Hs00189435_m1
Fibroblasts	*TSLP*	Hs00263639_m1
Fibroblasts	*TGFB1I1*	Hs00210887_m1
Fibroblasts	*VCAM1*	Hs01003372_m1
Proliferating cells	*MKI67*	Hs01032443_m1
Proliferating cells	*TOP2A*	Hs01032137_m1
Neural stem cells	*PAX6*	Hs00240871_m1
Neurons	*MYT1L*	Hs00903951_m1
Neurons	*SYP*	Hs00300531_m1
Oligodendrocytes	*OLIG2*	Hs00377820_m1
Oligodendrocytes	*MBP*	Hs00921945_m1

SUPPLEMENTAL EXPERIMENTAL PROCEDURES

Human tissue and cell culture

Human tissue was obtained from dead aborted human fetuses 6-9 week post-conception from Lund and Malmö University Hospitals. Developmental stage of the embryos was determined by crown-to-rump length and careful evaluation of external features of the embryos and internal features of the nervous system. Tissue was microdissected under a stereo-microscope (Leica) in ice-cold hibernation medium (Gibco). Human embryonic dermal fibroblasts (HEFs) were isolated after removal of the central nervous system, spinal ganglia and internal organs, residual tissue was transferred several times to a clean petri dish containing sterile cold hibernation medium to remove contaminant cells. The epidermis was then carefully removed by tearing the tissue with sterile forceps. Sub-dissected epidermis was washed several times with hibernation medium prior to enzymatic digestion with 0.25% Trypsin-EDTA (Sigma-Aldrich) at 37°C for 5 minutes and was manually triturated to reach a single cell suspension. Cells were plated onto 0.1% gelatin coated flasks (Sigma-Aldrich) in Fibroblast medium (DMEM, 10% FBS, 1% GlutaMAX, 1% NEAA and 1% Sodium Pyruvate, all from Gibco), passaged after reaching confluence with 0.25% Trypsin-EDTA and seeded in uncoated T25 or T75 flasks. HEFs were expanded for two passages, cryopreserved in 85% Fibroblast medium, 5% FBS and 10% DMSO (Sigma-Aldrich) and used for direct conversion experiments at passage 3-7. HEFs used for immunocytochemical analysis were after thawing expanded one passage, seeded on 0.167 mg/ml Matrigel-coated glass coverslips and fixed after 3 days.

Human fetal cortical astrocytes (hAs) were derived as previously described (Miskinyte et al., 2018) from fetuses 7-9 week post-conception and expanded in poly-D-lysine (Sigma-Aldrich)/ fibronectin (Life Technologies) coated flasks in Fetal astrocyte medium (DMEM-F12, 10% FBS 1% GlutaMAX and 1% N-2, all from Gibco). hAs were used at passage 5-8. hAs used for immunocytochemical analysis were seeded on 0.167 mg/ml Matrigel-coated glass coverslips and fixed after 3-4 days.

Postnatal fibroblasts were initially expanded according to the manufacturer instructions after which they were expanded and cryopreserved as described for HEFs.

Vectors and viral production

FUW-rtTA (Addgene #20342) and tetO-Ascl1-Puromycin (Addgene #97329) constructs were from AddGene. TetO-FUW-Sox9-Puromycin (Addgene #117269), tetO-FUW-Nfia-Blasticidin (Addgene #117270) and tetO-FUW-Nfib-Hygromycin (Addgene #117271) were generated as described (Canals et al., 2018). TetO-Ngn2-Hygromycin was a kind gift from Dr. M.Mall DFKZ Heidelberg. Lentiviral particles were produced as previously described (Quist et al., 2021).

Direct conversion

Selection antibiotics were titrated and following concentration used: *Nfia* alone blasticin 4 µg/ml, *Nfib* alone 50 µg/ml hygromycin, *Sox9* alone 1 µg/ml puromycin, *Sox9+Nfia* 1 µg/ml blasticin and 0.5 µg/ml puromycin, *Sox9+Nfib* 1 µg/ml puromycin and 50 µg/ml hygromycin, *Nfia+Nfib* 2 µg/ml blasticin and 50 µg/ml hygromycin and *Sox9+Nfia+Nfib* 1 µg/ml blasticin, 0.5-1 µg/ml puromycin and 25-100 µg/ml hygromycin.

Human embryonic and postnatal fibroblasts were seeded in 0.083 mg/ml Matrigel-coated 24-well plates (50,000 or 20 000 cells/well respectively) or 6-well plates (200,000 or 80,000 cells/well respectively) on day -2. For rtTA-infected controls half the number of cells were seeded due to their rapid proliferation without selection and rtTA-HEFs cultured in 6-well plates were split 1:6 using 0.25% Trypsin-EDTA at day 9-10, collected with FGF medium supplemented with 0.1% BSA (Gibco) and seeded in new Matrigel-coated 6-well plates.

For serum-containing condition the Fibroblast medium was changed every second or third day until day 21-23.

For Serum-free condition (1:1 DMEM-F12, HEPES:Neurobasal, 1% N-2, 1% GlutaMAX, 1% Sodium Pyruvate, 5 µg/ml N-Acetyl-L-cysteine (Sigma-Aldrich) and 5 µg/ml Heparin-binding EGF-like growth factor (Sigma-Aldrich)) and Serum-free + SM condition (10 ng/ml CNTF (Peprotech), 40 ng/ml hLIF (Peprotech), 40ng/ml BMP4 (Peprotech) and 500 µg/ml dbcAMP (Sigma-Aldrich)), the Serum-free medium (+SM) was gradually introduced after selection periods were finished at day 6. Thereafter, half of the culture medium was changed every second or third day.

For electrophysiological recordings, biocytin-injections and immunocytochemical analysis at day 37-42, cells were dissociated with accutase (Gibco) and seeded on 0.167 mg/ml Matrigel-coated glass coverslips (5,000-20,000 cells/coverslip) 4-7 days before analysis.

Astrocytic identity of obtained cells was analyzed at day 21-23 and day 33-42.

Immunocytochemistry

Cells were washed with KPBS, fixed in 4% paraformaldehyde in KPBS for 20 min at room temperature (RT) and washed with KPBS. Blocking and permeabilization was performed with 0.025 % Triton X-100 in KPBS containing either 5% normal donkey serum (NDS) (Millipore) or 2.5% NDS and 2.5% normal goat serum (NGS) (Millipore) (blocking solution) for 1 h at RT. Primary antibodies were applied overnight at 4°C in blocking solution. Cells were washed two times with 0.025 % Triton X-100 and once with blocking solution for 5 min each. Secondary antibodies and 1 µg/ml Hoechst 33342 (Invitogen) were applied for 2 h at RT in blocking solution. For F-actin staining Phallodin-TRITC were applied with secondary antibodies. Cells were washed with 0.025% Triton X-100 for 5 min, if DAPI (Sigma-Aldrich) was used for nuclear staining it was included at 0.5 µg/ml in this wash, followed by two washes with KPBS for 5 min after which wells were filled with KPBS containing 0.1% sodium azide (Sigma-Aldrich) and coverslips washed once with MilliQ and mounted on glass slides using Dabco:PVA. Primary and secondary antibodies used are listed in Table S1 and S2.

Image acquisition, processing and quantification

Images were acquired using Olympus BX61 or Olympus IX73 using CellSens software or Zeiss LSM 780 confocal microscope using Zen software. HDR was used for acquisition of punctate protein expression. Brightness and contrast were adjusted for entire images, Individual color levels only when needed to reflect what was observed at the microscope and if so indicated in figure legends.

Images for quantifications were acquired using Olympus IX51 and IX73 inverted microscopes with a 20X magnification and CellSens software. The number of S100B and GFAP immunoreactive cells were counted in 10-20 randomly selected fields of view in one to two wells of each independent viral transduction experiments. Exposure times were set to be as similar as to the intensity as when viewed directly with the microscope and were kept constant for acquisition of all wells in each infection and staining. Images of secondary antibody only controls were acquired with the same exposure settings used during each acquisition session. Quantification of nuclei and immunoreactive cells were performed in ImageJ. Total number of nuclei and immunoreactive cells in each well was quantified based on the area of the wells and the fields of view area. For ATP1B2 quantifications at least 100 cells for each independent viral transduction were counted.

The fluorescence intensity measurements were performed on at least three independent biological replicates for each experimental condition. For each replicate, all images were acquired using constant parameters. The analysis was performed as absolute fluorescence intensity measurement for cells identified as GFAP positive in SAB-HEF-iAs and SB-HEF-iAs conditions in at least four different fields randomly acquired from the culture. Imaging corrections were performed for both camera noise and for field illumination for all acquired fields.

Glutamate uptake assay

rtTA-infected, SAB or SB-infected HEFs or human adult fibroblasts were cultured in 6-well plates and analyzed at 33-34 days after induction. Human fetal astrocytes used as controls were cultured on PDL- and fibronectin coated T25 flasks or Matrigel-coated Ibidi dishes. Cells were washed three times with HBSS (Mg2+ and Ca2+) (Gibco) after which 125 μM Glutamic acid (Sigma-Aldrich) in HBSS was applied. Samples were collected after 30 min and kept at 4°C until analysis. Amount of glutamate was measured using a Fluorometric kit (Abcam ab138883). Measured absorbances for standards and samples were corrected using a blank sample measurements and HBSS respectively. Glutamate uptake was determined by comparing the amount of glutamate in the media to the initial concentration. All standards and samples were measured in two technical replicates.

Electrophysiology

For whole cell patch-clamp recordings, coverslips were transferred to the recording chamber and constantly perfused with carbogenated artificial cerebrospinal fluid (ACSF), containing (in mM): 119 NaCl, 2.5 KCl, 1.3 $MgSO_4$, 2.5 $CaCl_2$, 26 $NaHCO_3$, 1.25 NaH_2PO_4, and 11 glucose (pH ~7.4, osmolarity ~305 mOsm). Patch pipette was filled with internal solution containing (in mM): K-gluconate 122.5, KCl 17.5, NaCl 8, KOH-HEPES 10, KOH-EGTA 0.2, MgATP 2, and Na3GTP 0.3 (pH ~7.2, 295 mOsm). Average pipette tip resistance was ~4-5 MΩ and recordings were done at 34°C. Pipette current was corrected online before gigaseal formation while fast capacitive currents were compensated for during cell-attached configuration. All recordings were done using a HEKA double patch EPC10 amplifier (HEKA Elektronik, Lambrecht, Germany) and sampled at 10 KHz. PatchMaster software was used for data acquisition and Fitmaster, IgorPro and NeuroMatic (Rothman and Silver, 2018) for offline analysis.

Single cell recordings of SAB/SB-HEF-iAs were performed at day 37-42 and compared to untreated HEFs and human fetal cortical astrocytes. 5,000-10,000 cells were plated on glass coverslips 3-9 days before patch clamp recordings. Only S100B+ or GFAP+ SAB/SB-HEF-iAs, as identified with biocytin co-labeling or SAB/SB-HEF-iAs with a clear astrocytic morphology were included in analysis. SAB-HEF-iAs from two independent viral transduction and SB-HEF-iAs from four independent viral transductions were used.

Resting membrane potential (RMP) was measured in current clamp mode at 0 pA immediately after establishing whole-cell configuration. Series resistance and input resistance (Ri) were calculated from a 5, or 10 mV pulse and monitored throughout the experiment. Whole-cell currents were induced in voltage-clamp mode either by delivering voltage steps in 10 mV increments for 200 milliseconds (ms) at a holding potential of −80 mV, or a protocol that, from the holding potential of −60 mV, stepped the membrane potential to −100 mV for 200 ms before a 100 ms long depolarizing ramp to +120 mV. To minimize recording errors, series resistance was compensated to 60-80% and recordings with leak currents >100pA were excluded. Moreover, quality of recording was monitored with a test pulse throughout the recording.

Electrophysiological properties of iNs were recorded at day 45-52. RMP, Ri and series resistance was measured similar to above described. Ability to generate action potential (AP) was determined by 500 ms square depolarizing current step injections at RMP, with 10 pA increments and ramp injection of 1 second depolarizing current, that was also used to determine action potential threshold. AP amplitude was measured from threshold to peak, the half AP amplitude width was determined as the time between the rising and decaying phase of the AP measured at half the amplitude of the AP, and the afterhyperpolarization (AHP) amplitude was determined as the difference between the AHP peak and the AP threshold. Whole-cell sodium and potassium currents were observed in voltage-clamp mode at a holding potential of −70 mV and 200ms voltage steps were delivered in 10 mV increments.

Biocytin labelling
To confirm identity of recorded cells for astrocyte experiments and visualize diffusion through gap junctions individual cells were filled with biocytin (1-3 mg/ml, Biotium in internal solution) using a patch pipette during electrophysiology recordings. To detect biocytin post hoc, Alexa Fluor 568 conjugated Streptavidin (ThermoFisher Scientific) diluted 1:500 in 0.025% TKPBS with 5% NDS was added for 1h at RT and co-stained with S100B or GFAP antibody. SAB-HEF-iAs from two independent viral transductions and SB-HEF-iAs from four independent viral transductions were used.

Gene Expression
For RT-qPCR analysis cells were at day 33-34 washed once with DPBS (-/-) (Gibco) before RNA was collected and purified using RNeasy® Mini Kit (Qiagen). DNase I (Qiagen) digestion was performed to prevent DNA contamination. RNA concentration and purity were determined with a Nanodrop ND-1000 Spectrophotometer (Saveen & Werner). cDNAs were prepared using qScript cDNA Synthesis Kit (Quanta Biosciences). For each sample two to three technical replicates were performed as well as a no reverse transcriptase reaction (NRT) to exclude DNA contamination. 50 ng cDNA were used for each qPCR reaction and was performed using TaqMan Universal PCR Master Mix and TaqMan Gene Expression Assays (both ThermoFisher Scientific) (Table S3) on an iQ5 real-time PCR detection system (Biorad). For each TaqMan Assay a no-template control was included. Data are expressed relative to the house-keeping gene GAPDH using the ΔCt method.

Single-cell RT-qPCR analysis was done as previously described (Miskinyte et al., 2017). Genes were selected from multiple sources based on reported expression in fibroblasts, fetal and mature astrocytes, neural stem cells, neurons and oligodendrocytes (complete list in Table S4) (Allen et al., 2012; Khazaei et al., 2018; Molofsky and Deneen, 2015; Philippeos et al., 2018; Sloan et al., 2017; Tiwari et al., 2018; Uhlén et al., 2015; Zhang et al., 2016). In brief, single viable (based on DRAQ7 (Abcam) staining) rtTA-infected and SAB-infected HEFs at day 21-23 from four independent viral transductions and human fetal astrocytes were sorted in a FACSAria II (BD Biosciences) to individual wells of 96-well plates containing lysis buffer. Linearity controls (0-, 10- and 20-cells) were included in each plate. Plates were snap frozen and stored at -80°C until analysis. RT-PCR pre-amplification were done using CellsDirect™ One-Step qRT-PCR Kit (ThermoFisher Scientific) and TaqMan assays (ThermoFisher Scientific). XenoRNA™ Control (TaqMan™ Cells-to-CT™ Control Kit, ThermoFisher Scientific) and 3 NRT controls were included to control for presence of RT-PCR inhibitors and DNA contamination respectively. cDNA levels were determined using the BioMark dynamic array system (Fluidigm). Amplification curves were manually inspected in the Fluidigm Real-Time PCR Analysis software to ensure proper loading and reading of the chips. Data was analyzed with the Auto (Global) Ct threshold method using a Linear (Derivate) baseline correction and quality threshold set at 0.6 and further filtered and processed using the SCExV webtool (Lang et al., 2015). Cells with non-detectable (Ct>25) levels of house-keeping gene UBC and genes with detectable levels (Ct<25) in NRT controls or presence of failed amplification curves were excluded as well as house-keeping genes, xRNA and 0-, 10- and 20-cells controls. Data was normalized to the median Ct of each cell, Ct-values inverted, z-scored normalized by gene, non-detectable gene expression was artificially set to the minimum of the z scored values minus 1 and hierarchical clustering analysis performed using ward.D algorithm to clustered based on correlation distance. Groups were manually colored based on condition (rtTA-infected HEFs, SAB-infected HEFs and human fetal astrocytes respectively) to generate heat map, violin plots and DDRTree.

Cytokine stimulation
30 ng/mL TNFα (R&D Systems), 3 ng/mL IL-1α (Sigma-Aldrich) and 400 ng/mL C1q (MyBioSource) were applied in a full media change. 24 hours after stimulation cells were either fixed for immunocytochemistry or RNA was collected for RT-qPCR.

Calcium imaging
rtTA-HEFs, SB-HEF-iAs and human fetal astrocytes were cultured in 6w plates and imaged in DMEM/F12 without phenol red containing 25mM HEPES (both from ThermoFisher Scientific). Cells were washed once with DMEM/F12, loaded with 2 µM Fluo4 (Life Technologies) prepared in Pluronic Acid (Invitrogen) in DMEM/F12 for 30 min at 37 C after which a full media change to 2 ml DMEM/F12 was performed. Imaging was done using Olympus IX73 microscope with a 10X objective at 1Hz for 5 min. Baseline recording were done for 60 s after which 500 µl 90 µM ATP was added next to the imaging field. Data was analyzed using Fiji-ImageJ software. Ca^{2+} signals are presented as relative fluorescence changes (dF/F0) from specified regions of interest (ROIs). Single ROIs were manually selected based on overlays of Fluo-4 fluorescence maximum intensity projection and brightfield

images of the recorded field. Data was corrected for background intensity levels at each time point based on the signal detected in an area not containing any cells. Calcium elevation events were detected with thresholds of 3 times the standard deviation from baseline, which was set as the time interval preceding the stimulus. In experiments that presented spontaneous Ca^{2+} oscillations in the baseline time window, F0 was calculated in the time interval in between spontaneous transients. For post hoc identification of S100B and GFAP expressing cells ROI selection was manually done on immunocytochemical images of recorded fields.

Generation of iNs and iAs/iN co-cultures

HEF-iNs and SB-HEF-iAs were generated in parallel using the same starting population of HEFs. Co-cultures were set up at day 9-10.

iNs were generated by transducing HEFs with rtTA, tetO-Ascl1-Puromycin and tetO-Ngn2-Hygromycin following the same procedure as described for iAs until day 1 when a 3-day selection period with puromycin (1 µg/ml) and 5-day selection period with hygromycin (50 µg/ml) was started. On day 3, 1:1 Fibroblast medium and Early iN medium (1:1 DMEM-F12, HEPES:Neurobasal, 1% GlutaMAX, 2% B-27 and 1% N-2 supplemented with 1 µg/ml mouse laminin (Gibco), 400 µg/ml db-cAMP, 150 µg/ml Noggin (Peprotech), 10 µM SB431542 (Peprotech), 3 µM CHIR99021 (Sigma-Aldrich), 5 µM Forskolin (Peprotech), 0.5 µM LDN-193189 (Selleck) and 0.5 µM A83-1 (StemCell Technologies) were added. At day 4 a full medium change to Early iN medium was performed after which half of the medium was changed every 2nd to 3rd day. At day 9-10 HEF-iN and SB-HEF-iAs were seeded together. Cells were washed with DPBS (-/-), dissociated using accutase containing 100 U/ml DNase I (Roche), collected with FGF medium/Early iN containing 0.1% BSA and 100U/ml DNase I and pelleted by centrifugation (5 min, 300 rcf). 10,000-20,000 SB-HEF-iAs were seeded on poly-D-lysine/laminin-coated glass coverslips by applying cell suspensions as a drop and allowed to attach after which HEF-iNs were passed through 40 µm strainer and 60,000-80,000 HEF-iNs were seeded in a 1:1 FGF medium:Early iN medium alone or on top of SB-HEF-iAs as a drop. Once cells had attached medium were filled to 500 µl. The day after a full medium change were performed after which half medium changes were done every second to third day. At day 11, FGF medium was replaced with Serum-free medium supplemented with 10 ng/ml CNTF, 10 ng/ul BMP4 and 500 µg/ml dbcAMP and at day 18 Early iN medium was replaced by Late iN medium (1:1 DMEM-F12, HEPES:Neurobasal, 1% GlutaMAX, 2% B-27 and 1% N-2 supplemented with 1 µg/ml mouse laminin, 500 µg/ml db-cAMP, 10ng/ml GDNF (Peprotech) and 10 ng/ml NT3 (Peprotech). If signs on detachment were observed 10 µg/ml laminin were added when changing medium.

Statistical analysis

Statistical analysis was performed using GraphPad Prism (version 7.02-9.2.0). Statistical significance was determined by the following statistical tests; S100B and GFAP quantifications two-tailed paired t-test and Kruskal-Wallis test, ATP1B2 and SOX9 quantifications Mann-Whitney test, RT-qPCR data Mann-Whitney test and Kruskal-Wallis test, glutamate uptake Kruskal-Wallis test and two-tailed Wilcoxon matched-pairs rank test, cell number quantifications and KI67 quantifications Friedman test, fluorescence intensity measurements and calcium signaling data Kruskal-Wallis test. For electrophysiological recordings of uninfected HEFs, SAB-HEF-iAs, SB-HEF-iAs and hAs Kruskal-Wallis tests were performed except for the current/voltage (I/V) curve where statistical significance was analyzed using multiple unpaired t-tests. For electrophysiological recordings of iNs unpaired t-tests were performed. All Kruskal-Wallis tests were followed by Dunn's multiple comparison test. Significance level of 0.05 were used in all statistical tests.

SUPPLEMENTAL REFERENCES

Allen, N.J., Bennett, M.L., Foo, L.C., Wang, G.X., Chakraborty, C., Smith, S.J., and Barres, B.A. (2012). Astrocyte glypicans 4 and 6 promote formation of excitatory synapses via GluA1 AMPA receptors. Nature *486*, 410-4. 10.1038/nature11059.

Canals, I., Ginisty, A., Quist, E., Timmerman, R., Fritze, J., Miskinyte, G., Monni, E., Hansen, M.G., Hidalgo, I., Bryder, D., et al. (2018). Rapid and efficient induction of functional astrocytes from human pluripotent stem cells. Nat Methods *15*, 693-6. 10.1038/s41592-018-0103-2.

Khazaei, N., Rastegar-Pouyani, S., O'Toole, N., Wee, P., Mohammadnia, A., and Yaqubi, M. (2018). Regulating the transcriptomes that mediate the conversion of fibroblasts to various nervous system neural cell types. J Cell Physiol *233*, 3603-14. doi:10.1002/jcp.26221.

Lang, S., Ugale, A., Erlandsson, E., Karlsson, G., Bryder, D., and Soneji, S. (2015). SCExV: a webtool for the analysis and visualisation of single cell qRT-PCR data. BMC Bioinformatics *16*, 320. 10.1186/s12859-015-0757-z.

Miskinyte, G., Devaraju, K., Gronning Hansen, M., Monni, E., Tornero, D., Woods, N.B., Bengzon, J., Ahlenius, H., Lindvall, O., and Kokaia, Z. (2017). Direct conversion of human fibroblasts to functional excitatory cortical neurons integrating into human neural networks. Stem Cell Res Ther *8*, 207. 10.1186/s13287-017-0658-3.

Miskinyte, G., Gronning Hansen, M., Monni, E., Lam, M., Bengzon, J., Lindvall, O., Ahlenius, H., and Kokaia, Z. (2018). Transcription factor programming of human ES cells generates functional neurons expressing both upper and deep layer cortical markers. PLoS One *13*, e0204688. 10.1371/journal.pone.0204688.

Molofsky, A.V., and Deneen, B. (2015). Astrocyte development: A Guide for the Perplexed. Glia *63*, 1320-9. 10.1002/glia.22836.

Philippeos, C., Telerman, S.B., Oulès, B., Pisco, A.O., Shaw, T.J., Elgueta, R., Lombardi, G., Driskell, R.R., Soldin, M., Lynch, M.D., et al. (2018). Spatial and Single-Cell Transcriptional Profiling Identifies Functionally Distinct Human Dermal Fibroblast Subpopulations. J Invest Dermatol *138*, 811-25. 10.1016/j.jid.2018.01.016.

Quist, E., Ahlenius, H., and Canals, I. (2021). Transcription Factor Programming of Human Pluripotent Stem Cells to Functionally Mature Astrocytes for Monocultures and Cocultures with Neurons. In Methods Mol Biol, H. Ahlenius, ed. (New York, NY: Humana), pp. 133-48.

Rothman, J.S., and Silver, R.A. (2018). NeuroMatic: An Integrated Open-Source Software Toolkit for Acquisition, Analysis and Simulation of Electrophysiological Data. Front Neuroinform *12*, 14. 10.3389/fninf.2018.00014.

Sloan, S.A., Darmanis, S., Huber, N., Khan, T.A., Birey, F., Caneda, C., Reimer, R., Quake, S.R., Barres, B.A., and Pasca, S.P. (2017). Human Astrocyte Maturation Captured in 3D Cerebral Cortical Spheroids Derived from Pluripotent Stem Cells. Neuron *95*, 779-90. 10.1016/j.neuron.2017.07.035.

Tiwari, N., Pataskar, A., Péron, S., Thakurela, S., Sahu, S.K., Figueres-Oñate, M., Marichal, N., López-Mascaraque, L., Tiwari, V.K., and Berninger, B. (2018). Stage-Specific Transcription Factors Drive Astrogliogenesis by Remodeling Gene Regulatory Landscapes. Cell Stem Cell *23*, 557-71. 10.1016/j.stem.2018.09.008.

Uhlén, M., Fagerberg, L., Hallström, B.M., Lindskog, C., Oksvold, P., Mardinoglu, A., Sivertsson, Å., Kampf, C., Sjöstedt, E., Asplund, A., et al. (2015). Proteomics. Tissue-based map of the human proteome. Science *347*, 1260419. 10.1126/science.1260419

Zhang, Y., Sloan, S.A., Clarke, L.E., Caneda, C., Plaza, C.A., Blumenthal, P.D., Vogel, H., Steinberg, G.K., Edwards, M.S., Li, G., et al. (2016). Purification and Characterization of Progenitor and Mature Human Astrocytes Reveals Transcriptional and Functional Differences with Mouse. Neuron *89*, 37-53. 10.1016/j.neuron.2015.11.013.
